# Microextraction of *Reseda luteola*-Dyed Wool and Qualitative Analysis of Its Flavones by UHPLC-UV, NMR and MS

**DOI:** 10.3390/molecules26133787

**Published:** 2021-06-22

**Authors:** Elbert van der Klift, Alexandre Villela, Goverdina C. H. Derksen, Peter P. Lankhorst, Teris A. van Beek

**Affiliations:** 1 Laboratory of Organic Chemistry, Wageningen University, Stippeneng 4, 6708 WE Wageningen, The Netherlands; ejcvanderklift@gmail.com (E.v.d.K.); alexandre.villela@naturalproductschemistry.com (A.V.); 2 Research Group Biobased Products, Avans University of Applied Sciences, P.O. Box 90.116, 4800 RA Breda, The Netherlands; gch.derksen@avans.nl; 3 DSM, Biotechnology Center, A. Fleminglaan 1, 2613 AX Delft, The Netherlands; peter.lankhorst@planet.nl

**Keywords:** weld, *Reseda luteola* L., natural yellow dye, μ-analysis, micro-extraction of wool, UHPLC, fingerprinting, flavones, NMR, structure elucidation

## Abstract

Detailed knowledge on natural dyes is important for agronomy and quality control as well as the fastness, stability, and analysis of dyed textiles. Weld (*Reseda luteola* L.), which is a source of flavone-based yellow dye, is the focus of this study. One aim was to reduce the required amount of dyed textile to ≤50 μg for a successful chromatographic analysis. The second aim was to unambiguously confirm the identity of all weld flavones. By carrying out the extraction of 50 μg dyed wool with 25 μL of solvent and analysis by reversed-phase UHPLC at 345 nm, reproducible chromatographic fingerprints could be obtained with good signal to noise ratios. Ten baseline separated peaks with relative areas ≥1% were separated in 6 min. Through repeated polyamide column chromatography and prepHPLC, the compounds corresponding with the fingerprint peaks were purified from dried weld. Each was unequivocally identified, including the position and configuration of attached sugars, by means of 1D and 2D NMR and high-resolution MS. Apigenin-4′-*O*-glucoside and luteolin-4′-*O*-glucoside were additionally identified as two trace flavones co-eluting with other flavone glucosides, the former for the first time in weld. The microextraction might be extended to other used dye plants, thus reducing the required amount of precious historical textiles.

## 1. Introduction

Since the stone age, mankind has dyed his clothes for decoration. Later also rugs and tapestries were dyed. For this purpose, minerals, insects, and especially plant extracts have been used. The most used dye plants in Europe throughout the ages include madder (*Rubia tinctorum* L.), weld (*Reseda luteola* L.) and woad (*Isatis tinctoria* L.) [1]. During the second half of the 19th century, natural dyes were replaced by cheaper, synthetic dyes. However, in recent times for various reasons there is a renewed interest in natural dyes. To facilitate the re-introduction of natural dyes, over the last 25 years many papers have appeared, for instance on screening methods for agronomic studies of dye plants [2,3], the fastness of dyed textiles [4], the colorimetric properties of dye plants and their individual constituents [5], mild extraction methods for textiles [6,7,8], degradation of dyes [9,10,11], safety of natural dyes [12], the development of new production routes [13], and analysis of dyed textiles [1,14]. Concerning the latter topic, natural dye analysis is of great importance for historians and conservators. Detailed knowledge about natural dyes used in old garments and tapestries can provide answers concerning the plant(s) used for dyeing, dyeing practices, age, origin, original colour, and trade routes. It can also help to shed light on previous restorations and help future restorations [1].

As historic textiles are precious, non-destructive analysis is preferred. Fibre optics reflectance spectroscopy (FORS) for the detection of dye molecules in the visible wavelength spectrum is such a technique. Unfortunately, the discriminatory power is limited and moreover the method is not suitable for distinguishing between different yellow dyes [15,16,17]. Surface-enhanced Raman scattering (SERS) has been used as a micro invasive technique [18,19]. SERS has also been coupled on-line with HPLC for greater resolution [20]. This methodology is better at distinguishing between yellow dyes. Jurasekova et al. have shown that different ratios of the flavones luteolin and apigenin generate different spectra [18]. A disadvantage of this method is that the textile has to be coated with silver nanoparticles.

Still the most powerful—albeit destructive—technique to obtain highly detailed knowledge on which dye plants have been used is HPLC after the extraction of one or more dyed threads. Each plant has its own unique blend of major and minor dye molecules and this fingerprint is thus specific even if some compounds are partially the same. Analyses can become more complex when significant decomposition has occurred. A prerequisite for a successful analysis is the intact isolation of all dyes. Until fairly recently strong mineral acids were employed to release dyes [9,21,22], e.g., from metals used for mordanting. Unfortunately, this leads to the hydrolysis of glycosidic dyes, such as those present in weld, and then no representative fingerprint is obtained. However, much progress has been made in developing milder yet efficient extraction methods [6]. Formic acid or EDTA instead of HCl provide higher recovery and glycosidic dyes remain intact. Moreover, other acids, such as oxalic acid, citric acid, or trifluoroacetic acid, have been proposed [23,24].

Detection after the HPLC separation can be by UV-Vis or UV-Vis followed by MS. The amount of material typically taken for HPLC analysis varies from 0.1-3.9 mg with 0.2 mg as average [1,6,7,9,21,22,23,24,25,26,27,28,29]. Obviously, the less material is needed, the better. On the other hand, analyte quantities are minute. Rosenberg estimated that typically less than 1 mg of dye is present per gram of fabric [7] and this estimate is in good correspondence with actual measurements on pure compounds [5]. This translates to 50 ng per 50 μg of wool. Even with 100% extraction efficiency—Willemen et al. have shown it might actually be <50% [5]—this equals, with a final sample volume of 125 μL and a 10 μL injection, about 4 ng on column. For a 1% component this amounts to only 40 pg. Thus, concentrations should be as high as possible. Earlier we have shown that by using short UHPLC columns for dye substances significant time reduction and sensitivity gains are possible [30]. Since then, UHPLC has been used for dye separations by several other groups [27,31,32]. Thus, the first aim was to investigate if it was possible by combining miniaturisation of the extraction step and using a modern UHPLC for the separation and detection to obtain clear chromatograms while using significantly less than 0.1 mg of textile. Modern UV detectors have become significantly more sensitive with femtogram (fg) limits of detection (LOD) [33]. Weld-dyed wool was taken as test material.

Upon going through assigned weld chromatograms in the literature, different assignments were found, and not always all stereochemical features of the glycosides were clear. Although ESI-MS is a powerful tool, it is less suited to assign where sugars are attached to the aglycone or to determine the configuration of sugars (α or β). Thus, several partial identifications of weld flavone mono and diglucosides have been reported after LC-MS studies [25,26,28]. The most detailed studies so far are those by Moiteiro et al., Marques et al., Mantzouris et al. and Otłowska et al. [24,26,28,34]. In contrast, the study by Burger is confusing with the major peaks reported as unknown [35]. Further, it is unclear where exactly luteolin-4′-*O*-glucoside elutes. According to Otlowska et al., it elutes just prior to luteolin while Moiteiro et al. place it before apigenin-7-O-glucoside [28,34]. Later, the group of Gaspar switched the assignment of the luteolin-3′ and 4′-*O*-glucosides [36]. The study by Serrano et al. is confusing as luteolin-3′,7-di-*O*-glucoside **3**, apigenin-7-*O*-glucoside **5**, and apigenin **9** are each assigned to two different peaks in the chromatogram, which is plainly impossible [32]. Moreover, monoglucosides elute before diglucosides [32], which is highly unlikely in a reversed-phase system. To arrive at an unambiguously assigned weld fingerprint these knowledge gaps have to be filled. Thus, the second aim was to carry out a preparative extraction of weld plant material to enable the isolation and purification of all flavonoids corresponding with peaks having a normalised peak area ≥1% in the weld fingerprint chromatogram. This was to be followed by the unequivocal identification of each isolated compound by means of 1-dim and 2-dim NMR and HRMS. Results on the micro-extraction work and the structure elucidation of all weld flavones are reported below.

## 2. Results

### 2.1. UHPLC Separation

**Figure 1:**
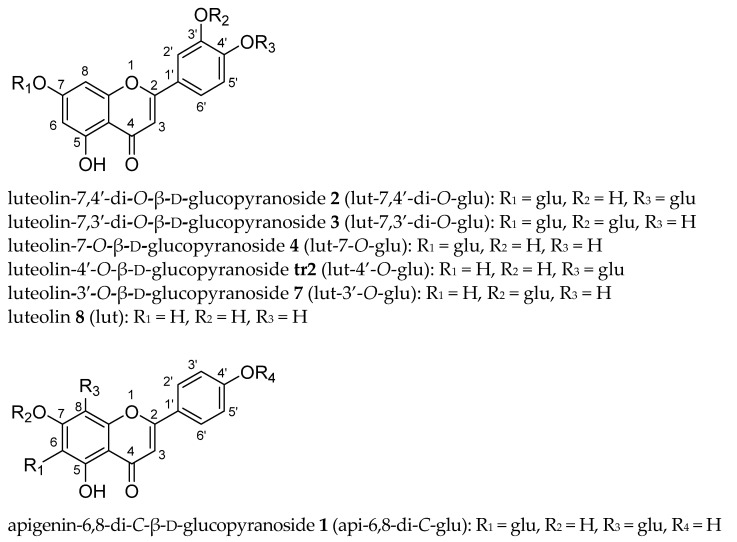

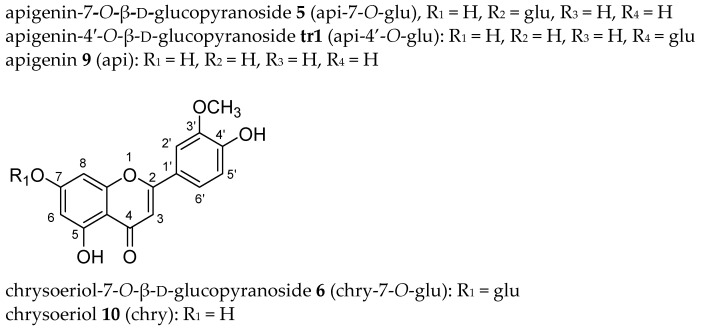
Structures of all flavones identified in weld (*R. luteola*). Numbers in bold, refer to peak numbers in Figure 2; **tr1** & **tr2** = two trace flavones not visible in Figure 2; glu = β-d-glucopyranoside.

**Figure 2:**
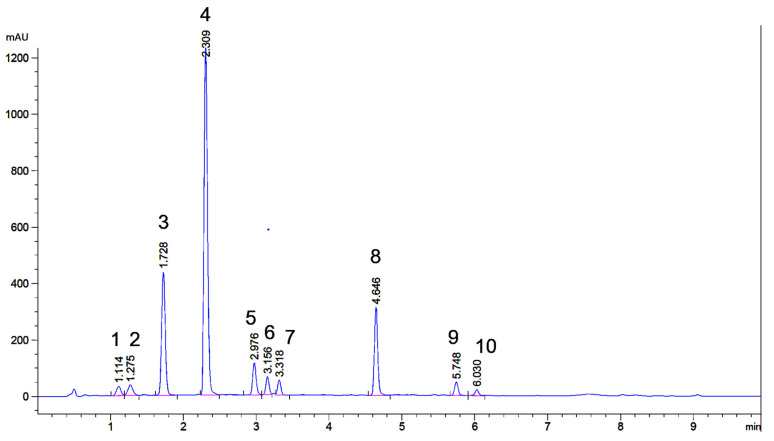
Reversed phase UHPLC profile of an extract of weld; UV detection at 345 nm. Peaks of the ten main flavones are numbered and correspond with the bold numbers in Figure 1.

As a first step, a UHPLC method for obtaining a fast chromatographically well-resolved fingerprint chromatogram of weld was developed. Starting point was the chromatogram obtained earlier with a 50 mm × 3.0 mm UHPLC column in combination with a standard HPLC pump [37]. To obtain more resolution of the three minor flavones eluting in between lut-7-*O*-glu and lut (see Figure 1 for structures), a 100 × 2.1 mm 1.8 μm UHPLC column was used instead. To obtain less band broadening, higher reproducibility and higher sensitivity, the column was mounted in a modern UHPLC set-up. The A (40 mM formic acid buffer pH = 3 with 0.04 mM EDTA) and B (acetonitrile) solvents remained the same as before [37] but gradient and flow were adapted because of the increased length and decreased diameter of the new column. With a gradient from 15% to 45% of acetonitrile in 8 min, the chromatogram shown in Figure 2 was obtained for the weld extract showing baseline resolution of the ten peaks with ≥1% relative abundance (based on normalized peak areas at UV 350 nm). Weld extracts appear to be remarkably constant in their composition as this chromatogram shows much correspondence with LC-UV weld chromatograms published by other groups [3,23,25,26,28]. The profiles published by Moiteiro et al. and Gaspar et al. somewhat deviate, but this could be due to a different selectivity of the used C18 stationary phase [34,36]. A simple quadrupole mass spectrometer operating in negative mode was connected in series and provided useful initial data about the MW of the different peaks, especially in extracted ion chromatography (EIC) mode.

### 2.2. Microextraction Method for Weld-Dyed Wool

As a next step, the extraction procedure (300 μL of solvent in a 2 mL vial) used earlier by Villela et al. for weld-dyed wool was downscaled to enable the analysis of 50 μg of sample [30]. Initially, we attempted to use a procedure used for the extraction of ng amounts of sex pheromone [38]. Said procedure used 5 μL of solvent in a shortened melting point tube to extract an SPME needle. Although successful analyses of weld-dyed wool were obtained, in practice it proved extremely difficult to transfer <100 μg of wool threads into a melting point tube. Additionally, due to the small volume available for injection, the standard UHPLC autosampler could not be used and hardware adaptations to enable manual injection were required. For the same reason, filtration posed a problem. Because of these limitations, this approach was abandoned and 250 μL commercially available inserts for autosampler vials were tried instead.

**Figure 3:**
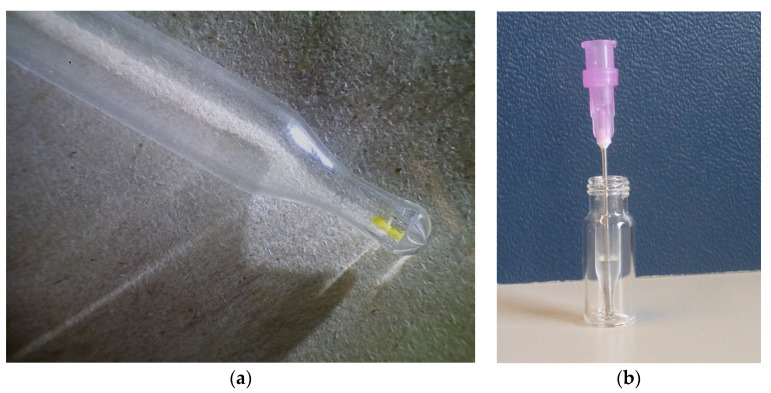
(**a**) bottom part of 250 μL insert for 1.8 mL HPLC autosampler vial with <0.1 mg weld-dyed wool sample at the very bottom; (**b**) autosampler vial with insert containing ~100 μL of extracted wool sample after filtration through a zero dead volume 5 μm syringe filter. The purple needle filter can still be seen.

A minute amount of yellow lab-dyed wool was taken by means of chirurgic scissors and transferred into an empty insert previously weighed on a μg balance (readable up to 0.1 μg) (Figure 3a). After weighing again, 0.5 μL of extraction solvent (methanol-water-formic acid (80:15:5, *v*/*v*/*v*) was added per μg of wool. After a 30 min extraction step at 60 °C, the extract was diluted five times with water and filtered through a 5-μm zero-dead volume needle filter into a clean empty insert (Figure 3b). This dilution step enabled the filtration step. A filtration step is advisable to avoid clogging of the UHPLC column with wool fibres. The dilution step did not affect the sensitivity as the higher water content of the sample allowed a five times bigger injection volume. Ten μL were injected into the UHPLC by means of the standard autosampler.

**Figure 4:**
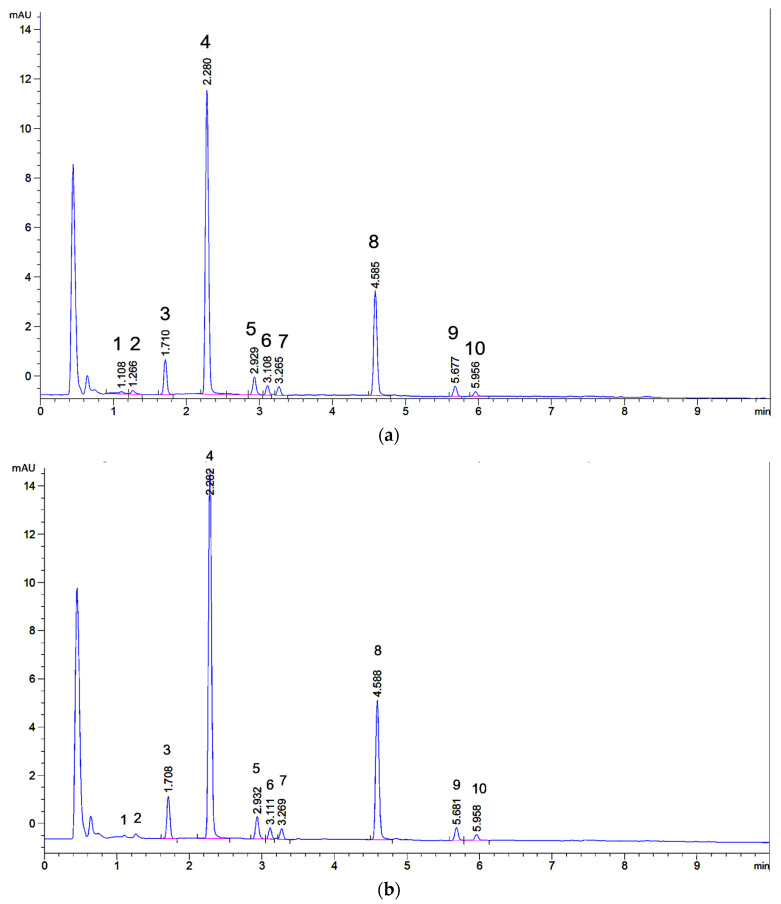

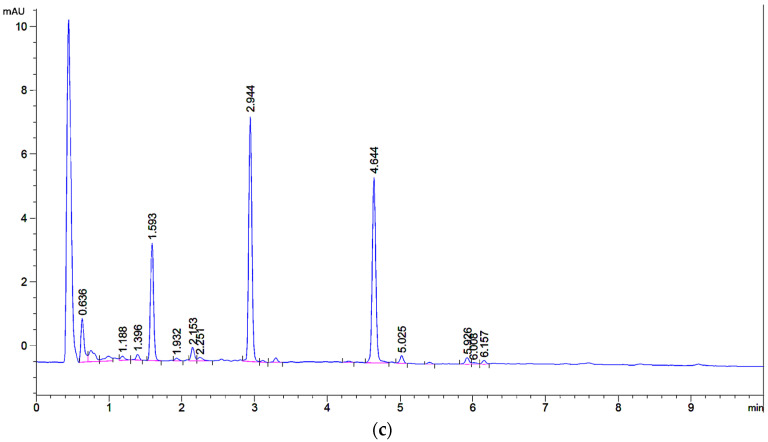
UHPLC profiles of extracted wool samples (**a**). extract of 56.6 μg weld-dyed wool. (**b**). extract of 49.2 μg weld-dyed wool. Peak numbers refer to Figure 1. (**c**). extract of 28.3 μg of onion-dyed wool.

In Figure 4a,b, the chromatograms of 56.6 μg and 49.2 μg of weld-dyed wool respectively are depicted. The fingerprints are qualitatively identical to the one of weld extract in Figure 2. Quantitatively the flavone diglucosides (peaks **1**, **2,** and **3**) are less prominent in the extracted wool sample as compared to the original weld extract (Figure 2). This can be due to either the dyeing or the extraction. Willemen et al. noted that flavone diglucosides are less well bound during the dyeing process than the monoglucosides and aglycones [5]. The total sample volumes (~125 μL) allowed for duplicate injections into the UHPLC. As expected, intraday retention times were constant within 0.003 min and variation of relative peak areas was ≤1% (Supplementary Figures S68 and S69). In both chromatograms, the 10 peaks constituting the fingerprint are clearly resolved and well above background noise. Although peak **1** appears small, it can still be integrated. To determine whether the proposed technique is capable of differentiating wool samples dyed with another natural yellow dye, onion-dyed wool was extracted in duplicate. This also served to see if even smaller samples can be processed and successfully analysed. In Figure 4c, a chromatogram of 28.3 μg onion-dyed wool is shown. From the UHPLC profile, it can be concluded that the microextraction methodology also works for other yellow flavonoid-based dyes and that wool quantities <50 μg can be processed. Onion contains flavonoids of the flavonol type, with quercetin-3,4′-di-*O*-glucoside, quercetin-4′-*O*-glucoside, and quercetin as major compounds [39].

### 2.3. Preparative Isolation of Major and Minor Flavones of Weld

As the literature on the identity of weld flavones was not 100% clear, next it was decided to isolate at least a few mg of each flavone corresponding with peaks **1**–**10** in Figure 2 and Figure 4a. This should suffice to record 1-dim and 2-dim NMR spectra as well as high-resolution mass spectra (HRMS). This was carried out by extracting 200 g of weld followed by repeated column chromatography on polyamide and preparative RP-HPLC. In addition to the successful isolation of the ten flavones corresponding to peaks **1**–**10**, two additional trace flavones (<1% normalised peak area), named **tr1** & **tr2** (Figure 1) were isolated. So, in total, 12 flavones were isolated from weld in pure form, five of which elute between 2.8 and 3.4 min. Due to their low concentration and overlap, the two trace flavones are not visible in the UHPLC fingerprint, but their NMR spectra were recorded nonetheless.

### 2.4. Identification of Isolated Flavones by NMR and HRMS

The 12 compounds isolated were expected to be flavones based on the extraction method, retention times and especially their distinctive on-line UV spectra (see for example Figures S73–S75). Their 1-dimensional and 2-dimensional 600 or 700 MHz NMR spectra were recorded in DMSO-*d*_6_ and fully assigned. All spectra can be found in the supplementary materials (Supplementary Figures S1–S67). In Table 1, ^1^H NMR shifts and coupling constants are presented together with ^13^C NMR shifts. Particular attention was given to the point of attachment of sugar units and the configuration of the sugar units. When available, retention times were compared with those of authentic standards. In all cases, high-resolution mass spectra (HRMS) were also recorded to confirm the NMR assignments. In the following three paragraphs, starting with aglycones and ending with the diglucosides, results are discussed and also compared with published NMR spectra.

#### 2.4.1. Flavone Aglycones

The literature indicates that the three aglycones present in weld are luteolin, apigenin and chrysoeriol [40]. Based on their lower polarity and correspondence with a published fingerprint [40], the three last eluting peaks were thus expected to be luteolin (lut, peak **8** in Figure 2)—the main aglycone of weld—apigenin (api, **9**) and chrysoeriol (chry, **10**). The structures of all flavones are depicted in Figure 1. For lut and api, the peak assignment could be confirmed by authentic references. Their identity was further fully confirmed by comparison with ^1^H and ^13^C NMR data published in literature [41,42,43,44] and full interpretation of their HMBC spectra (see Supplementary Figures S3, S7 and S12). The NMR data of chry were in good agreement with the literature except for the assignment of H-6 and H-8 by Kim [44,45]. Assignment based solely on 1-dim NMR data are less reliable than those based on both 1-dim and 2-dim NMR data as in this study. This has been shown for other flavones too [42]. The position of the methyl group in chry was unequivocally assigned to be 3′ based on its ROESY spectrum (Supplementary Figure S13). The HRMS data (Supplementary Table S1) further substantiated the NMR data and assignments. The NMR data of the three aglycones were useful for the interpretation of the NMR spectra of the monoglucosides and diglucosides discussed in the next two paragraphs.

#### 2.4.2. Flavone Monoglucosides

Based on the literature, the following flavone monoglucosides were expected: lut-7-*O*-glucoside, lut-3′-*O*-glucoside, lut-4′-*O*-glucoside, api-7-*O*-glucoside and a chry-*O*-glucoside with the main component being lut-7-*O*-glu (peak **4** in Figure 2). Again, all NMR data can be found in the Supplementary Materials. Based on their intermediate polarity, the monoglucosides (peaks **4**–**7**, Figure 2) elute in between the diglucosides (peaks **1**–**3**) and aglycones (peaks **8**–**10**). Their identification is discussed below, peak by peak in order of increasing retention times.

The major compound (peak **4**) should be lut-7-*O*-glucoside. This was confirmed by comparison of its retention time with a reference and HRMS. Additionally, there was a good correspondence with published NMR data except for the assignments of H-3, H-6 and H-8 by Chung, which seem erroneous [41,43,46,47]. This confirmed the hexose to be glucose. The HMBC spectrum further substantiated the attachment of the glucose unit to O-7. As *J*_1″2″_ is 7.6 Hz, lut-7-*O*-glu isolated from weld is lut-7-*O*-β-d-glucopyranoside **4**. This exactly matches an earlier assignment [48].

The HRMS and ^1^H NMR spectrum of the flavone corresponding with peak **5** suggested the presence of both apigenin and a hexose unit. The downfield shift of H-6 and H-8 indicated that the sugar unit was probably attached to O-7. The C-7/H-1″ cross-peak in the HMBC spectrum confirmed this. Its ^13^C NMR spectrum was virtually identical to the one reported by Oyama and Kondo for api-7-*O*-β-glu [49]. This, and a *J*_1″2″_ of 7.7 Hz, proved that peak **5** corresponded with api-7-*O*-β-d-glucopyranoside **5**.

The HRMS and ^1^H NMR of the flavone corresponding with peak **6** pointed in the direction of chrysoeriol as aglycone and a hexose. Again, the downfield shift of H-6 and H-8 made it clear that the sugar was attached to O-7. This was confirmed by a C-7/H-1″ cross-peak in the HMBC spectrum. The location of the methoxy group at C-3′ as well as that of the sugar moiety to C-7 were confirmed based on the ROESY spectrum. The ^13^C NMR spectrum gave a good match with the one reported by Harput et al. [47] for chry-7-*O*-glucopyranoside. This, and a *J*_1″2″_ of 7.6 Hz, confirmed the main compound of the sample to be chry-7-*O*-d-glucopyranoside **6**.

The HRMS as well as the ^1^H NMR data of the trace flavone **tr1** were indicative of an apigenin-hexose. Relative to the ^1^H NMR of apigenin itself, H-3′ and H-5′ had shifted downfield suggesting the hexose to be attached to O-4′. The C-4′ and H-1″ cross-peak in the HMBC confirmed this. The spectra matched those reported by Oyama and Kondo [49] and Ding et al. [50] for api-4′-*O*-β-d-glucopyranoside well. This, and a *J*_1″2″_ of 7.4 Hz, led to the conclusion that this minor compound is api-4′-*O*-β-d-glucopyranoside **tr1**. This compound has not yet been reported to occur in *R. luteola*.

The mass of the second trace flavone **tr2** was that of a luteolin-hexose. Based on the identification of lut-7-glu (peak **4**) and lut-3′-glu (peak **7**, vide infra) in combination with the identification of api-4′-glu, lut-4′-*O*-glu appeared a plausible candidate. The 0.3 ppm downfield shift of H-5′ supported this hypothesis. Furthermore, C-4′ can be discriminated from C-3′ by long range couplings in the HMBC of C-4′ to both H-2′and H-6′. The anomeric proton couples with C-4′ and therefore this compound was confirmed as lut-4′-*O*-glu. A good fit with the ^13^C spectrum published by Lee et al. [51] for lut-4′-*O*-β-d-glucopyranoside and a *J*_1″2″_ of 7.3 Hz, led to the identification of this second minor compound as lut-4′-*O*-β-d-glucopyranoside **tr2**.

The HRMS indicated that peak **7** corresponded with a luteolin-hexose. In the ^1^H NMR H-3, 6 and 8 had the same chemical shifts as in luteolin **8** itself but H-2′ and 6′ had shifted suggesting attachment of the sugar to O-3′. This was proven by a C-3′ and H-1″ cross-peak in the HMBC spectrum. The ^1^H data matched published data [43]. The ^13^C data of lut-3′-*O*-glu differed less than 1 ppm (sugar moiety C-atoms) and less than 3 ppm (other C-atoms) from those reported by Markham et al. [41]. This confirmed the identity of the main compound of the sample, including the nature of the hexose. As L-glucose does not occur in nature, the sugar moiety of the flavonoid is d-glucose. Furthermore, as the magnitude of the coupling constant H-1″/H-2″ is in the range 7–8 Hz, the configuration of the anomeric C-atom could be ascertained as β, and the cyclic form of the glucose as pyranosidic [43]. Thus, lut-3′-*O*-glu isolated from weld is lut-3′-*O*-β-d-glucopyranoside **7**. Whereas this configuration of the anomeric C-atom is the same as that of an earlier assignment [48], the glucose is in the pyranose form, and not in the furanose form as claimed by Batirov et al. [48].

In the past, also HPLC has been used by us to separate the weld flavones [37]. With a run time of one hour, lut-4′-*O*-glu **tr2** can be observed as a minor peak eluting just after chry-7-*O*-glu **6** while api-4′-*O*-glu **tr1** co-elutes with both with chry-7-*O*-glu **6** and lut-4′-*O*-glu **tr2** (Supplementary Figure S70). According to the Extracted Ion Chromatograms (EICs), the UHPLC system has a slightly different selectivity, which could be due to a different C18 phase, different gradient, or the use of acetonitrile instead of methanol. In the UHPLC system the trace flavones api-4′-*O*-glu **tr1** and lut-4′-*O*-glu **tr2** partially coelute with api-7-*O*-glu **5**. For EICs see Figures S76–S83.

#### 2.4.3. Flavone Diglucosides

Based on the literature, the following diglucosides were expected: lut-7,3′-di-*O*-glucoside **3**, an isomer thereof (peak **2**), and api-6,8-di-*C*-glucoside (peak **1**). The identification is discussed in order of decreasing retention times (Figure 2).

The mass of the main diglucoside (peak **3**) was in agreement with lut-7,3′-di-*O*-glucoside, one of the main flavones of weld. In the ^1^H NMR spectrum H-2′, H6′ and H-5′ had near identical shifts as the same protons in lut-3′-*O*-glu **7** while the shifts of H-3, H-6, H-8 corresponded well with those of the same protons in lut-7-*O*-glucoside **4**. The attachment of hexoses to O-7 and O-3′ was further confirmed by cross-peaks in the HMBC between C-7 and H-1″, and C-3′ and H-1‴. The ^13^C NMR was in excellent agreement with the one reported by Markham et al. [41] for lut-7,3′-di-*O*-glu. This confirmed both hexoses to be glucose. As *J*_1″2″_ is 7.2 Hz and *J*_1‴2‴_ is 7.1 Hz, the compound isolated from weld is lut-7,3′-di-*O*-β-d-glucopyranoside **3**. The spectral identification was confirmed by chromatographic evidence: an authentic reference had the same retention time.

The mass of the compound corresponding with peak **2** was the same as that of the major diglucoside lut-7,3′-di-*O*-glu (peak **3**). In the ^1^H NMR spectrum H-2′, H6′ and H-5′ had near identical shifts as the same protons in lut-4′-*O*-glu while the shifts of H-3, H-6, H-8 corresponded well with those of the same protons in lut-7-*O*-glucoside **4**. This pointed in the direction of lut-7,4′-di-*O*-glu. Cross-peaks in the HMBC spectrum between C-7 and H-1″, and C-4′ and H-1‴ proved that the sugar moieties were indeed attached to O-7, and O-4′. The NMR data of the compound are consistent with the aglycone being lut. As there are no ^1^H- or ^13^C-NMR data reported for lut-7,4′-di-*O*-glu in the literature, the ^13^C shifts of its sugar moieties were compared with those obtained for lut-7-*O*-glu and lut-4′-*O*-glu (Table 1). The differences between them were <1.0 ppm, confirming both hexoses to be glucose. Similarly, there were excellent matches with the shifts of C-2 to C-8a of lut-7-*O*-glu **4** and of C-1’ to C-6′ of lut-4′-*O*-glu **tr2** (Table 1). As *J*_1″2″_ is 7.7 Hz and *J*_1‴2‴_ is 7.1 Hz, the compound is lut-7,4′-di-*O*-β-d-glucopyranoside **2**.

The HRMS corresponding with peak **1** gave as elemental composition C_27_H_30_O_15_ (Supplementary Table S1), which is the composition of the expected api-6,8-di-*C*-glucoside **1**. The absence of H-6 and H-8 signals in the ^1^H and HSQC spectra suggested that the sugar moieties were indeed attached to C-6 and C-8. This was supported by cross-peaks in the HMBC spectrum between C-5 and H-1″, C-6 and H-1″, C-8a and H-1‴, and C-8 and H-1‴. The remaining ^1^H NMR data were consistent with the aglycone being api and the presence of two hexoses. The ^13^C shifts of the anomeric carbons further supported the assignment as their signals (~75 ppm) appeared more upfield than those of the *O*-glycosides (~100 ppm), see Table 1. Additionally, there was an excellent fit with the ^13^C NMR spectrum reported by Sato et al. [52] for api-6,8-di-*C*-β-d-glucopyranoside in DMSO-*d*_6_ at 90 °C. The H-1″ and H-1‴ shifts of 4.9 ppm, and the *J*_1″2″_ and *J*_1‴2‴_ of 10 Hz were consistent with 6- and 8-*C*-β-d-glucosyl moieties [43]. The combined data show this flavone to be api-6,8-di-*C*-β-d-glucopyranoside **1** (synonym: vicenin 2).

## 3. Discussion

Starting from only 50 µg of weld-dyed wool and using a simple extraction procedure, high quality chromatographic fingerprints consisting of ten peaks can be obtained (Figure 4a,b), whereby “high quality” is defined in terms of overall baseline stability, chromatographic resolution, peak shape and signal-to-noise ratio. Due to the mild extraction conditions, the flavone aglycones and glucosides originally present in a weld extract are all detectable in the wool extract. This makes the fingerprint representative for weld. For instance, distinction from another yellow dye proved easy (Figure 4c). After membrane filtration to remove extracted wool threads, the final volume available for injection suffices for duplicate injections. As the injections are highly reproducible, one could also resort to single injections. This could in principle increase the sensitivity further, either by injecting a larger volume or by decreasing the initial extraction volume. However, the current sensitivity is sufficient as exemplified by an estimated signal-to-noise ratio of at least 100 for a 1% component like chrysoeriol **10**. Actually, the limiting factor appears to be chemical noise and baseline stability rather than electronic noise. Telling UV spectra could be obtained as evidenced by the those of luteolin **8**, apigenin **9** and even chrysoeriol **10**, which has a peak height < 0.25 mAU (Supplementary Figures S73–S75). Starting with smaller quantities of wool would be possible too, but amounts smaller than 25 µg are in practice difficult to handle. If needed, another possibility for increasing the sensitivity would be to mount a 60 mm flow cell, which has a five times lower LOD [33]. The current surplus sensitivity could facilitate the investigation of historic textiles, which possibly contain lower concentrations of dye components due to decomposition. The method might also be used for following artificial ageing of dyed textiles as relative changes in flavone concentrations can be easily monitored even if decomposition products do not absorb at 345 nm.

The separation was carried out by UHPLC with diode-array detection (DAD). This allowed for a fast (6 min), efficient (baseline separation of all 10 flavones), selective and sensitive detection. Mass spectrometric detection in series with the DAD was attempted, but for this particular analysis, the simple quadrupole MS was less sensitive than a modern DAD. ESI-MS in negative mode could be used for the original weld extract but not for extracts of 50 µg of dyed wool as these contain 100 times less material. A more expensive MS will be able to provide the required sensitivity. Although on-line MS was still useful for peak assignments, for dye analysis mass spectrometry is perhaps slightly less selective than UV-Vis, as it may detect also non-dye compounds and is poorly suitable for distinguishing between different isomers such luteolin 3′- and 4′-glucosides. Thus, when only MS [27] is used for dye analyses instead of the combination DAD-MS, important information is lost.

To distinguish between isomers, NMR is a much more powerful method. A disadvantage of NMR is that it requires many preliminary purification steps. In this study 1-dim and 2-dim NMR successfully allowed the unequivocal identification of the ten flavones corresponding with the ten fingerprint peaks. Additionally, two trace flavones were identified. All of the twelve flavones had been reported for weld earlier with the exception of apigenin-4′-*O*-β-d-glucopyranoside **tr1**. The analysis of weld by Marques et al. is correct [26] although they did not detect either lut-3′-*O*-glu or lut-4′-*O*-glu. The correction by Gaspar et al. [36] of their earlier assignment [34] of these two compounds is justified, indeed the 3′-isomer elutes later than 4′-isomer. In the chromatographic system used by Gaspar et al., these two isomers are much better separated than in our system. Thus, some controversy with regard to the elution order of the different flavones was solved and this may assist future analyses of weld-dyed textiles. Future work should focus on expansion of the micro-extraction and UHPLC approach to other dye plants like madder.

## 4. Materials and Methods

### 4.1. Chemicals and Materials

Luteolin-7-monoglucoside and luteolin-7,3′-diglucoside (analytical control grade) were from Extrasynthèse. Luteolin was from Extrasynthèse or Sigma. Ammonium formate was from Aldrich or Fluka and EDTA tetrasodium salt dihydrate was from Aldrich. Aluminum chloride and formic acid were from Acros. The purity of formic acid used in extractions was 98%+ and for wool treatment 99%. Methanol (LC-MS grade) was from Prolabo and ultrapure water was prepared with an EasyPure system of Barnstead/Thermoline. Other chemicals were of analytical grade. Wool was weighed on a Mettler UM3 μg balance. *Reseda luteola* L. plants were grown in the province of Groningen, The Netherlands.

### 4.2. Wool Dyeing

Wool was dyed by students with weld as described by Villela et al. [30]. Five by five cm pieces of Kova medium weight wool sateen white (with a sateen finish on one side; natural cream colour; fabric is ready for dyeing and/or printing; approximately 290 g/m^2^; from Whaleys, Bradford) were pre-mordanted with aluminum potassium sulphate dodecahydrate (alum). Reseda extract was prepared by sonicating 1.5 g of dried and ground weld with 30 mL of 96% alcohol-water 3:1 (*v*/*v*) for 10 min in an ultrasonic bath for the filtration and removal of alcohol. The resulting extract was diluted with 60 mL of water, heated to 80 °C, after which 4 pieces of pre-soaked, pre-mordanted wool were added and stirred during 15 min. Afterwards the pieces were rinsed with hot and cold water and dried. Wool was dyed in 2011 and stored in the dark at room temperature until extracted in 2019. Wool threads were removed just prior to the analysis. Onion-dyed wool: alum pre-mordanted wool was dyed during educational activities with the outer (paper-like) scales of onions in the same way as weld with the exception of starting from dry onion extract. The onion extract–wool ratio was approximately 1:100. Photos of both weld-dyed and onion-dyed wool can be found in Supplementary Figures S71 and S72 respectively.

### 4.3. Wool Micro-Extraction

Approximately 50 μg of weld-dyed wool was taken by means of chirurgic scissors and transferred into an empty 250 µL insert for a standard 1.8 mL autosampler vial previously weighed on a μg balance (readable up to 0.1 μg) (Figure 3a). After weighing again to determine the amount of wool taken, 0.5 μL of extraction solvent (methanol-water-formic acid (80:15:5, *v*/*v*/*v*) was added per μg of wool. After firmly closing the cap with an inert seal to avoid any evaporation of solvent during the extraction, a 30 min extraction step at 60 °C followed by placing the vial in a water bath. Next, the extract was diluted five times with ultrapure water and filtered through a 5 μm zero-dead volume needle filter (Sol-M blunt needle fill needle, W/5 micron filter, 110022F, Sol-Millennium) into a clean and empty 250 µL insert (Figure 3B). This dilution step enabled the filtration step. A filtration step is advisable to avoid clogging of the UHPLC column. Ten μL were injected into the UHPLC by means of the standard autosampler. The photo of dyed wool in the insert (Figure 3a) was taken by means of a digital microscope (DNT, The Netherlands).

### 4.4. Preparative Isolation and Purification of Weld Flavones

Aerial parts of dried *Reseda luteola* L. (cultivar code B) plants were used. The plants were grown, harvested and dried as described by Villela [37]. This plant material was ground with a cutting mill (Retsch GmbH, type SM1, Haan/Germany) equipped with a 0.5 mm sieve.

Compounds of low polarity were removed from 200 g of the ground plant material by stirring overnight with 2 L of petroleum ether 40°/60°-MTBE (1:1). After removal of solvents, the compounds of interest were extracted from the defatted plant material by stirring overnight with 2 L of 96% alcohol-water (8:2). The next morning, the solvent was heated till near boiling after which the plant material was removed by vacuum filtration through a glass filter (por 40). The plant material was extracted anew with 0.5 L of fresh alcohol-water (8:2) and filtered through the same glass filter. The two extracts were combined after which all solvent was removed with a rotary evaporator. The hydroalcoholic extract was fractionated via repeated polyamide column chromatography using different water–acetone gradients as eluent. Final separation and purification took place by preparative RP-HPLC (Shimadzu prepHPLC, Altima C18 column, 250 × 22 mm, 5 μm, A eluent: 2.1 L of water with 12.9 mL of formic acid and 5.05 g of ammonium bicarbonate, B eluent: methanol, various gradients were used, DAD monitoring wavelength 350 nm). Eluents were removed by rotary evaporation and freeze drying. The progress of the purification was monitored by TLC, RP-HPLC–UV, and ^1^H-NMR.

### 4.5. UHPLC

An Agilent 1290 Infinity system equipped with binary pumps (G4220A), autosampler (G4226A), thermostatted (25 °C) column compartment (G1316C) and DAD (G4212A) in combination with a 60008 10 mm flow cell was used. The column was an Agilent Zorbax Eclipse Plus C18, 2.1 × 100 mm 1.8 µm). Mobile phase A: 40 mM formic acid buffer pH = 3 in water with 0.04 mM EDTA; mobile phase B: acetonitrile. Flow rate: 0.45 mL/min, initial pressure 511 bar. Linear gradient: 0 min 15% B; 8 min 45% B; Monitoring wavelength: 345 nm. Injection volume: 10 µL. For some analysis of the extract, the effluent of the DAD was connected to an Agilent Mass Spectrometer Detector (G6125B). The MSD was operated in negative mode with a *m/z* 150–800 scan range. Data were viewed as TIC and EIC plots.

### 4.6. NMR Spectroscopy

All 1-dim and 2-dim NMR spectra were recorded either on a Bruker Avance III 600 MHz NMR spectrometer or a Bruker Avance III 700 MHz NMR. Both were equipped with a 5 mm cryoprobe. Spectra were recorded in either 5 or 3 mm NMR tubes. The temperature of the sample was kept at 300 K. HSQC and HMBC spectra were recorded with standard pulse sequences. ROESY spectra were recorded on a Bruker Avance III 700 MHz NMR spectrometer with a standard pulse sequence and 225 µs mixing time. The ^1^H spectra of api-7-*O*-glu **5**, api-4′-*O*-glu **tr1** and chry-7-*O*-glu **6** were acquired with the standard Bruker pulse sequence zgcppr for suppression of the water signal. ^1^H spectra of api-6,8-di-*C*-glu **1** were recorded in DMSO-*d*_6_ in a 3 mm probe at 300 and 320 K prior to dilution of the sample with 2 volumes MeOH-*d*_4_ and transfer of the solution to a 5 mm tube; the ^1^H spectrum and 2D spectra of api-6,8-di-*C*-glu **1** in DMSO-*d*_6_—MeOH-d4 (1:2) were recorded at 300 K.

Between 0.7 and 2.7 mg of each of the 12 flavones was dissolved in 0.6 mL (5 mm probe) or 0.2 mL (3 mm probe) of DMSO-*d*_6_. Data interpretation: C–H pairs were assigned in the HSQC spectrum and assignments were confirmed using the HMBC spectrum. ^13^C δ of methine C-atoms reported in Table 1 were obtained from the HSQC experiment, and those of the quaternary C-atoms from the HMBC experiment. Chemical shifts were referenced against the peak of DMSO (δ (^1^H) = 2.55 ppm and δ (^13^C) = 40.45 ppm).

### 4.7. High-Resolution Mass Spectrometry

The twelve fractions containing the purified flavones were analysed by high-resolution mass spectrometry. The solutions were directly infused at a flow rate of 3 μL/min via a syringe pump into a Thermo Fisher Exactive Orbitrap mass spectrometer. The analyses were carried by electrospray, in negative ionisation mode. Settings were: 250–2000 *m/z* (scan range), ultrahigh resolution, 3.50 kV (spray voltage), 400 °C (capillary temperature), −25.00 V (capillary voltage). Measurements were carried out continuously during 0.6–1.7 min, and the data displayed in Supplementary Table S1 were obtained from 31–70 scans averaged spectra.

## Figures and Tables

**Table 1:** ^1^H-NMR and ^13^C-NMR data of weld flavones; numbering of compounds as in Figure 1 (DMSO-*d*_6_, 300 K).

	**lut (8)**		**lut-7-*O*-glu (4)**		**lut-3′-*O*-glu (7)**		**lut-4′-*O*-glu (tr2)**		**lut-7,3′-di-*O*-glu (3) ^a^**		**lut-7,4′-di-*O*-glu (2) ^a^**	
**Position**	**^1^H δ (ppm), Mult. ^b^; *J* (Hz)**	**^13^C δ (ppm)**	**^1^H δ (ppm), Mult.; *J* (Hz)**	**^13^C δ (ppm)**	**^1^H δ (ppm), Mult.; *J* (Hz)**	**^13^C δ (ppm)**	**^1^H δ (ppm), Mult.; *J* (Hz)**	**^13^C δ (ppm)**	**^1^H δ (ppm), Mult.; *J* (Hz)**	**^13^C δ (ppm)**	**^1^H δ (ppm), Mult.; *J* (Hz)**	**^13^C δ (ppm)**
2	-	164.9	-	165.3	-	164.4	-	162.8	-	164.8	-	164.7
3	6.70	103.2	6.80	103.9	6.78	103.2	6.64	104.0	6.95	104.0	6.94	104.8
4	-	182.5	-	182.6	-	182.4	-	n.d.	-	182.9	-	183.0 **
5	-	162.3	-	162.0	-	162.3	-	n.d.	-	161.7	-	162.0
4a	-	104.4	-	106.1	-	104.3	-	101.9	-	106.3	-	106.3
6	6.22, d; 1.2	99.6	6.49, d; 1.9	100.2	6.20, d; 1.5	99.8	5.92	101.4	6.48	100.2	6.49	100.4
7	-	165.5	-	163.7	-	166.2	-	n.d.	-	163.9	-	164.0
8	6.48, d; 1.2	94.6	6.84, d; 1.9	95.4	6.55, d; 1.5	94.9	6.22	95.8	6.94	95.3	6.91	95.5
8a	-	158.4	-	157.7	-	158.4	-	n.d.	-	158.0	-	157.9
1′	-	122.1	-	122.1	-	121.1	-	126.2	-	123.2	-	125.2
2′	7.44, d; 2.1	113.9	7.47, d; 2.2	114.2	7.80, d; 1.7	115.5	7.46	114.4	7.90	115.3	7.60, d; 2.0	114.7
3′	-	146.8	-	146.6	-	147.0	-	148.7	-	146.7	-	148.4
4′	-	150.9	-	150.9	-	153.5	-	149.5	-	151.9	-	149.9
5′	6.92, d; 8.3	116.6	6.95, d; 8.4	116.7	6.96, d; 8.5	117.6	7.24, d; 8.4	116.7	7.02, d; 8.3	117.1	7.28, d; 8.6	116.5
6′	7.46, dd; 8.3, 2.1	119.6	7.50, dd; 8.4, 2.2	119.9	7.66, dd; 1.7, 8.5	122.8	7.43, d; 8.4	118.0	7.73, d; 8.3	122.7	7.57, dd; 8.6, 2.0	118.9
												
1″			5.13, d; 7.6	100.6	4.88, d; 7.1	103.1	4.87, d; 7.3	102.2	5.11, d; 7.2	100.4	5.12, d; 7.7	100.5
2″			3.31, pt; 7.6, 8.9	73.8	3.36, n.r.	74.0	3.36, n.r.	74.0	3.32, n.r.	73.7	3.37, n.r.	73.9
3″			3.35, pt; 8.9, 8.9	77.1	3.36, n.r.	76.8	3.35, n.r.	76.6	3.37, n.r.	76.7	3.37, n.r.	76.5
4″			3.23, pt; 8.9, 8.9	70.2	3.21, pt; 8.8, 8.8	70.6	3.22, pt; 8.7, 8.7	70.4	3.26, n.r.	70.1	3.23, n.r.	70.4
5″			3.50, n.r.	77.9	3.48, m	78.1	3.42, n.r.	77.9	3.49, n.r.	77.7	3.51, n.r.	77.8
6″			3.76/3.53, d; 11.6/n.r.	61.3	3.83/3.56, d; 11.5/dd; 11.5, 6.4	61.7	3.77/3.53, d; 11.2/dd; 11.2, 5.7	61.4	3.75/3.56, d; 9.4 */d; 9.4 *	61.1	3.76/3.53, n.r./n.r.	61.3
		
1‴									4.92, d; 7.1	102.6	4.90, d; 7.1	102.1
2‴									3.39, n.r.	74.1	3.31, n.r.	73.8
3‴									3.34, n.r.	77.1	3.36, n.r.	77.1
4‴									3.18, n.r.	70.9	3.23, n.r.	70.4
5‴									3.54, n.r.	78.1	3.43, n.r.	78.0
6‴									3.83/3.53, d 11.6 */d; 11.6 *	61.8	3.76/3.53, n.r./n.r.	61.3
	**api (9)**		**api-7-*O*-glu (5)**		**api-4′-*O*-glu (tr1)**		**api-6,8-di-*C*-glu (1) ^c^**		**chry (10)**		**chry-7-*O*-glu (6)**	
**Position**	**^1^H δ (ppm), Mult.; *J* (Hz)**	**^13^C δ (ppm)**	**^1^H δ (ppm), Mult.; *J* (Hz)**	**^13^C δ (ppm)**	**^1^H δ (ppm), Mult.; *J* (Hz)**	**^13^C δ (ppm)**	**^1^H δ (ppm), Mult.; *J* (Hz)**	**^13^C δ (ppm)**	**^1^H δ (ppm), Mult.; *J* (Hz)**	**^13^C δ (ppm)**	**^1^H δ (ppm), Mult.; *J* (Hz)**	**^13^C δ (ppm)**
2	-	164.3	-	165.4	-	162.6	-	166.9	-	163.8	-	n.d.
3	6.75	103.1	6.84	102.7	6.74	104.0	6.64	104.4	6.80	103.3	6.75	101.0
4	-	182.2	-	182.6	-	n.d.	-	n.d.	-	n.d.	-	n.d.
5	-	162.2	-	161.8	-	n.d.	-	163.4	-	n.d.	-	161.9
4a	-	103.7	-	106.2	-	102.1	-	106.2	-	n.d.	-	105.9
6	6.15	100.1	6.47, d; 1.4	100.1	5.94	101.2	-	108.9	6.06	100.3	6.43	99.7
7	-	167.2	-	163.8	-	n.d.	-	n.d.	-	n.d.	-	163.3
8	6.43	94.9	6.86, d; 1.4	95.3	6.22	95.6	-	106.9	6.35	95.1	6.83	95.1
8a	-	158.3	-	157.7	-	n.d.	-	157.9	-	n.d.	-	157.5
1′	-	121.7	-	117.5	-	125.4	-	124.0	-	121.9	-	n.d.
2′	7.93, d; 8.8	128.8	7.95, d; 8.4	129.2	8.01, d; 8.6	128.3	7.97, d; 8.5	130.6	7.54	110.4	7.42	110.0
3′	6.96, d; 8.8	116.7	6.89, d; 8.4	117.2	7.21, d; 8.6	117.1	6.91, d; 8.5	117.4	-	149.0	-	n.d.
4′	-	162.6	-	165.4	-	160.8	-	163.4	-	n.d.	-	150.3
5′	equivalent to 3′ eq. 2′		eq. 3′		eq. 3′		eq. 3′		6.94, d; 8.0	116.4	6.66	117.8
6′			eq. 2′		eq. 2′		eq. 2′		7.55, d; 8.0	120.8	7.55	122.5
OCH_3_									3.92	56.5	3.85	56.3
1″			5.11, d; 7.7	100.4	5.06, d; 7.4	100.5	4.92, d; 9.7	76.4			5.08, d; 7.6	100.7
2″			3.30, pt; 8.5, 8.5	73.7	3.32, n.r.	73.9	n.a., n.r.	n.a.			3.29, pt; 8.1, 8.1	73.9
3″			3.35, pt; 8.9, 8.9 *	77.0	3.34, n.r.	77.2	n.a., n.r.	n.a.			3.35, pt; 8.9, 8.9	77.1
4″			3.22, pt; 9, 9	70.2	3.22, pt; 9, 9	70.2	n.a., n.r.	n.a.			3.22, pt; 9.1, 9.1	70.3
5″			3.49, n.r.	77.8	3.43, n.r.	77.7	n.a., n.r.	n.a.			3.48, n.r.	77.8
6″			3.76/3.53, d; 11.4/n.r.	61.2	3.74/3.53, d; 11.5/n.r.	61.1	n.a./n.a., n.r./n.r.	n.a.			3.76/3.51, d; 11.1/n.r.	61.3
1‴							4.90, d; 9.7	75.4				
2‴							n.a., n.r.	n.a.				
3‴							n.a., n.r.	n.a.				
4‴							n.a., n.r.	n.a.				
5‴							n.a., n.r.	n.a.				
6‴							n.a./n.a., n.r./n.r.	n.a.				

DMSO: 2.55 ppm/40.45 ppm; pt = pseudo t; n.d. = not detected; n.r. = not resolved; n.a. = not assigned. Error of *J*-values may be 0.5 Hz, or a bit more; * = severe line-broadening, not accurate; ** = cross-peak signal with very low intensity at position of H-3. ^a^ = signals of 2″–6″ and 2‴–6‴ were assigned tentatively, by analogy with a mono-substituted lut. Their assignment to a specific glucose moiety was not attempted, except for 1″ and 1‴. Assignment of ″ and ‴ moieties is arbitrary; ^b^ = ^1^H signals: only multiplicities other than s are mentioned; ^c^ = DMSO-*d*_6_–MeOH-*d*_4_ 1:3 (*v*/*v*) at 300 K, with *J*-values taken from spectrum in DMSO-*d*_6_ at 320 K.

## Data Availability

Additional data can be found in the Supplementary Materials.

## Supplementary Materials

The following are available online. Figures S1–S67: 1-dim and 2-dim NMR spectra of all 12 flavones; Figures S68–S69: UHPLC profiles of duplicate injections of extracted wool samples; Figure S70: HPLC profile of weld extract; Figures S71 and S72: photos of weld and onion dyed wool; Figures S73–S75: on-line UV spectra of luteolin, apigenin and chrysoeriol; Figures S76–S84: EICs and TIC of HPLC-DAD-MS analysis of weld extract; Table S1: High-resolution mass spectrometric data of all 12 flavones.

## Sample Availability

Samples of the compounds are not available from the authors.
