# Supplementary material for: Microextraction of Reseda luteola-Dyed Wool and Qualitative Analysis of Its Flavones by UHPLC-UV, NMR and MS
Source: Molecules. 2021 Jun 22;26(13):3787. doi: 10.3390/molecules26133787 (PMC8270335; doi:10.3390/molecules26133787)
Supplement: Supplementary file 1 [file molecules-26-03787-s001.zip › molecules-1245700-supplementary.pdf]

Supporting Information belonging to the paper

Microextraction of *Reseda luteola*-Dyed Wool and Qualitative Analysis of its Flavones by UHPLC-UV, NMR and MS*

by

Elbert van der Klift, Alexandre Villela, Goverdina C.H. Derksen, Peter P. Lankhorst and Teris A. van Beek

Index

Figure S1-S3: NMR spectra luteolin

Figure S4-S7: NMR spectra apigenin

Figure S8-S13: NMR spectra chrysoeriol

Figure S14-S19: NMR spectra luteolin-7-*O*-β-D-glucoside

Figure S20-S25: NMR spectra apigenin-7-*O*-β-D-glucoside

Figure S26-S32: NMR spectra chrysoeriol-7-*O*-β-D-glucoside

Figure S33-S38: NMR spectra apigenin-4'-*O*-β-D-glucoside

Figure S39-S44: NMR spectra luteolin-4'-*O*-β-D-glucoside

Figure S45-S50: NMR spectra luteolin-3'-*O*-β-D-glucoside

Figure S51-S55: NMR spectra apigenin-6,8-di-*C*-β-D-glucoside

Figure S56-S61: NMR spectra luteolin-7,4'-di-*O*-β-D-glucoside

Figure S62-S67: NMR spectra luteolin-7,3'-di-*O*-β-D-glucoside

Figures S68-S69: UHPLC profiles of duplicate injections of extracted wool samples

Figure S70: HPLC profile of weld extract

Figure S71-72: photos of weld and onion dyed wool

Figure S73-S75: on-line UV spectra of luteolin, apigenin and chrysoeriol

Figure S76-S84: EICs and TIC of HPLC-DAD-MS analysis of weld extract

Table S1: High-resolution mass spectrometric data of all 12 flavones

Luteolin (**lut**) **8**

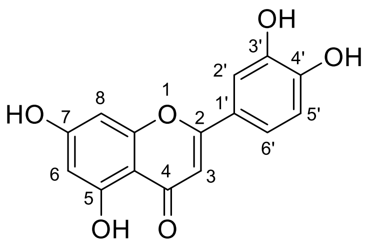

luteolin

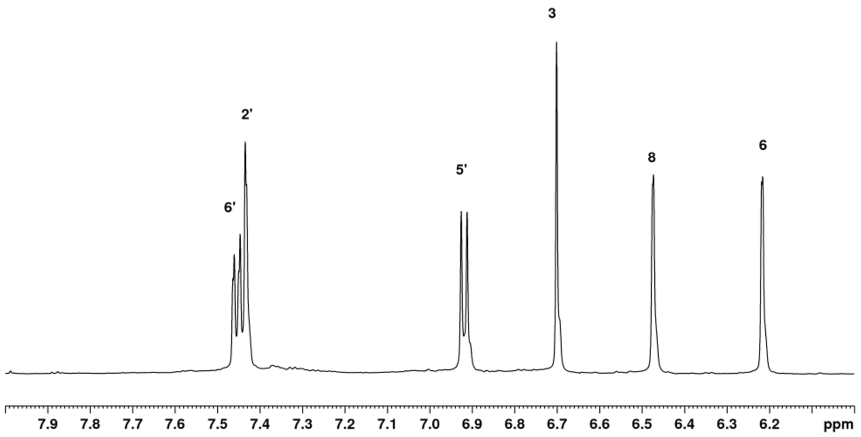

Figure SI 1. ^{1}H-NMR spectrum of **lut**.

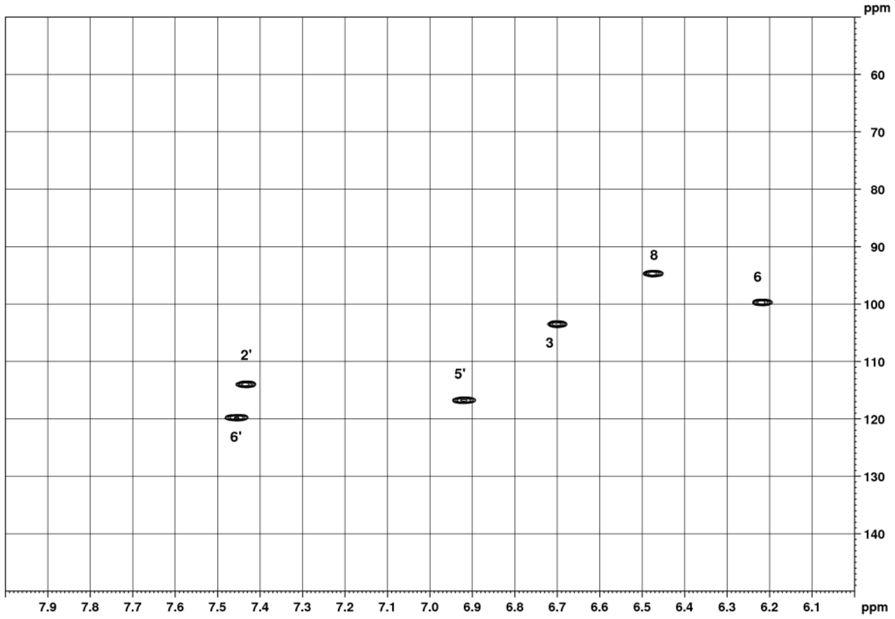

Figure SI 2. HSQC spectrum of **lut**.

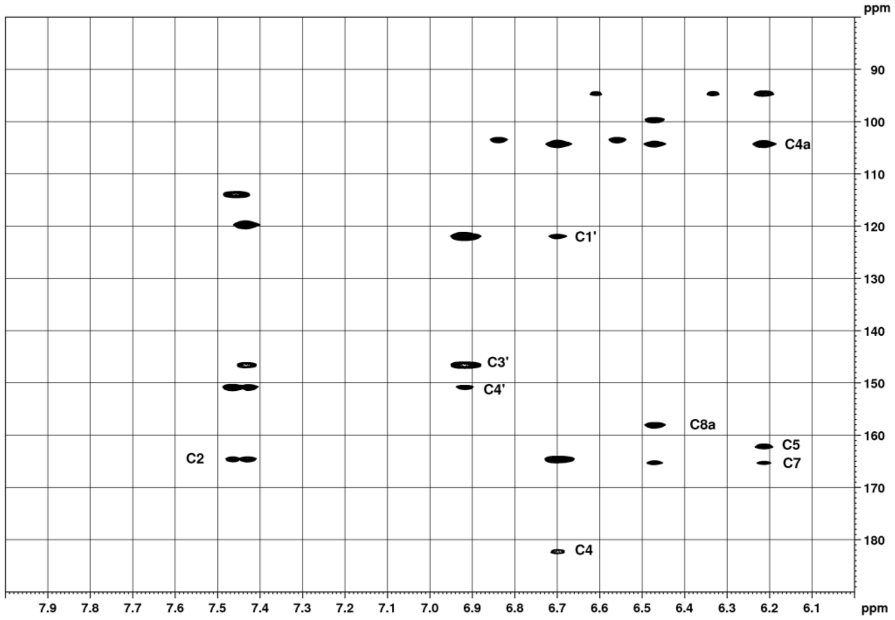

Figure SI 3. HMBC spectrum of **lut**; quaternary C-atoms assigned.

Apigenin (**api**) 9

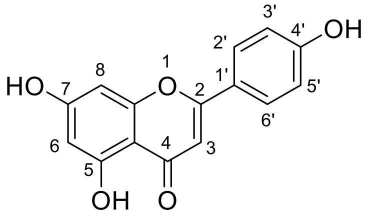

apigenin

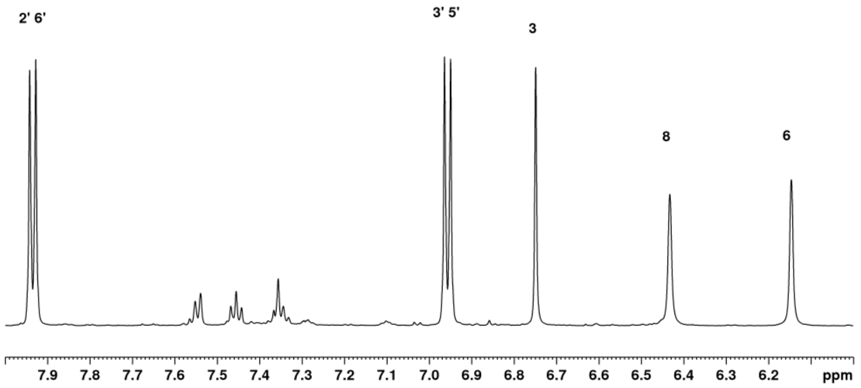

Figure SI 4. ^{1}H-NMR spectrum of **api**.

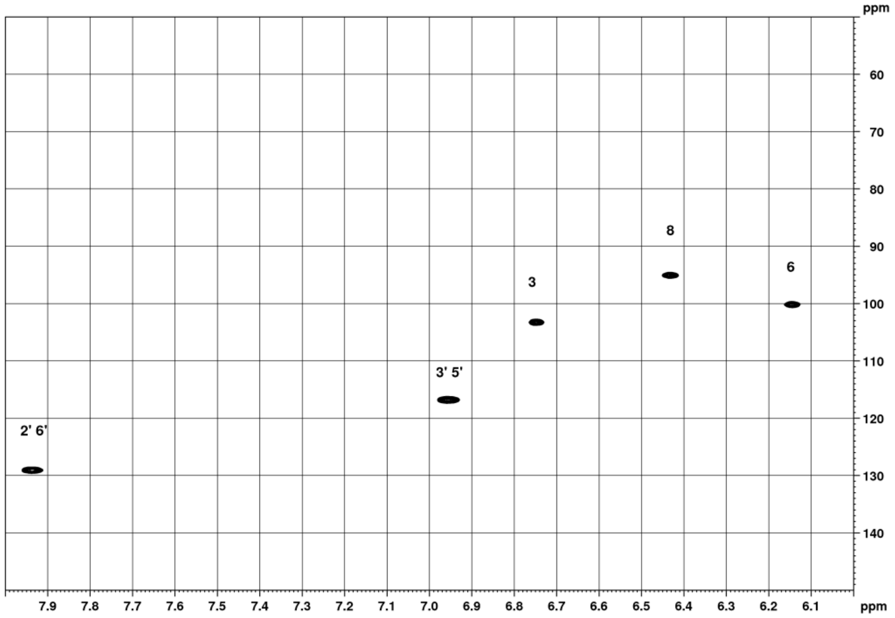

Figure SI 5. HSQC spectrum of **api**.

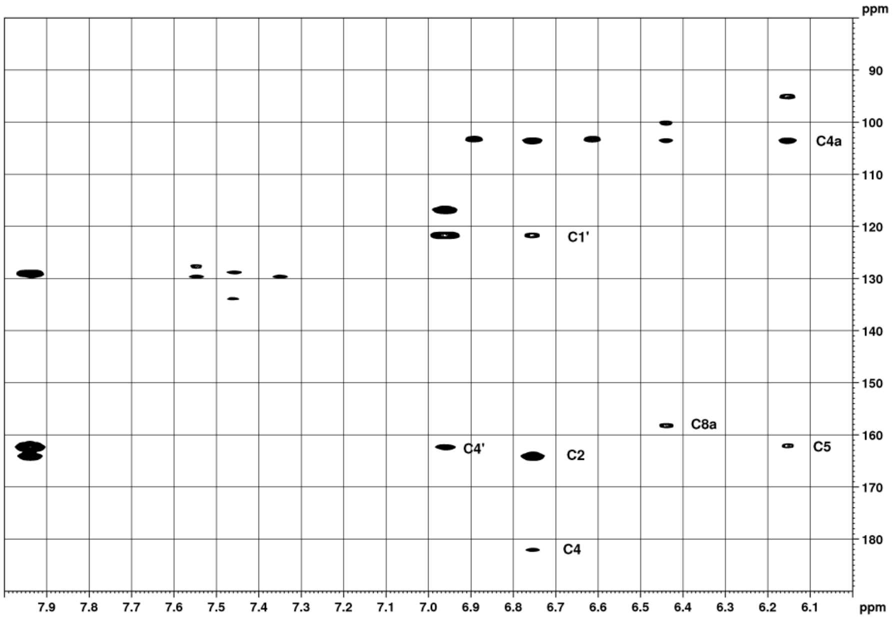

Figure SI 6. HMBC spectrum of **api**; quaternary C-atoms assigned.

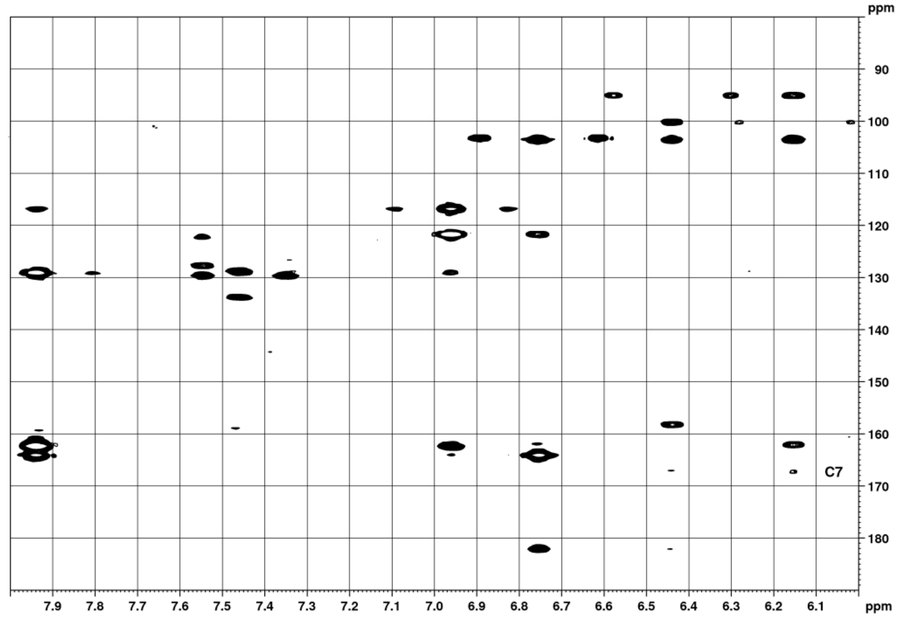

Figure SI 7. HMBC spectrum of **api**—same as Fig. SI 6, but at a lower contour level; C-7 assigned.

Chrysoeriol (**chry**) 10

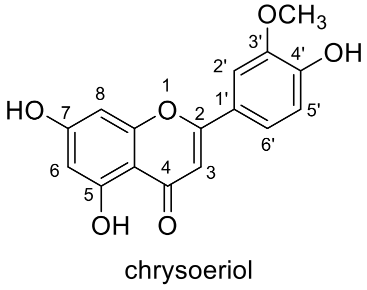

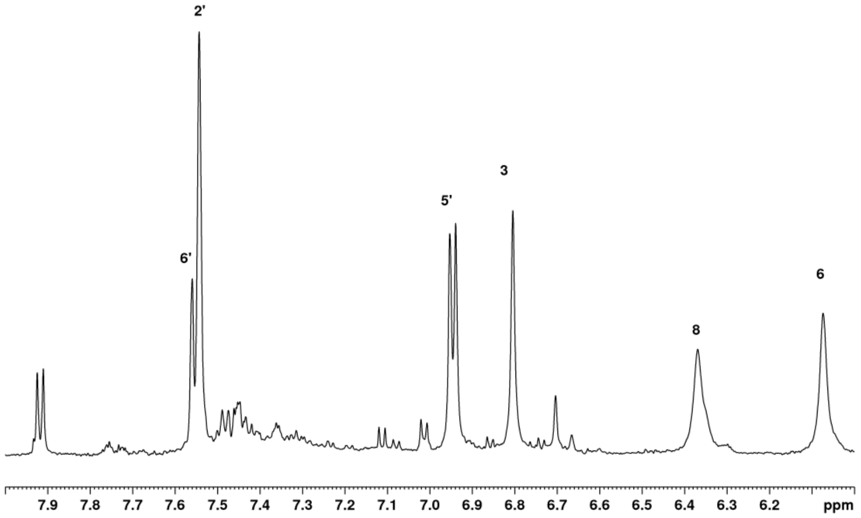

Figure SI 8. ^{1}H-NMR spectrum of **chry**.

The signals at 7.9 and 6.7 ppm suggested the presence of **api** as an impurity in this fraction. This was confirmed by HPLC–UV and HRMS, and is caused by the similar polarity of both compounds—as seen by the fact that they elute close to each other in RP-HPLC (Fig. SI 70).

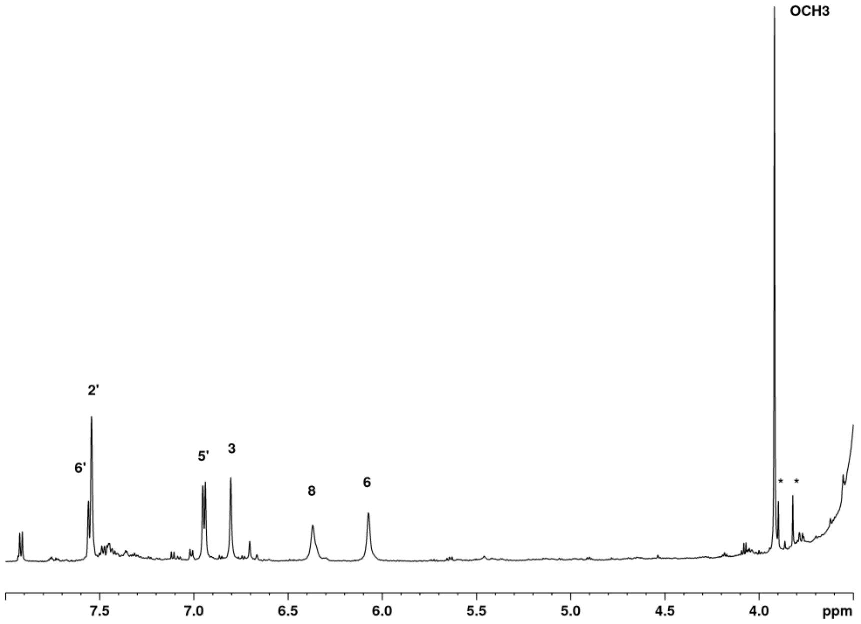

Figure SI 9. ^{1}H-NMR spectrum of **chry**; * = signals of impurities.

Concerning the impurities: see Figs. SI 12 and 13 below, and ensuing discussion.

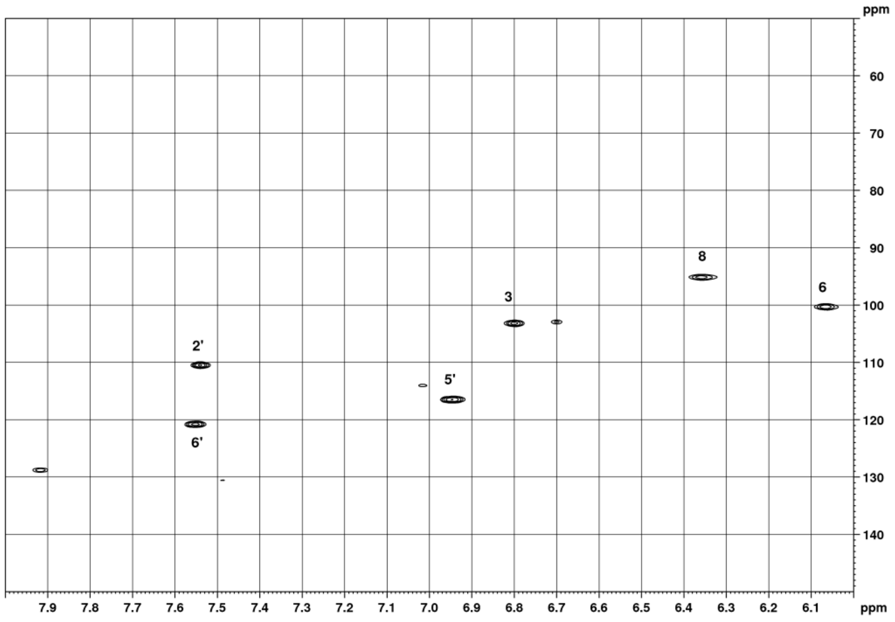

Figure SI 10. HSQC spectrum of **chry**.

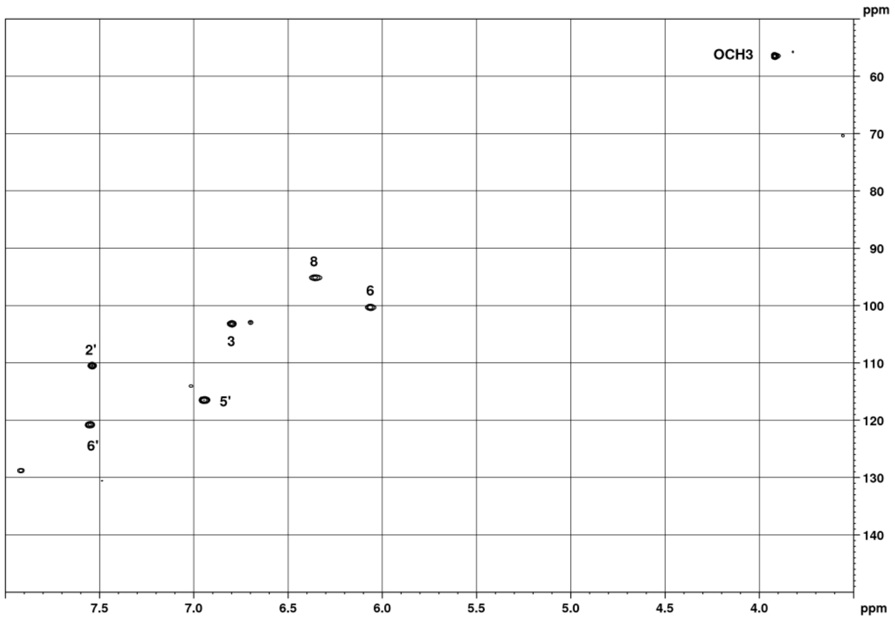

Figure SI 11. HSQC spectrum of **chry**.

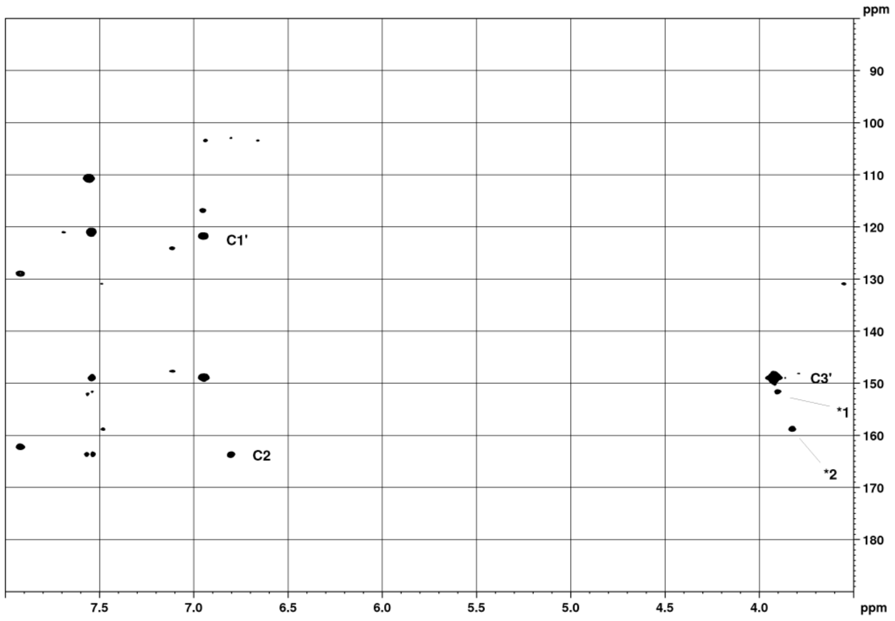

Figure SI 12. HMBC spectrum of **chry**.

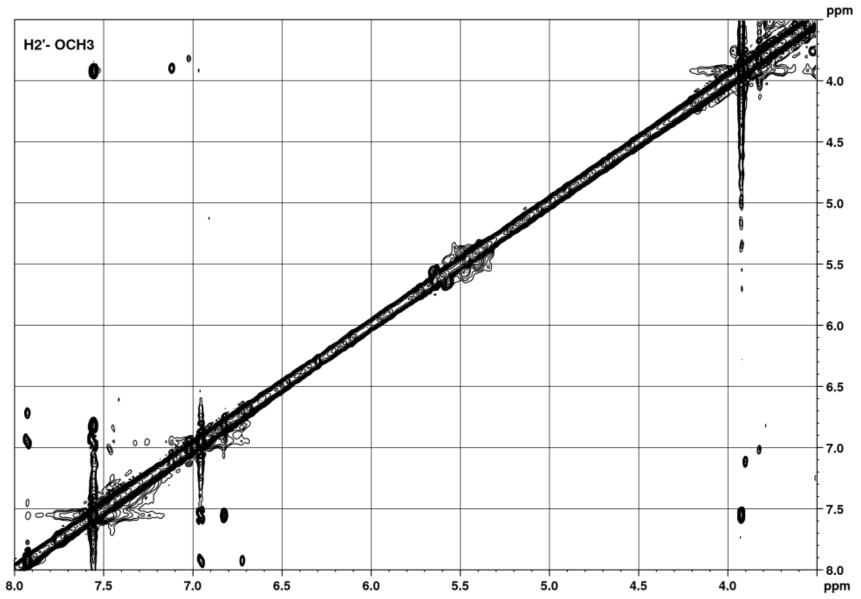

Figure SI 13. ROESY spectrum of **chry**.

The ROESY spectrum shows a strong cross-peak between the OCH_3 and H-2′, which confirms that the methoxyl group is attached to C-3′, and not to C-4′. The OCH_3 of the two impurities have cross-peaks around 7 ppm, which is close to H-5′ of **chry**. This suggests that the two cross-peaks might arise from a compound with a methoxyl group on C-4′, and maybe a compound with two of these groups, on C-4′ and C-3′.

Luteolin-7-*O*-β-D-glucoside (**lut**-7-*O*-glu) **4**

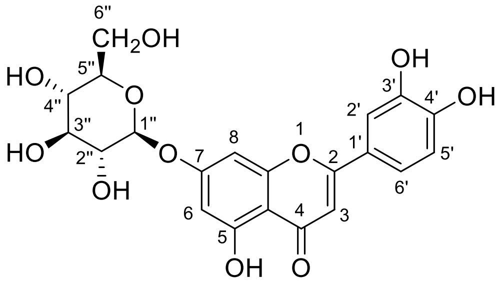

luteolin-7-O-β-D-glucopyranoside

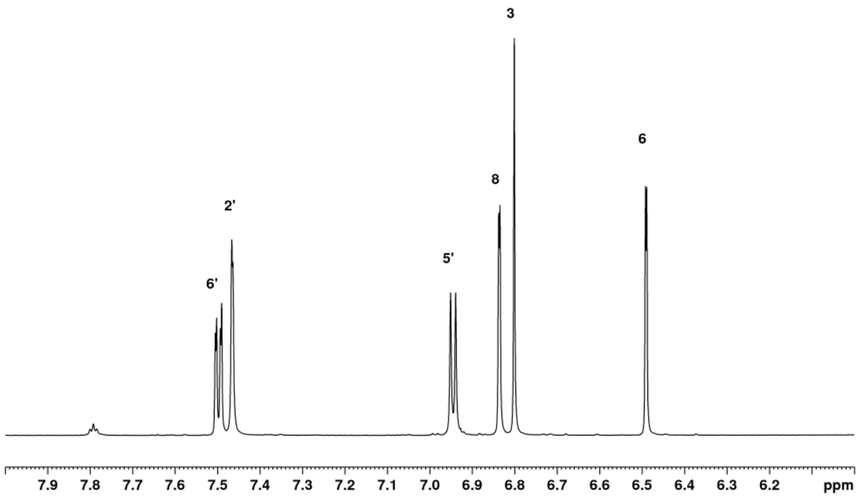

Figure SI 14. ^{1}H-NMR spectrum of **lut**-7-*O*-glu.

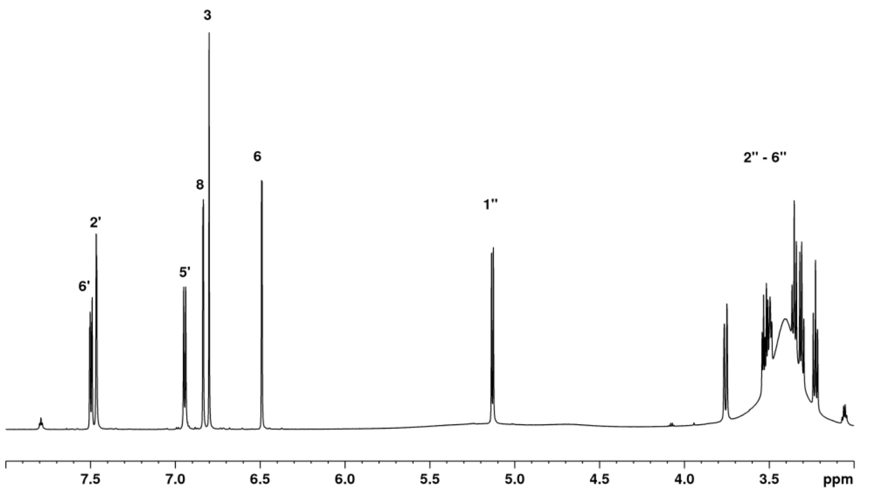

Figure SI 15. ^{1}H-NMR spectrum of **lut**-7-*O*-glu; glucose signals displayed.

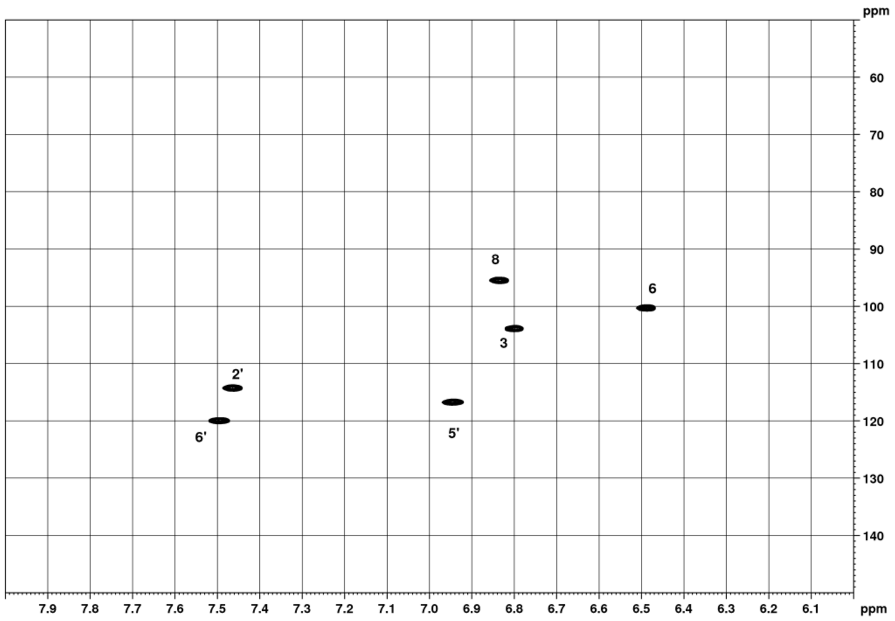

Figure SI 16. HSQC spectrum of **lut**-7-*O*-glu.

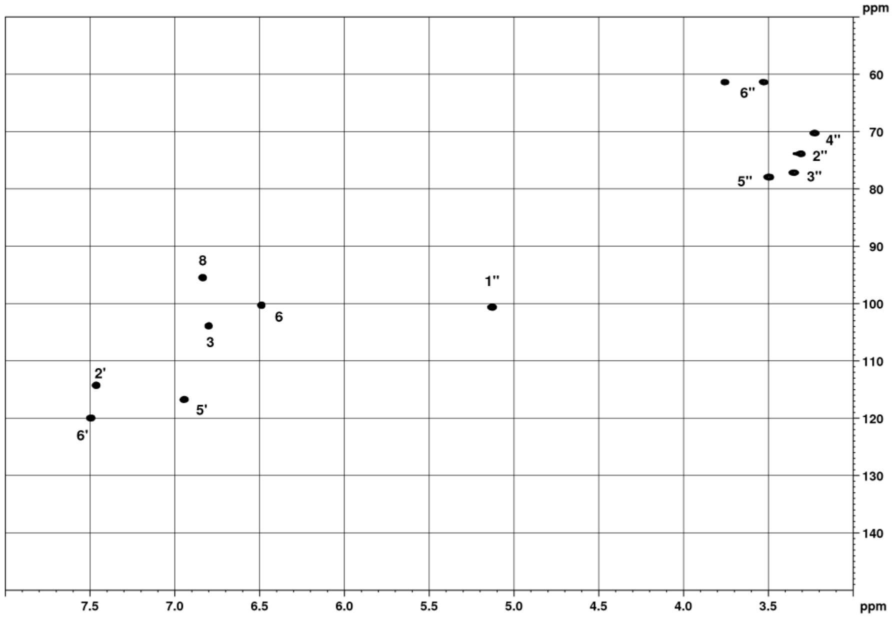

Figure SI 17. HSQC spectrum of **lut**-7-*O*-glu; glucose signals displayed.

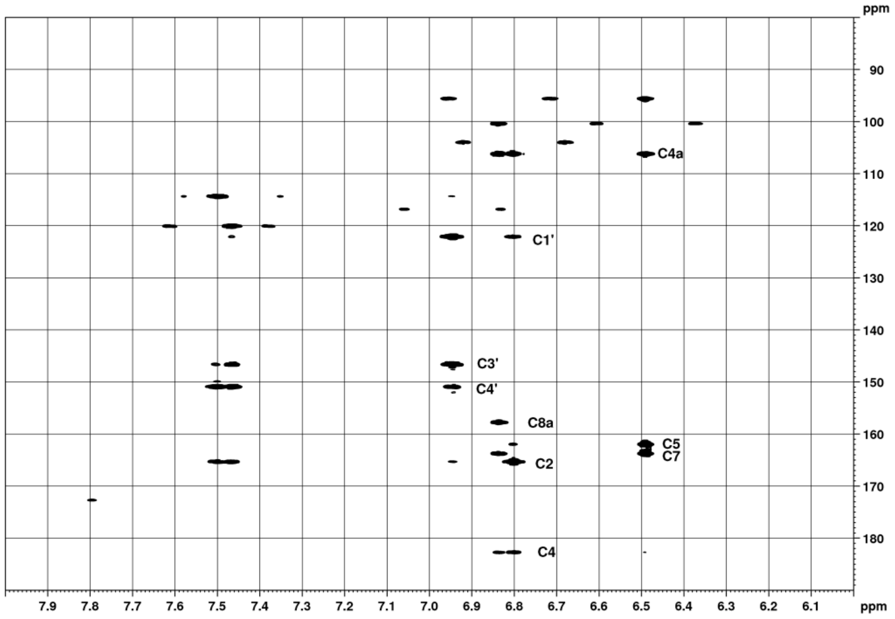

Figure SI 18. HMBC spectrum of **lut**-7-*O*-glu.

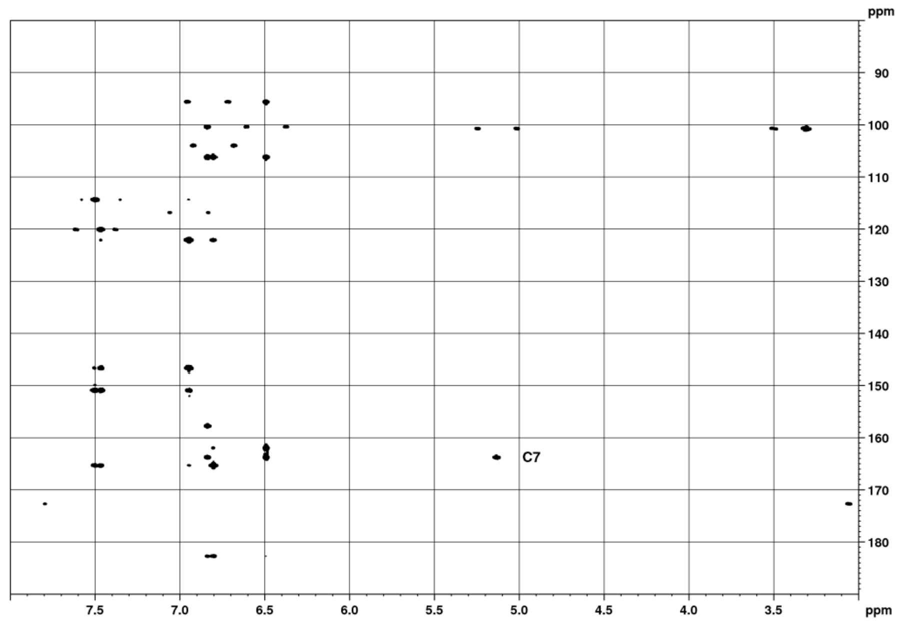

Figure SI 19. HMBC spectrum of **lut**-7-*O*-glu; glucose signals displayed.

Figure SI 19 shows a correlation between C-7 and H-1″. This confirms that the glucose moiety is attached to O-7.

Apigenin-7-*O*-β-D-glucoside (**api**-7-*O*-glu) **5**

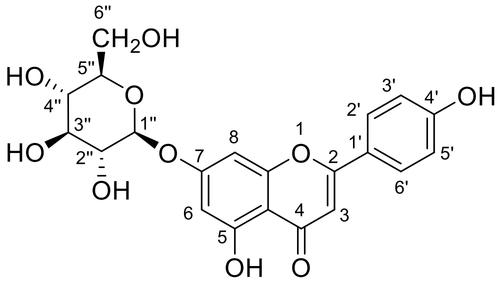

apigenin-7-O-β-D-glucopyranoside

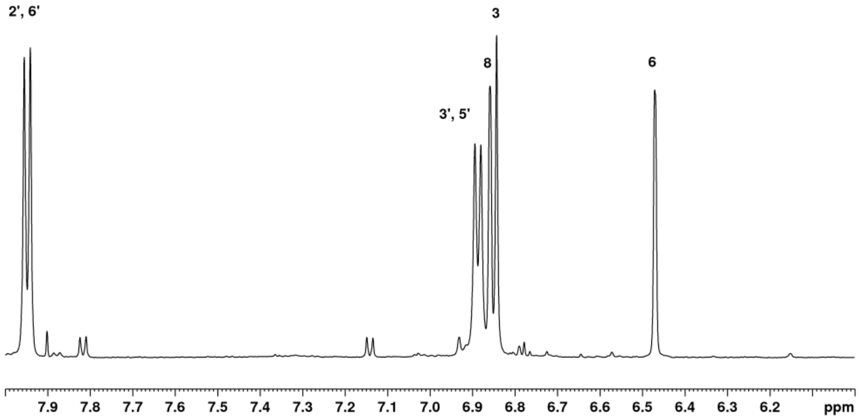

Figure SI 20. ^{1}H-NMR spectrum of **api**-7-*O*-glu, with water suppression.

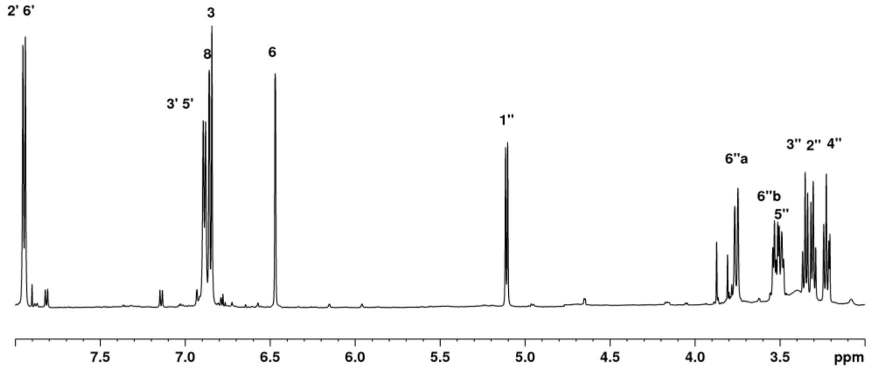

Figure SI 21. ^{1}H-NMR spectrum of **api**-7-*O*-glu, with water suppression; glucose signals displayed.

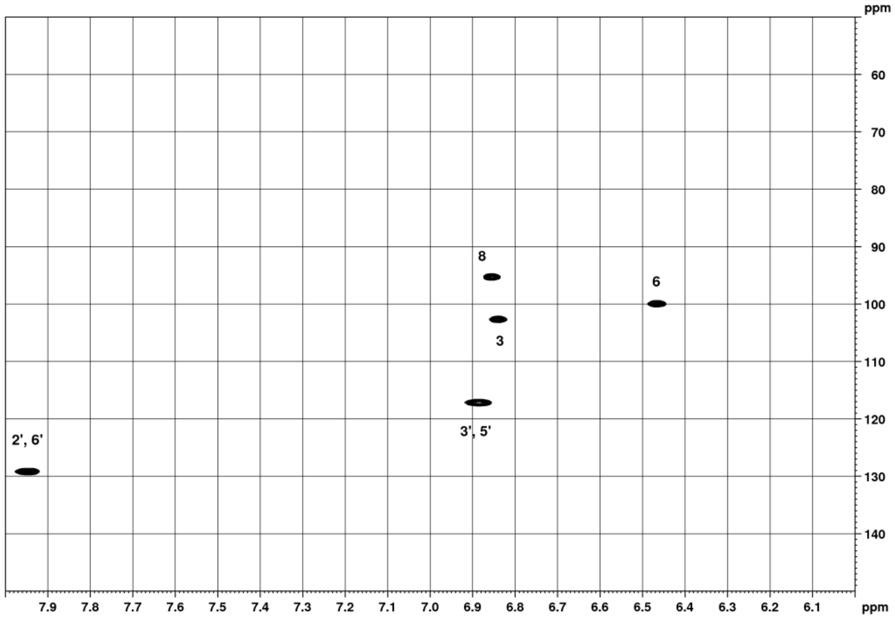

Figure SI 22. HSQC spectrum of **api**-7-*O*-glu.

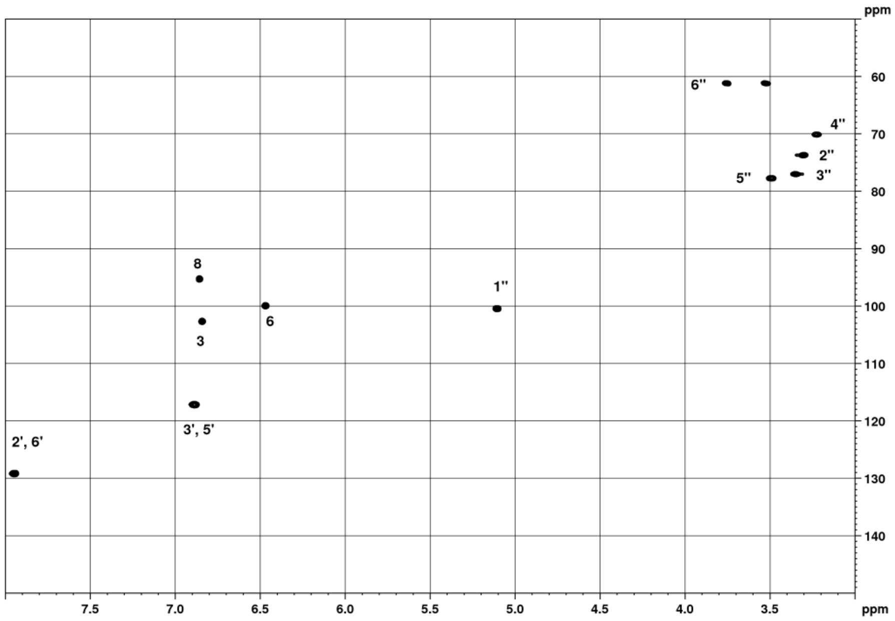

Figure SI 23. HSQC spectrum of **api**-7-*O*-glu; glucose signals displayed.

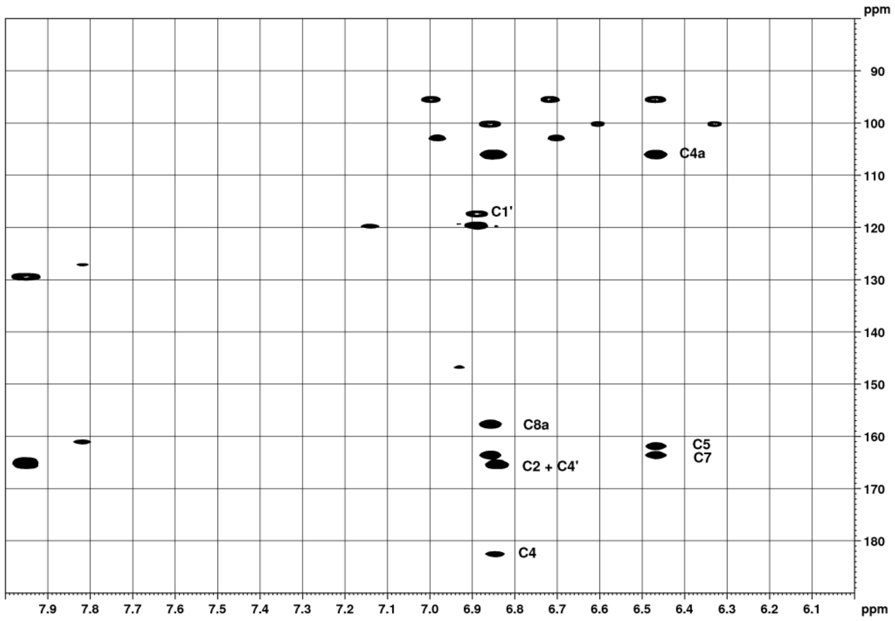

Figure SI 24. HMBC spectrum of **api**-7-*O*-glu.

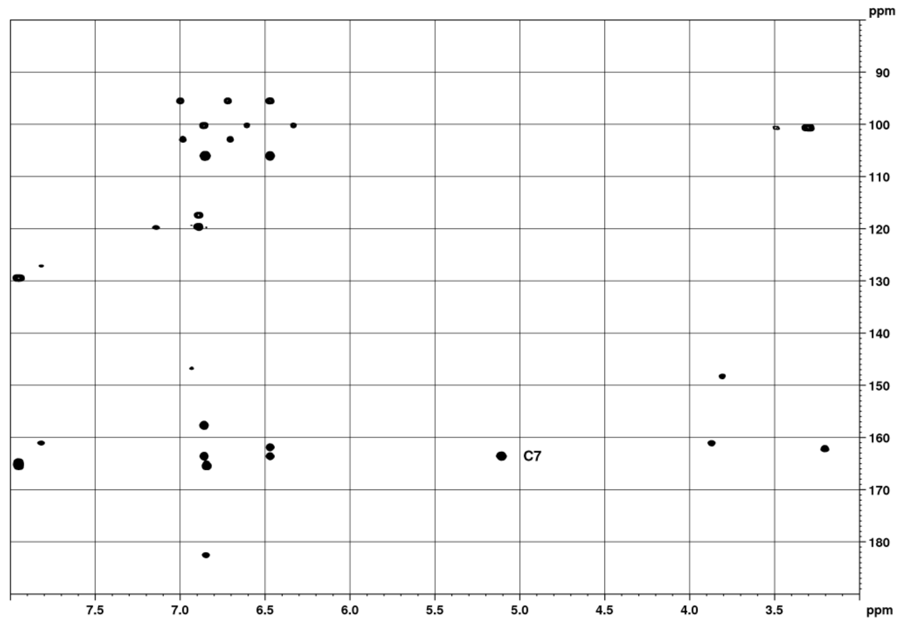

Figure SI 25. HMBC spectrum of **api**-7-*O*-glu; glucose signals displayed.

Figure SI 25 shows a correlation between C-7 and H-1″. This confirms that the sugar moiety is attached to O-7.

Chrysoeriol-7-*O*-β-D-glucoside (**chry**-7-*O*-glu) **6**

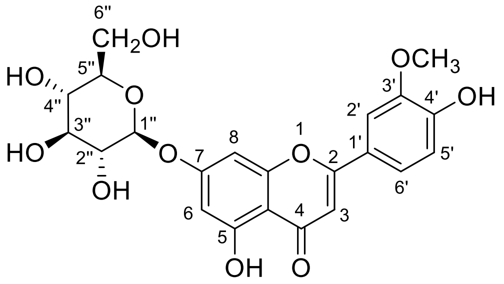

chrysoeriol-7-O-β-D-glucopyranoside

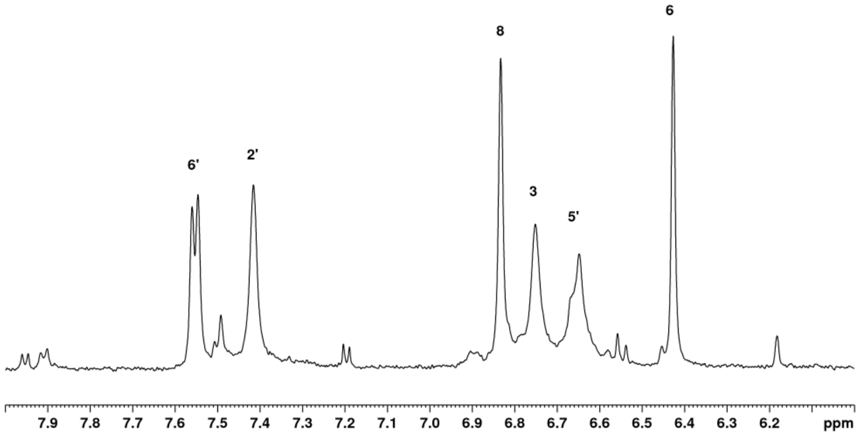

Figure SI 26. ^{1}H-NMR spectrum of **chry**-7-*O*-glu, with water suppression.

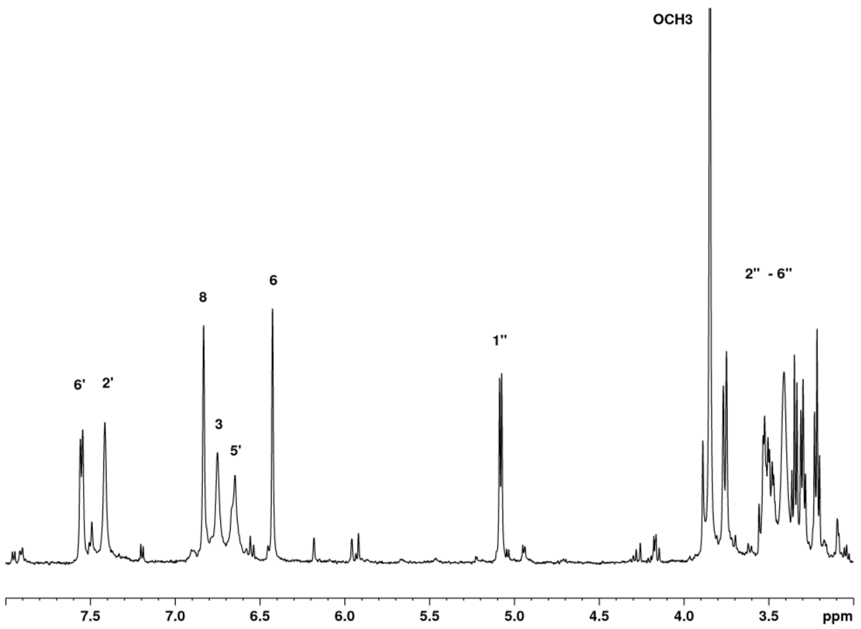

Figure SI 27. ^{1}H-NMR spectrum of **chry**-7-*O*-glu, with water suppression; glucose signals displayed.

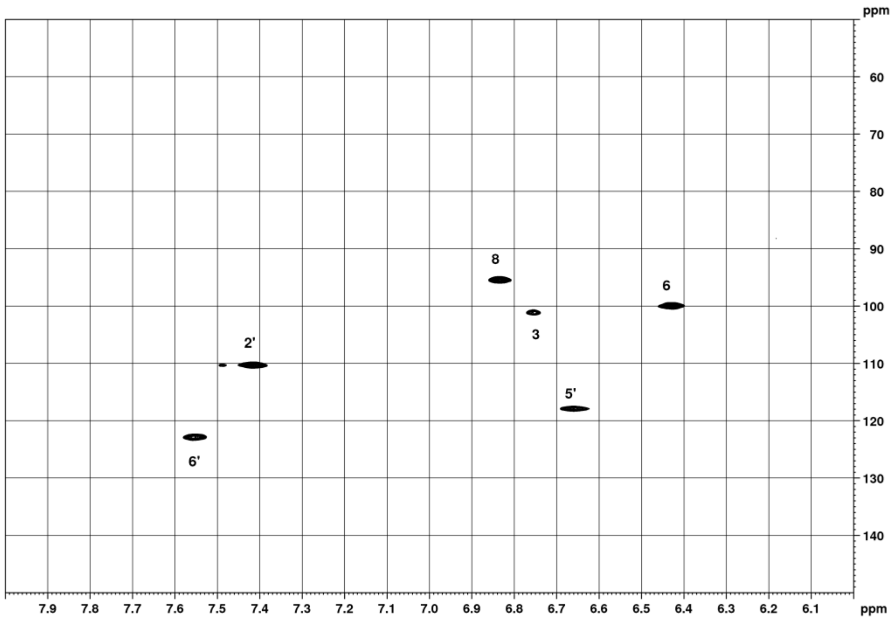

Figure SI 28. HSQC spectrum of **chry**-7-*O*-glu.

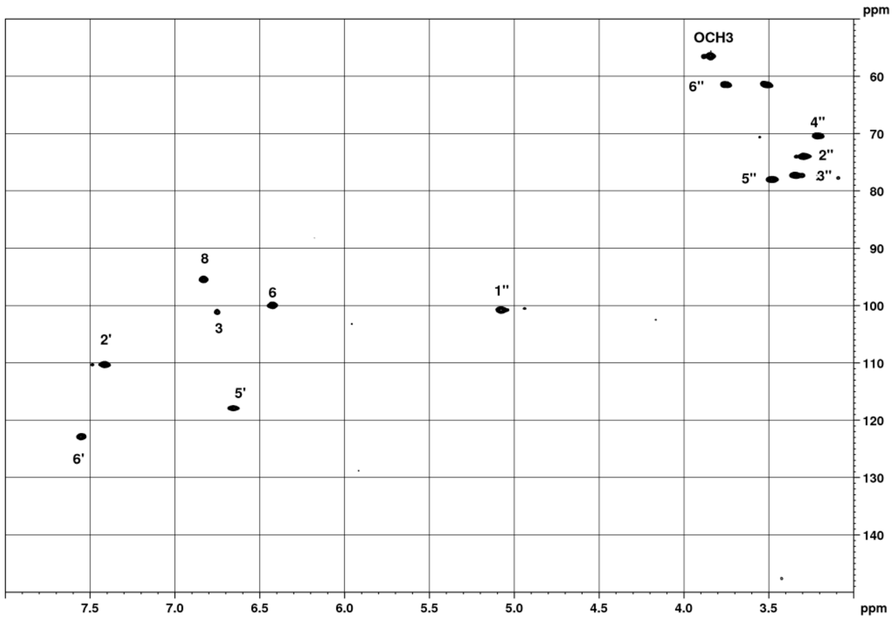

Figure SI 29. HSQC spectrum of **chry**-7-*O*-glu; glucose signals displayed.

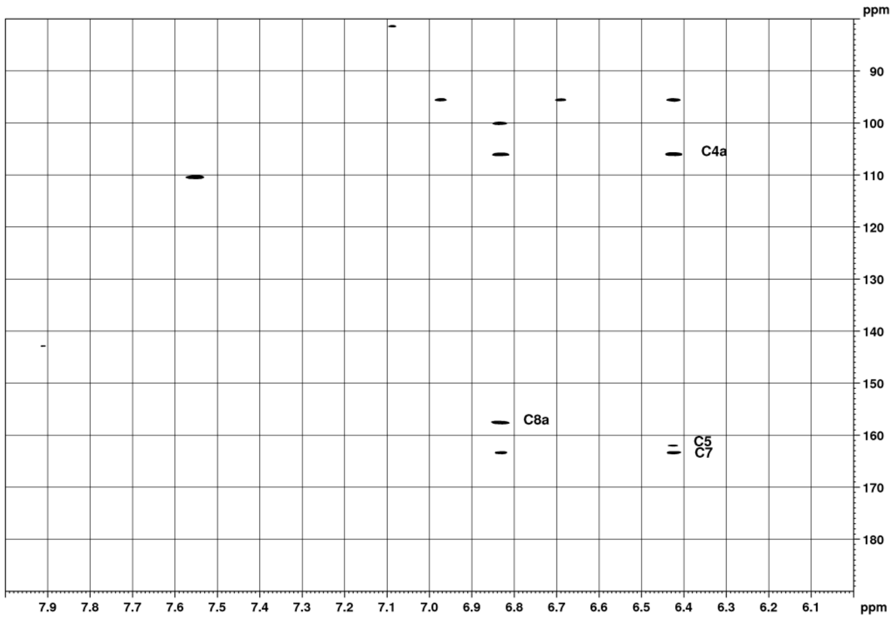

Figure SI 30. HMBC spectrum of **chry**-7-*O*-glu.

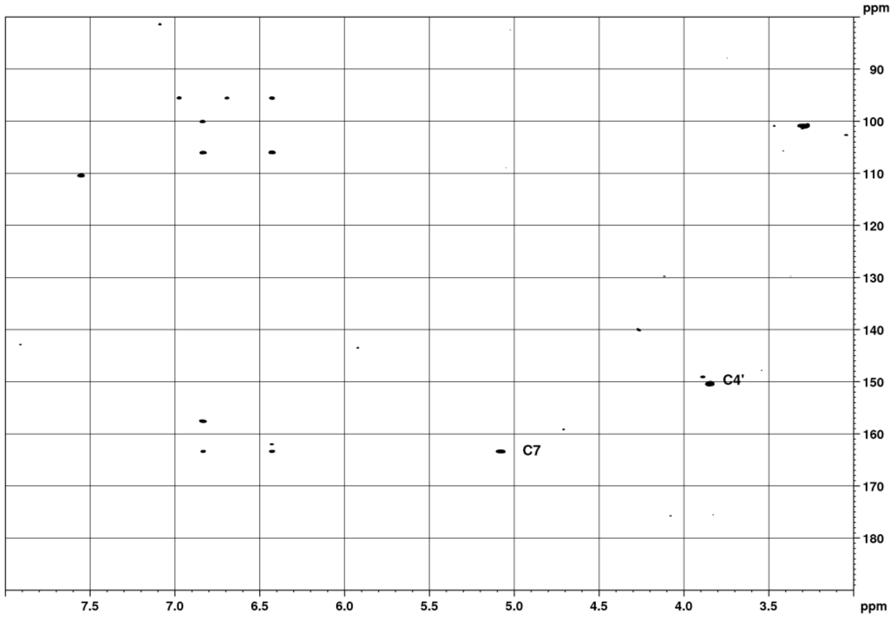

Figure SI 31. HMBC spectrum of **chry**-7-*O*-glu; glucose signals displayed.

Figure SI 31 shows a correlation between C-7 and H-1″. This indicates that the sugar moiety is attached to O-7. Due to the small amount isolated not all expected cross-peaks are visible in the HMBC spectrum. On the basis of the HMBC spectrum it could not be decided whether the methoxyl group is attached to C-3′ or C-4′, as they have very similar chemical shifts. However, the assignment to C4′, and not C3′, was made after evaluation of the ROESY spectrum. See Figure SI 32 below.

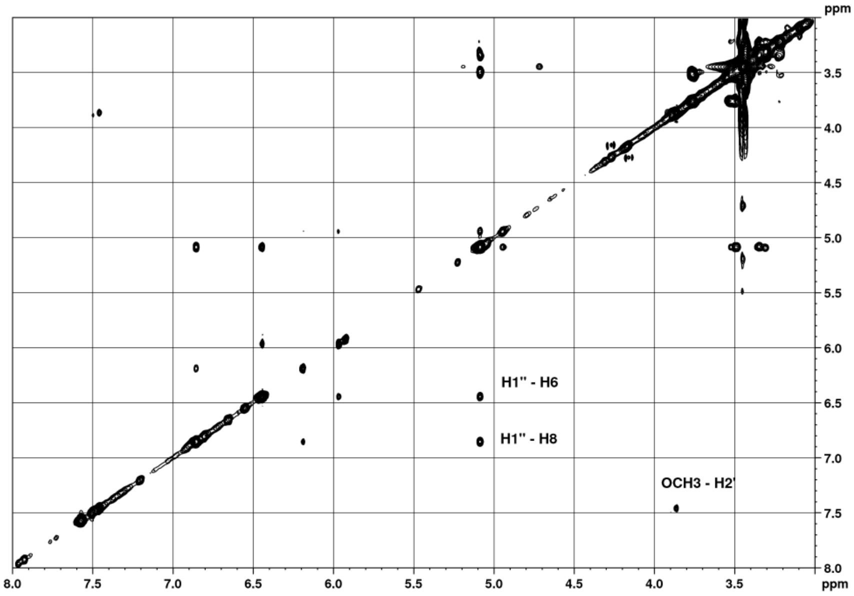

Figure SI 32. ROESY spectrum of **chry**-7-*O*-glu; glucose signals displayed.

The position of the methoxyl group and that of the sugar moiety were confirmed based on the ROESY spectrum in Fig. SI 32. Clear nOe can be seen between H-1″ and both H6 and H8, confirming that sugar moiety is attached to O-7. Additionally, the nOe between OCH_3 and H-2′ confirms that the OCH_3 is attached to C-3′, and not to C-4′.

Apigenin-4′-*O*-β-D-glucoside (**api**-4′-*O*-glu) **tr1**

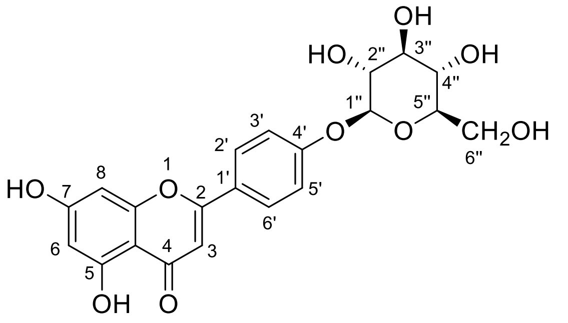

apigenin-4'-O-β-D-glucopyranoside

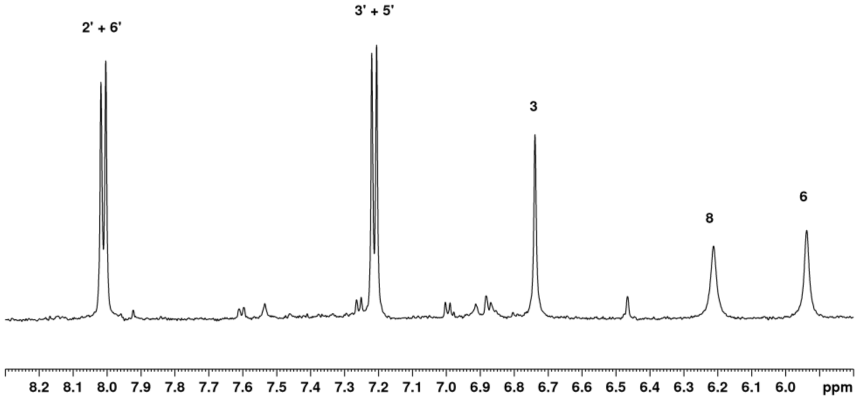

Figure SI 33. ^{1}H-NMR spectrum of **api**-4′-*O*-glu, with water suppression.

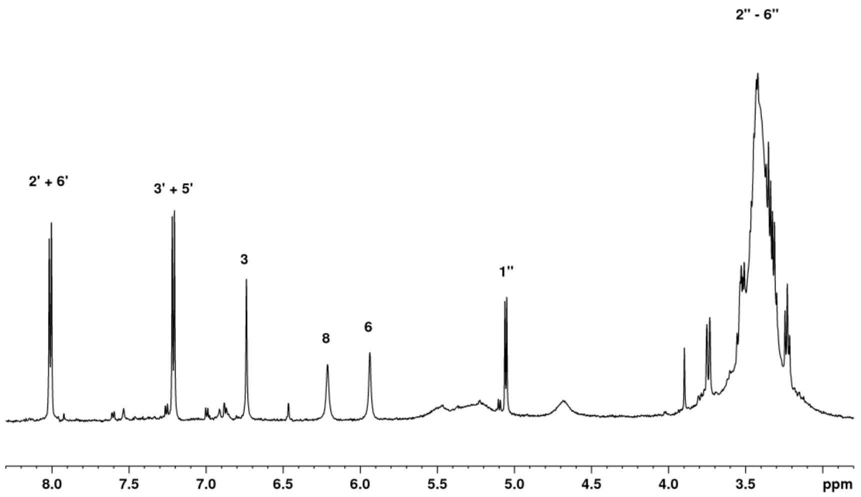

Figure SI 34. ^{1}H-NMR spectrum of **api-**4′-*O*-glu, with water suppression; glucose signals displayed.

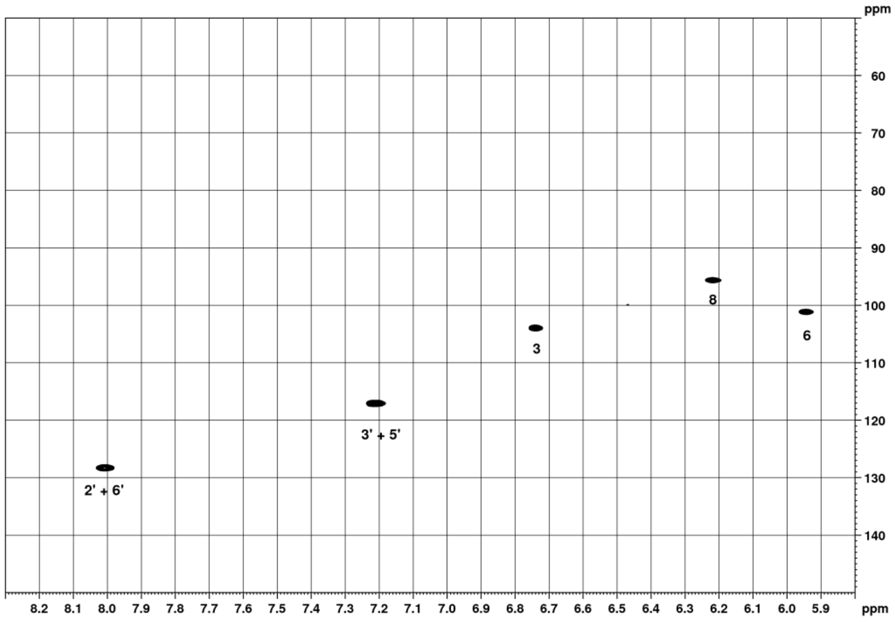

Figure SI 35. HSQC spectrum of **api-**4′-*O*-glu.

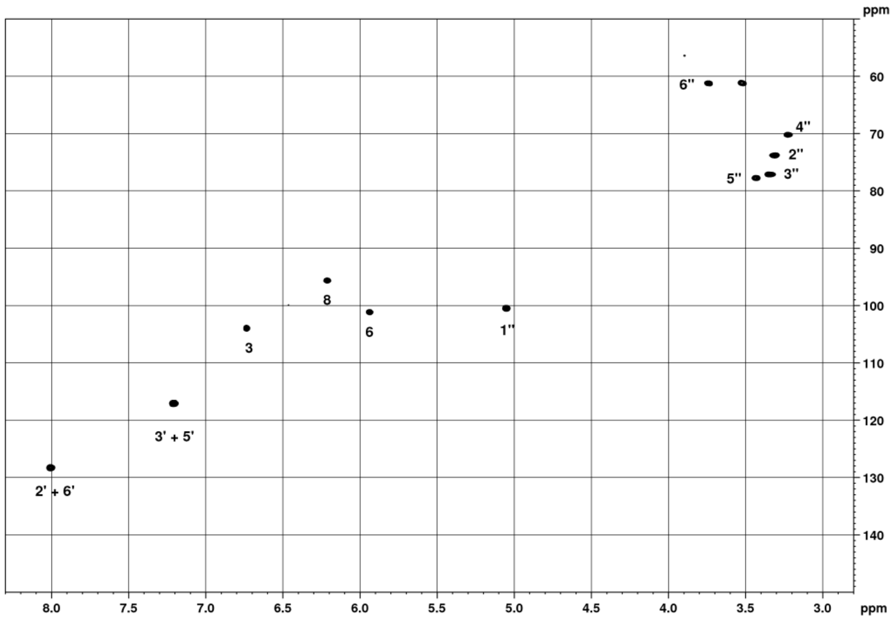

Figure SI 36. HSQC spectrum of **api-**4′-*O*-glu; glucose signals displayed.

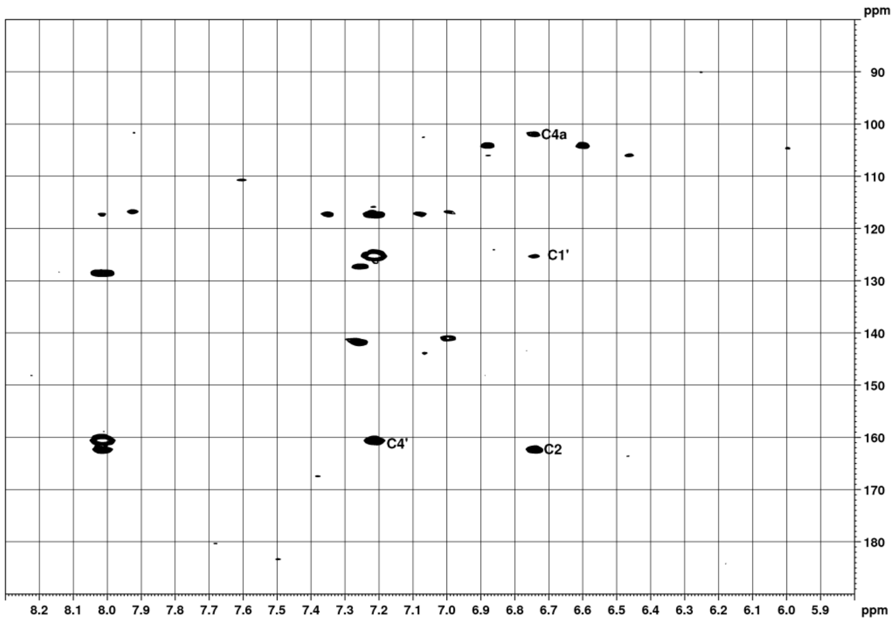

Figure SI 37. HMBC spectrum of **api-**4′-*O*-glu.

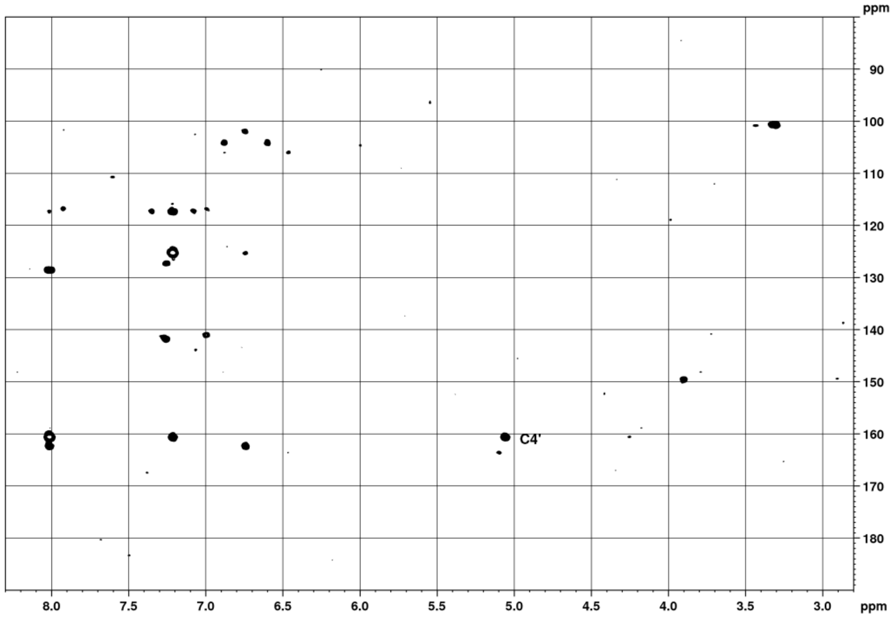

Figure SI 38. HMBC spectrum of **api-**4′-*O*-glu; glucose signals displayed.

Fig. SI 38 shows a correlation between C-4′ and H-1″. This indicates that the sugar moiety is attached to O-4′. Due to the small amount isolated not all expected cross-peaks are visible in the HMBC spectrum.

Luteolin-4′-*O*-β-D-glucoside (**lut**-4′-*O*-glu) **tr2**

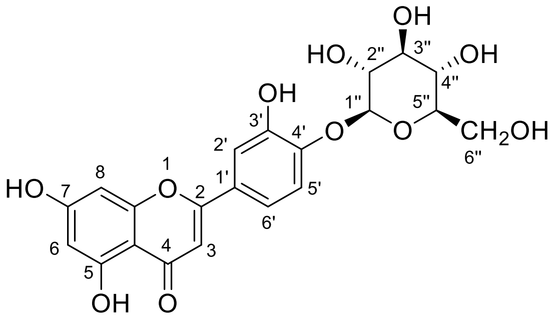

luteolin-4'-O-β-D-glucopyranoside

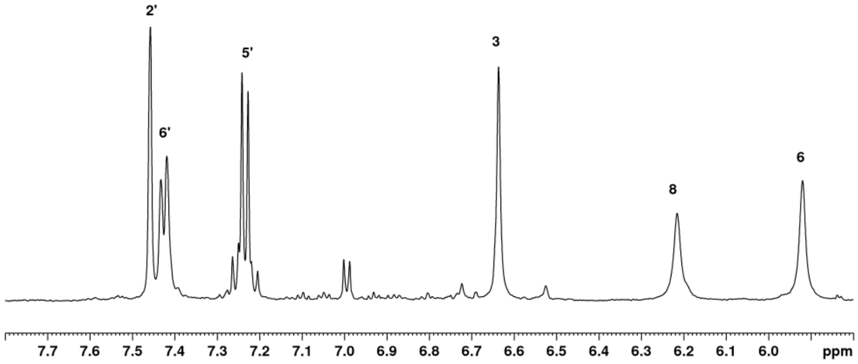

Figure SI 39. ^{1}H-NMR spectrum of **lut**-4′-*O*-glu, with water suppression.

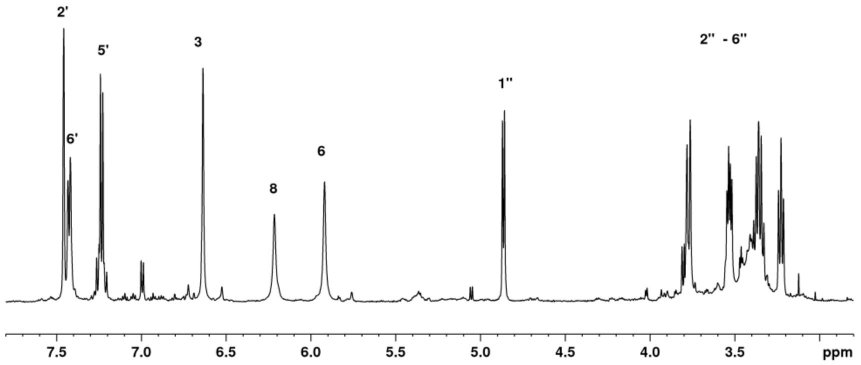

Figure SI 40. ^{1}H-NMR spectrum of **lut**-4′-*O*-glu, with water suppression; glucose signals displayed.

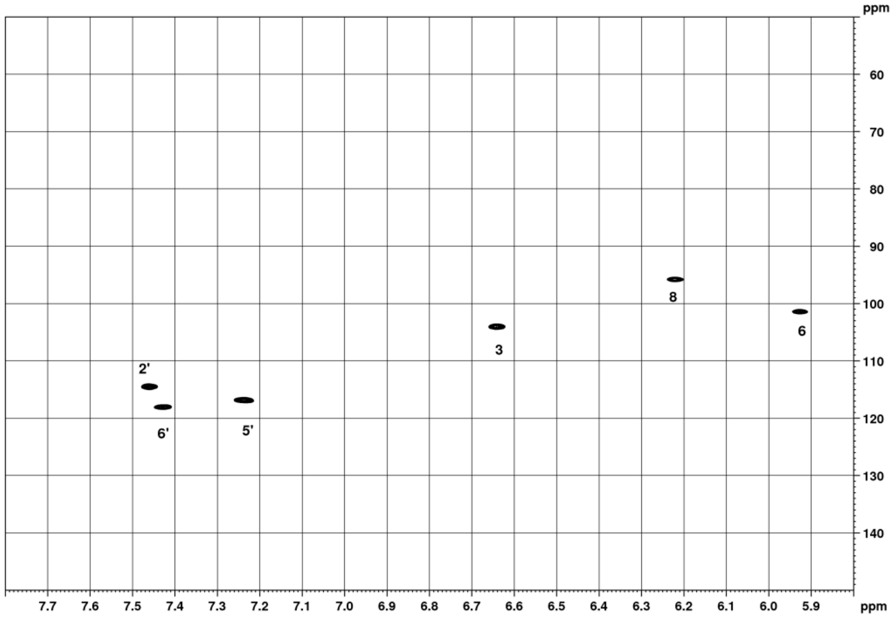

Figure SI 41. HSQC spectrum of **lut-**4′-*O*-glu.

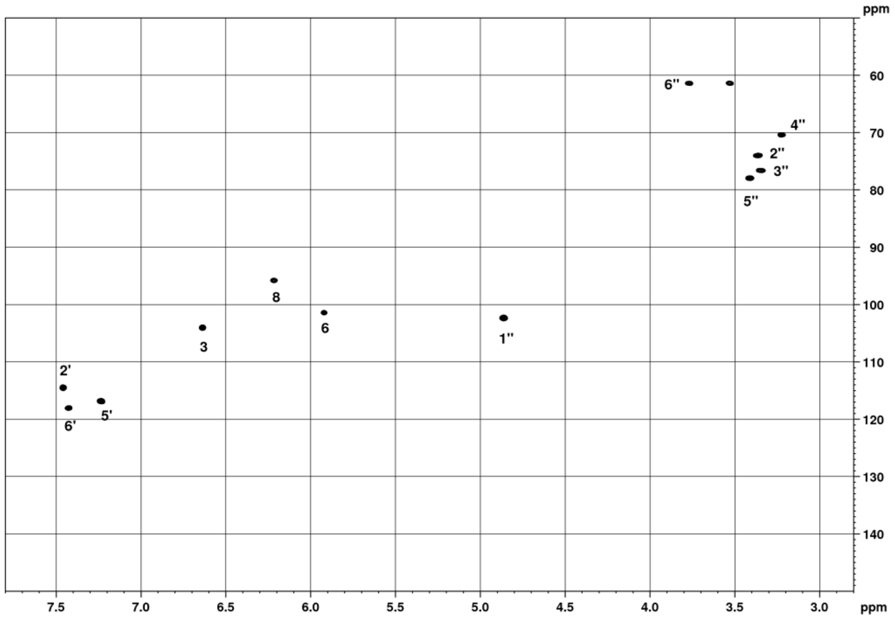

Figure SI 42. HSQC spectrum of **lut-**4′-*O*-glu; glucose signals displayed.

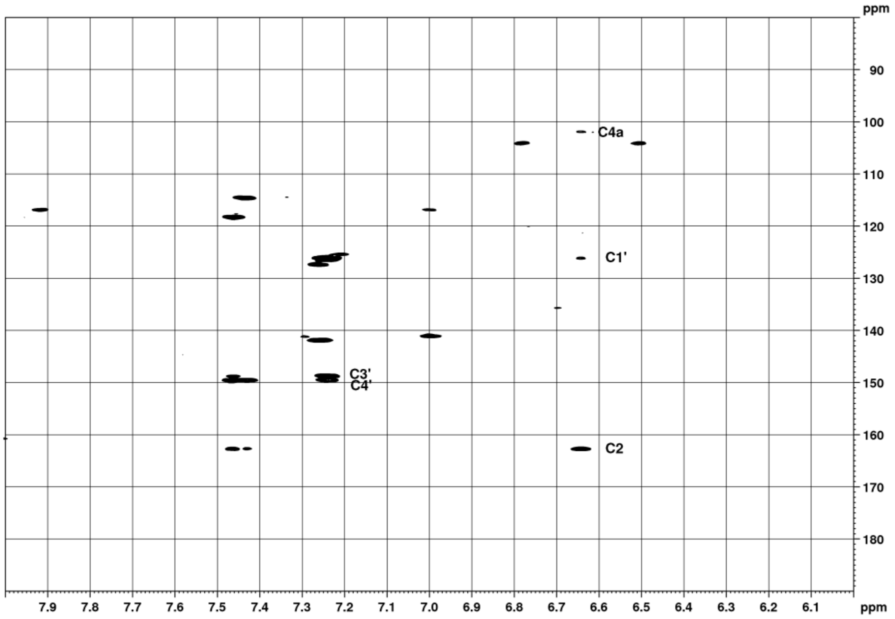

Figure SI 43. HMBC spectrum of **lut-**4′-*O*-glu.

In Fig. SI 43 the signals of C-4′ and C-3′ can be discriminated because one of them (C-4′) shows cross-peaks to both H-2′ and H-6′, and the other one has a cross peak with H-2′ only.

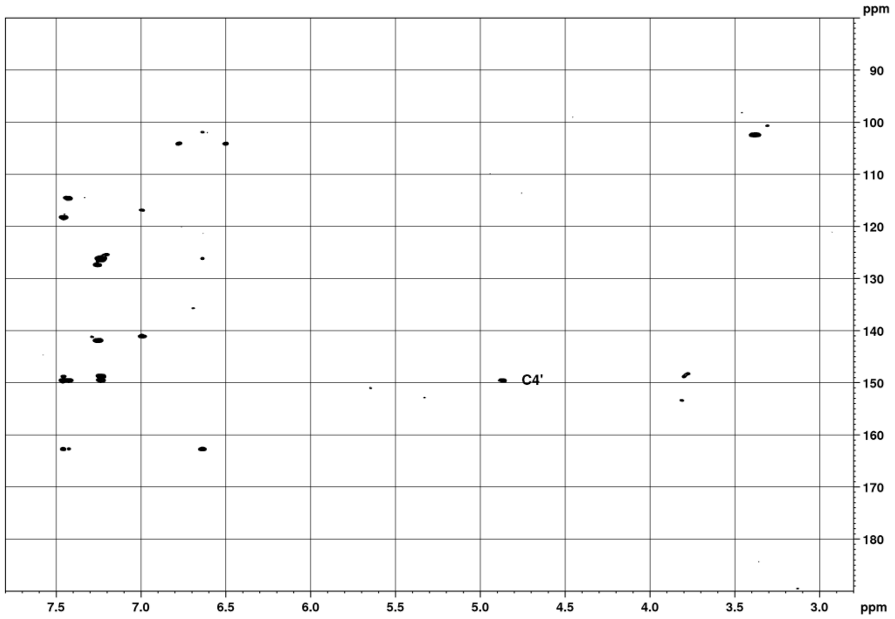

Figure SI 44. HMBC spectrum of **lut-**4′-*O*-glu; glucose signals displayed.

Fig. SI 44 shows a correlation between C-4′ and H-1″. This indicates that the sugar moiety is attached to O-4′. Due to the low quantity of material not all expected cross-peaks are visible in the HMBC spectrum.

Luteolin-3′-*O*-β-D-glucoside (**lut**-3′-*O*-glu) **7**

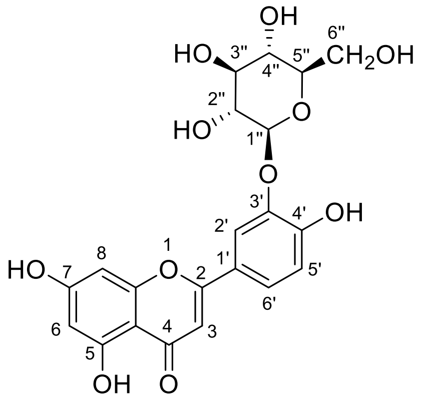

luteolin-3'-O-β-D-glucopyranoside

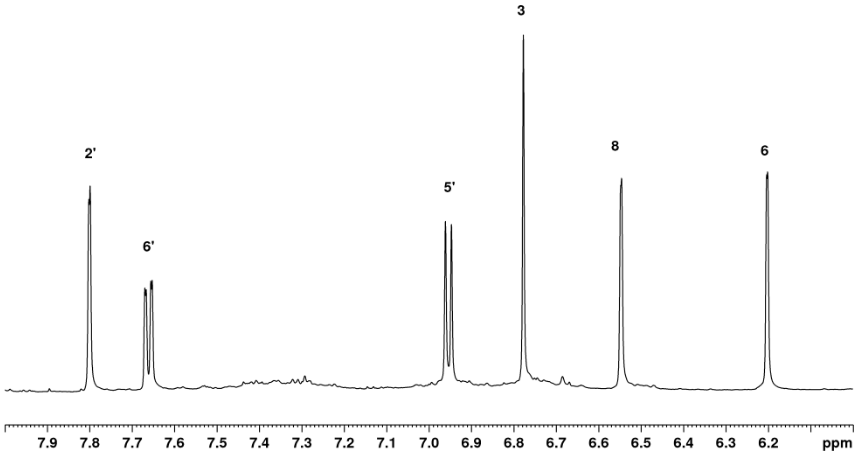

Figure SI 45. ^{1}H-NMR spectrum of **lut**-3′-*O*-glu.

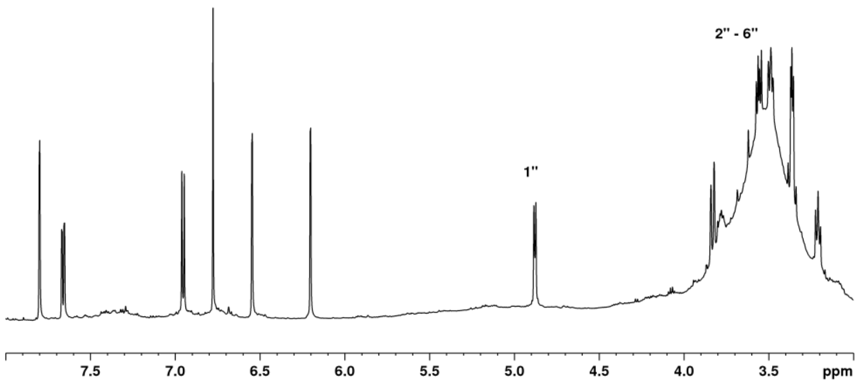

Figure SI 46. ^{1}H-NMR spectrum of **lut**-3′-*O*-glu; glucose signals displayed.

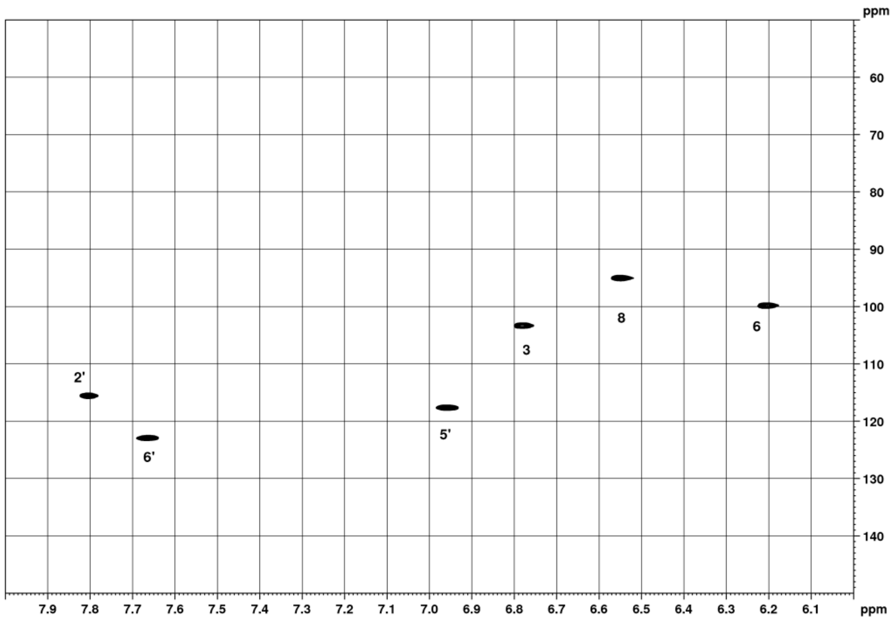

Figure SI 47. HSQC spectrum of **lut**-3′-*O*-glu.

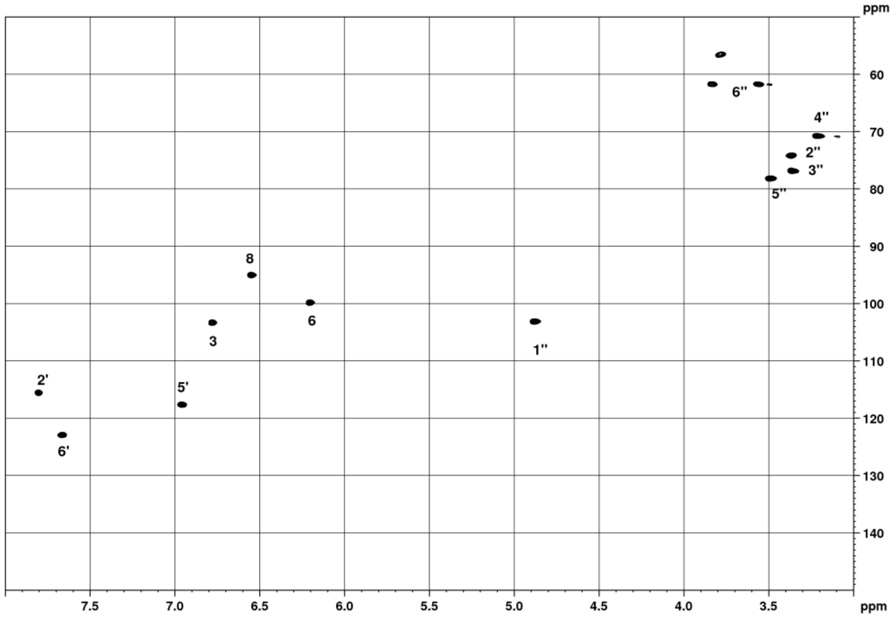

Figure SI 48. HSQC spectrum of **lut**-3′-*O*-glu; glucose signals displayed.

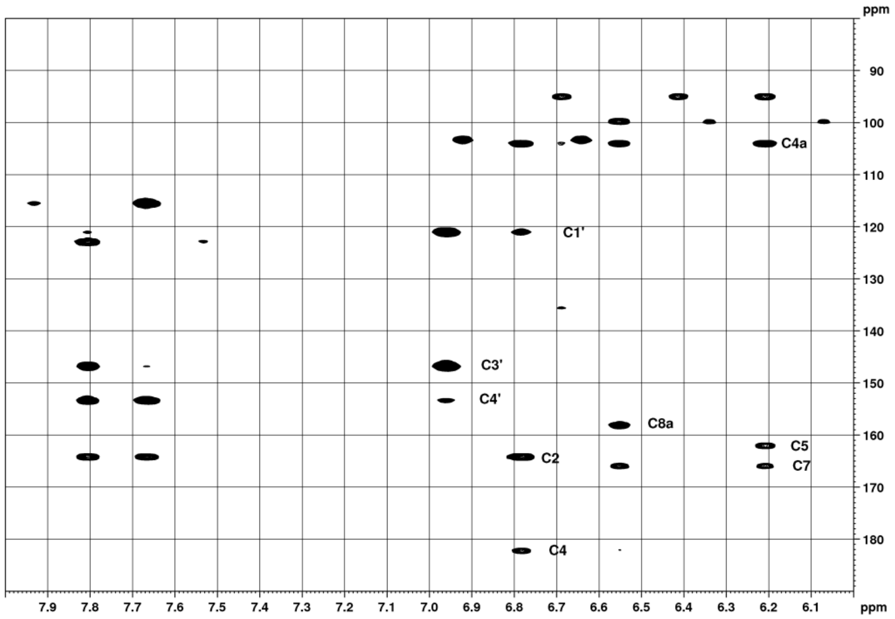

Figure SI 49. HMBC spectrum of **lut**-3′-*O*-glu.

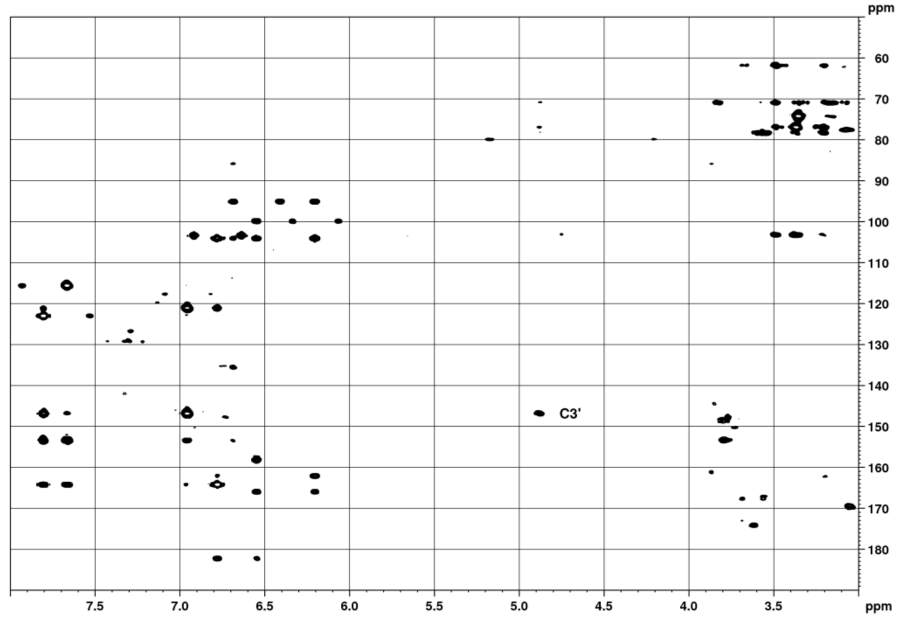

Figure SI 50. HMBC spectrum of **lut**-3′-*O*-glu; glucose signals displayed.

Fig. SI 50 shows a correlation between C-3′ and H-1″. This indicates that the glucose moiety is attached to O-3′.

Apigenin-6,8-di-*C*-β-D-glucoside (**api**-6,8-di-*C*-glu) **1**

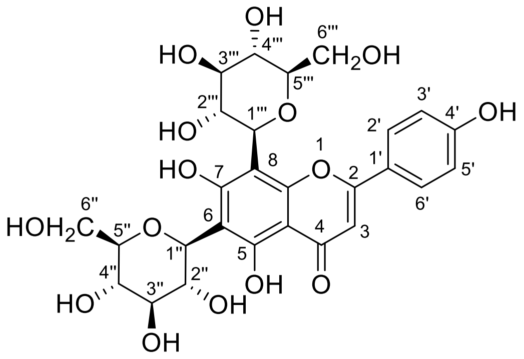

apigenin-6,8-di-C-β-D-glucopyranoside

In DMSO-d_6, severe line broadening was observed. The line-width improved significantly after dilution of the sample with MeOH-d_4. The additional signals and broadening were due to slow conformational averaging, as the 8-hexose obstructs rotation of the B-ring [1]. Better spectra were obtained in DMSO at 320 K. The discussions are based on the DMSO–MeOH spectra at 300 K.

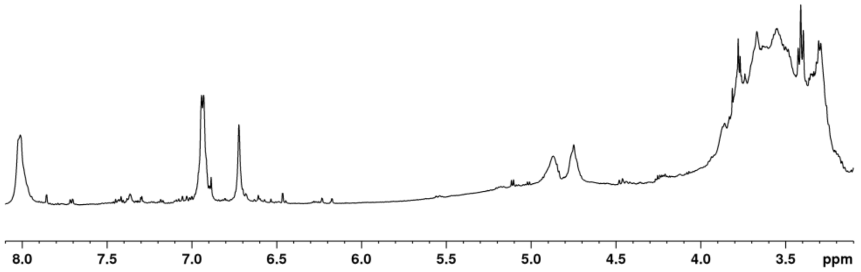

Figure SI 51. ^{1}H-NMR spectrum of **api**-6,8-di-*C*-hex (DMSO-d_6, 300 K).

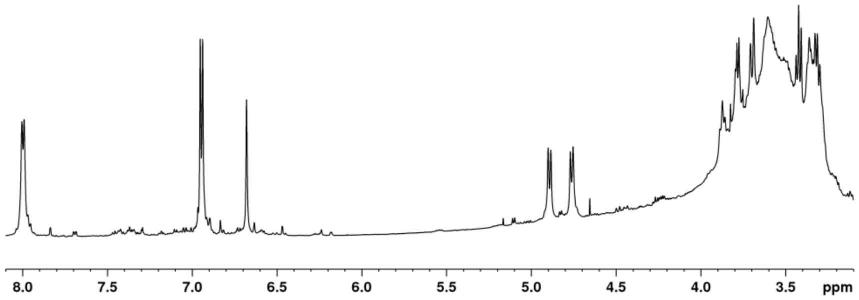

Figure SI 52. ^{1}H-NMR spectrum of **api**-6,8-di-*C*-hex (DMSO-d_6, 320 K).

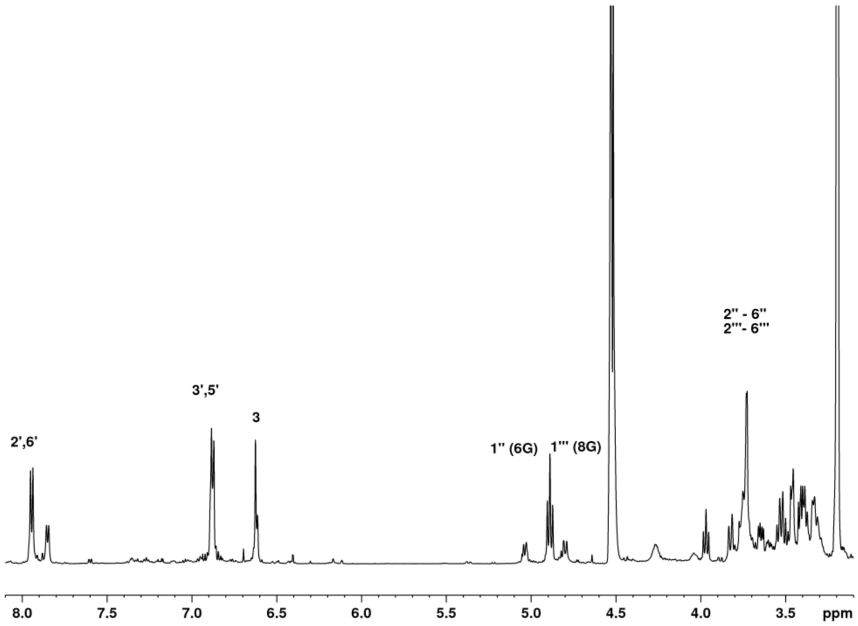

Figure SI 53. ^{1}H-NMR spectrum of **api**-6,8-di-*C*-hex [DMSO-d_6–MeOH-d_4 1:3 (v/v), 300 K].

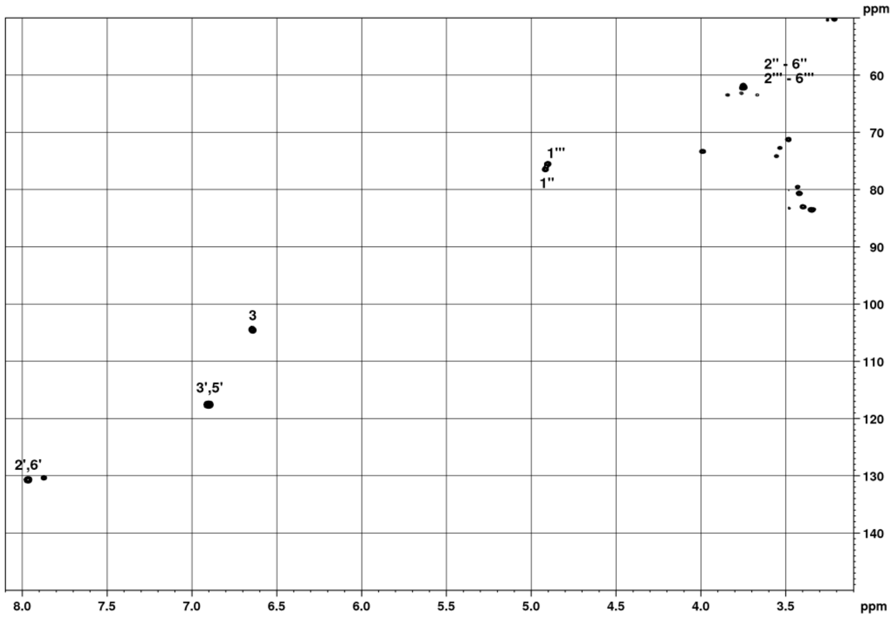

Figure SI 54. HSQC spectrum of **api**-6,8-di-*C*-hex [DMSO-d_6–MeOH-d_4 1:3 (v/v), 300 K].

The absence of H-6 and H-8 signals in the ^{1}H and HSQC spectra suggest that the sugar moieties are attached to C-6, and C-8. The ^{13}C δ-values of the anomeric C-atoms further support this assignment, as their signals appeared at lower frequency than those of the *O*-glycosides (75 *vs.* 100 ppm; Table 1 of manuscript).

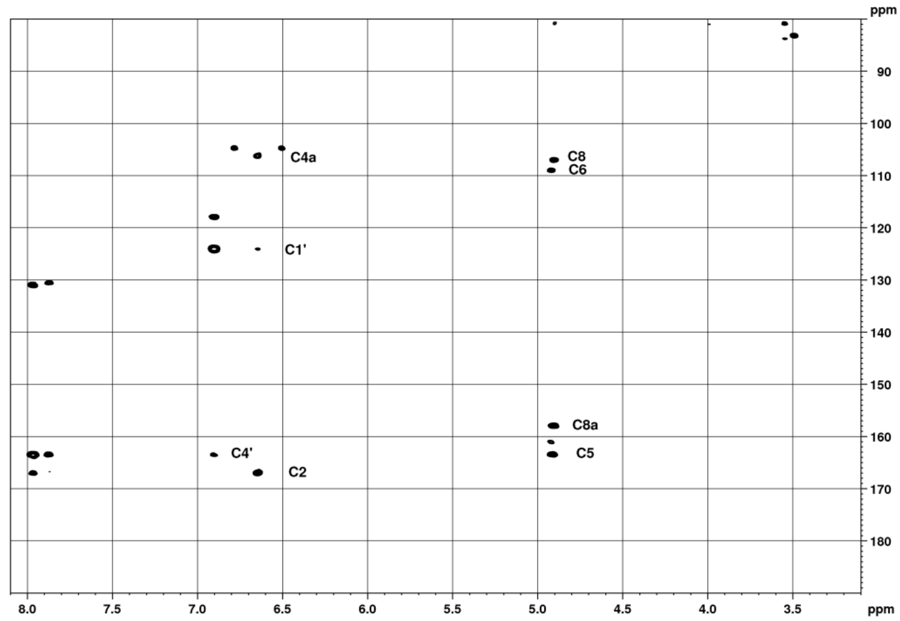

Figure SI 55. HMBC spectrum of **api**-6,8-di-*C*-hex [DMSO-d_6–MeOH-d_4 1:2 (v/v), 300 K].

The signals of the anomeric H-atoms are not well separated in this solvent. Nevertheless, it can be deduced from the HMBC spectrum that the high frequency proton signal is coupled to the C-5 and C-6. Therefore, this proton signal is assigned to the sugar moiety on C-6 (″). In contrast, the low frequency proton signal is coupled to C-8a and C-8 and, therefore, it is assigned to the sugar moiety on C-8 (‴).

Luteolin-7,4′-di-*O*-β-D-glucoside (**lut**-7,4′-di-*O*-glu) **2**

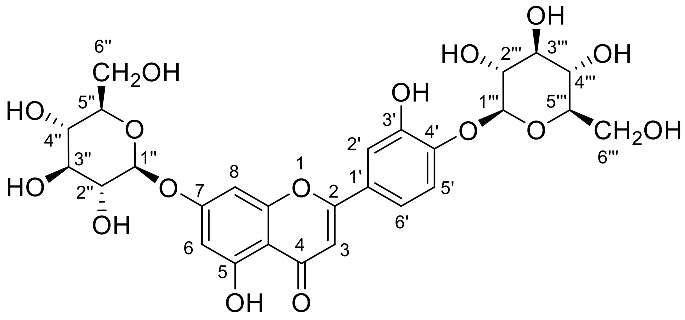

luteolin-7,4'-di-O-β-D-glucopyranoside

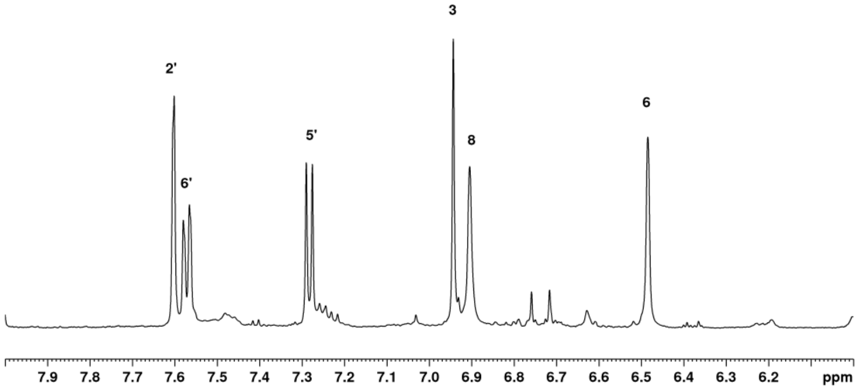

Figure SI 56. ^{1}H-NMR spectrum of **lut**-7,4′-di-*O*-glu.

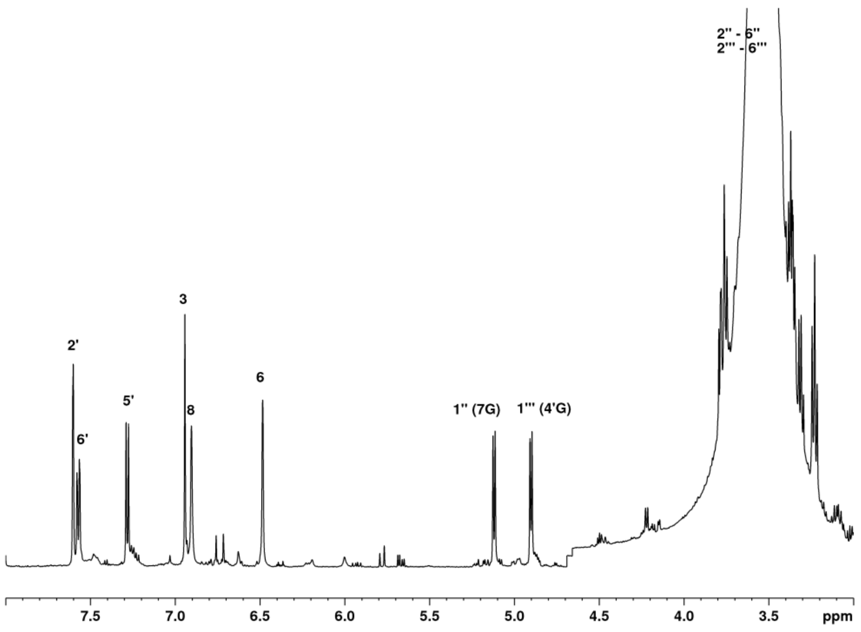

Figure SI 57. ^{1}H-NMR spectrum of **lut**-7,4′-di-*O*-glu; glucose signals displayed.

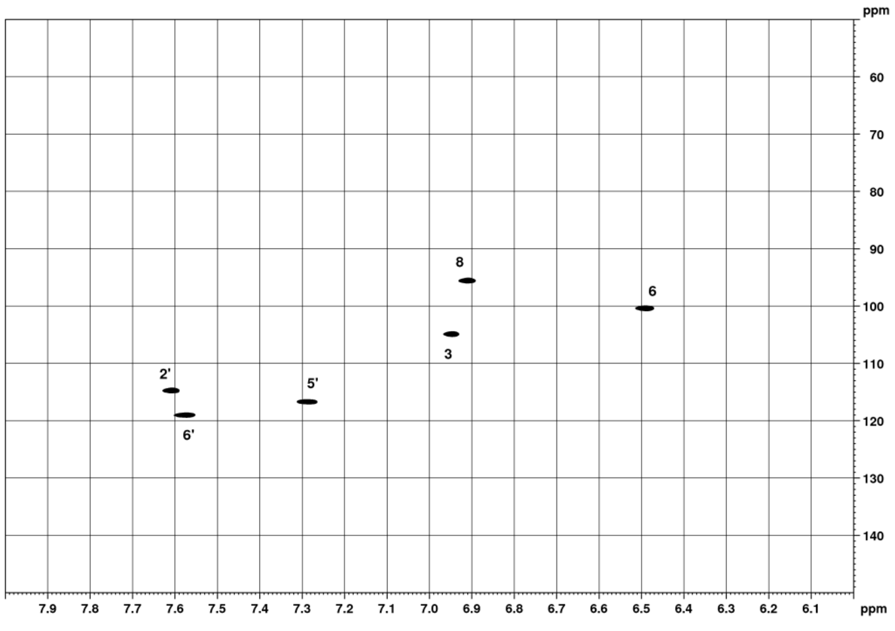

Figure SI 58. HSQC spectrum of **lut**-7,4′-di-*O*-glu.

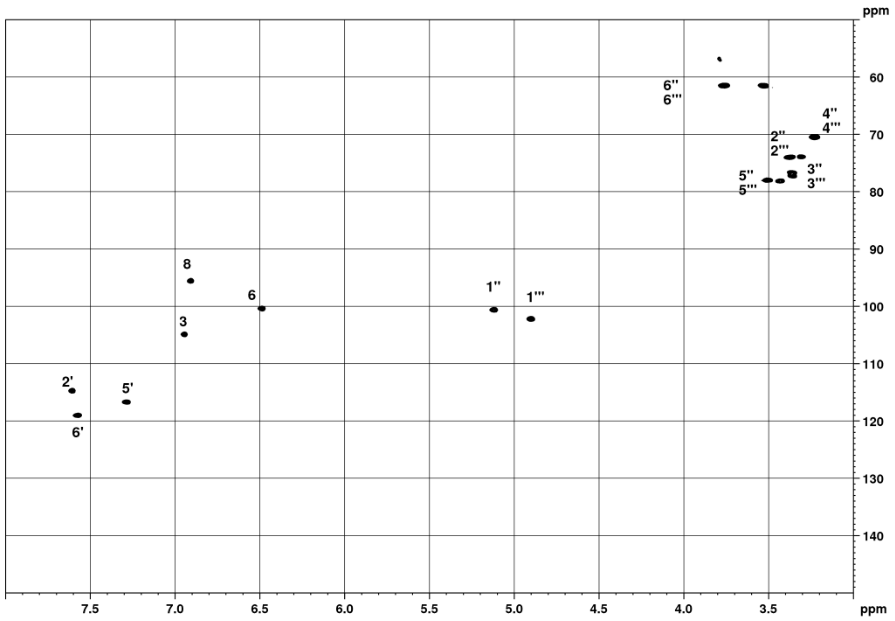

Figure SI 59. HSQC spectrum of **lut**-7,4′-di-*O*-glu; glucose signals displayed.

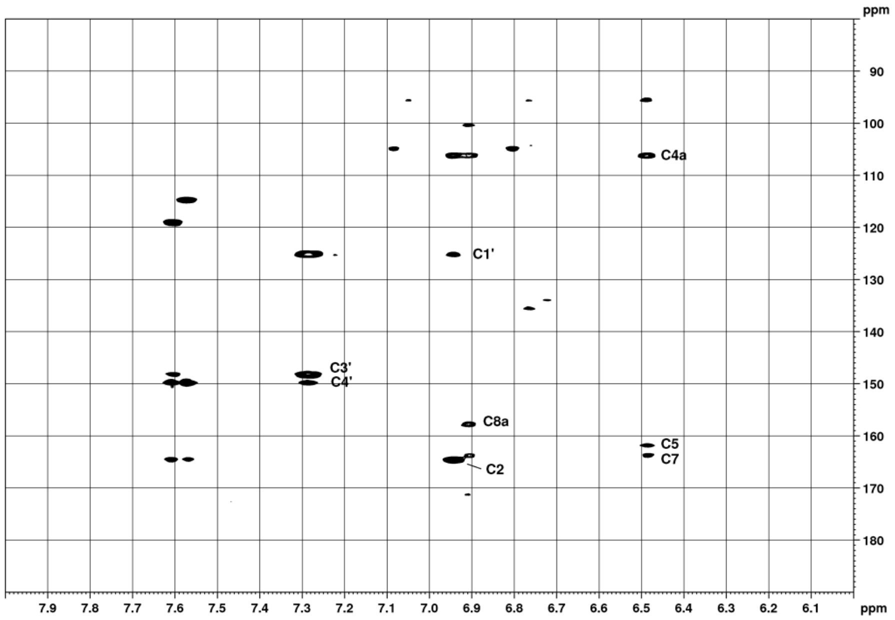

Figure SI 60. HMBC spectrum of **lut**-7,4′-di-*O*-glu.

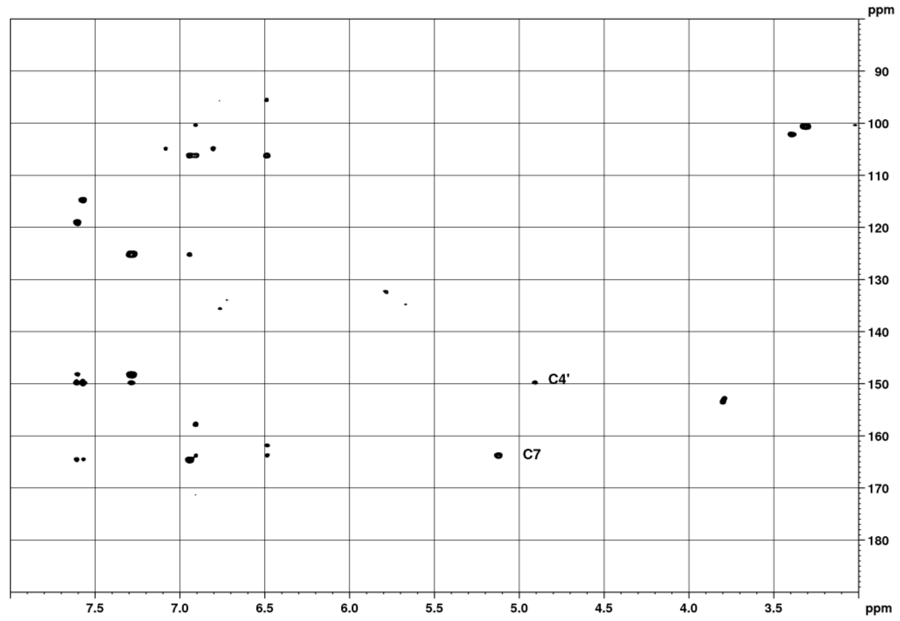

Figure SI 61. HMBC spectrum of **lut**-7,4′-di-*O*-glu; glucose signals displayed.

Fig. SI 61 shows a correlation between C-7 and H-1″. This confirms that the glucose ″ is attached to O-7. It also shows a correlation between C-4′ and H-1‴. This confirms that the glucose ‴ is attached to O-4′.

Luteolin-7,3′-di-*O*-β-D-glucoside (**lut**-7,3′-di-*O*-glu) **3**

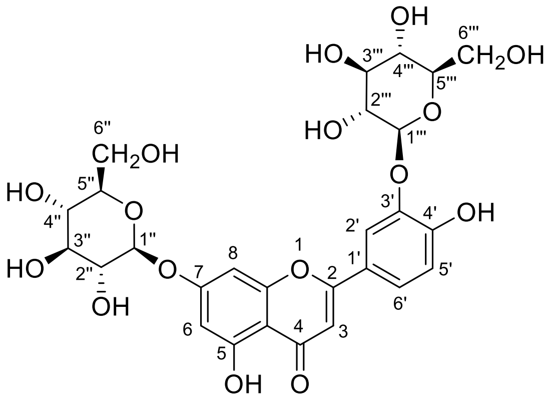

luteolin-7,3'-di-O-β-D-glucopyranoside

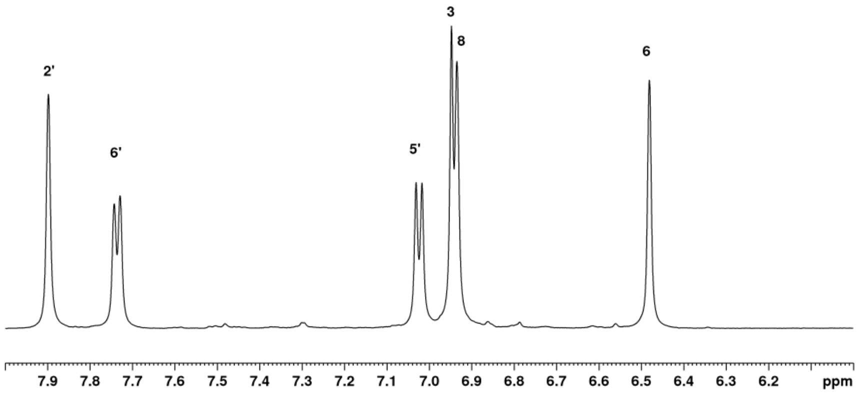

Figure SI 62. ^{1}H-NMR spectrum of **lut**-7,3′-di-*O*-glu.

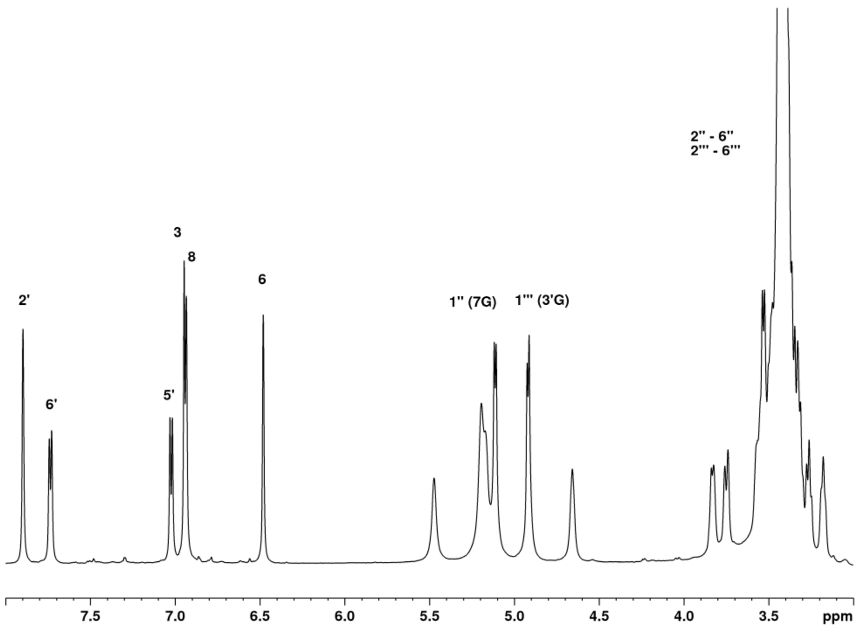

Figure SI 63. ^{1}H-NMR spectrum of **lut**-7,3′-di-*O*-glu; glucose signals displayed.

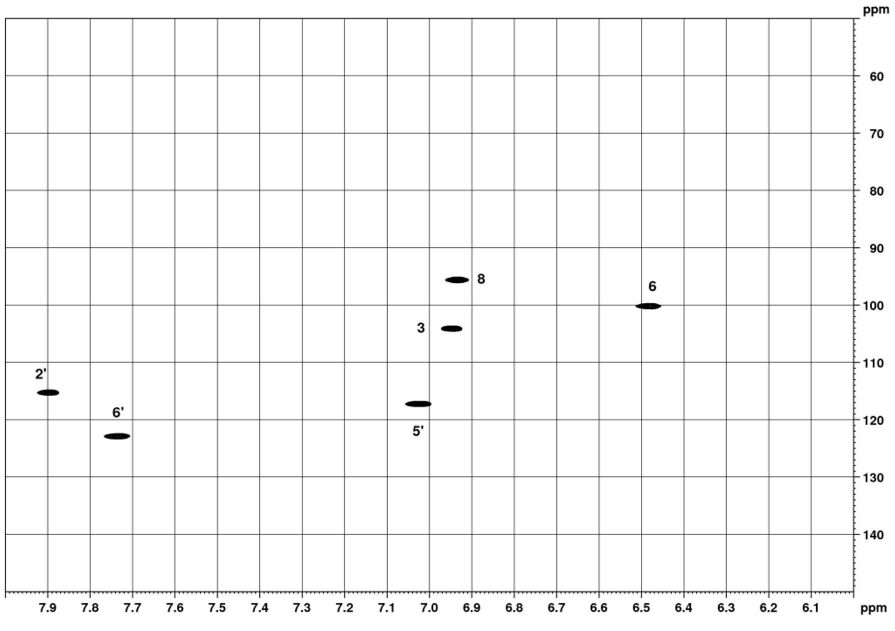

Figure SI 64. HSQC spectrum of **lut**-7,3′-di-*O*-glu.

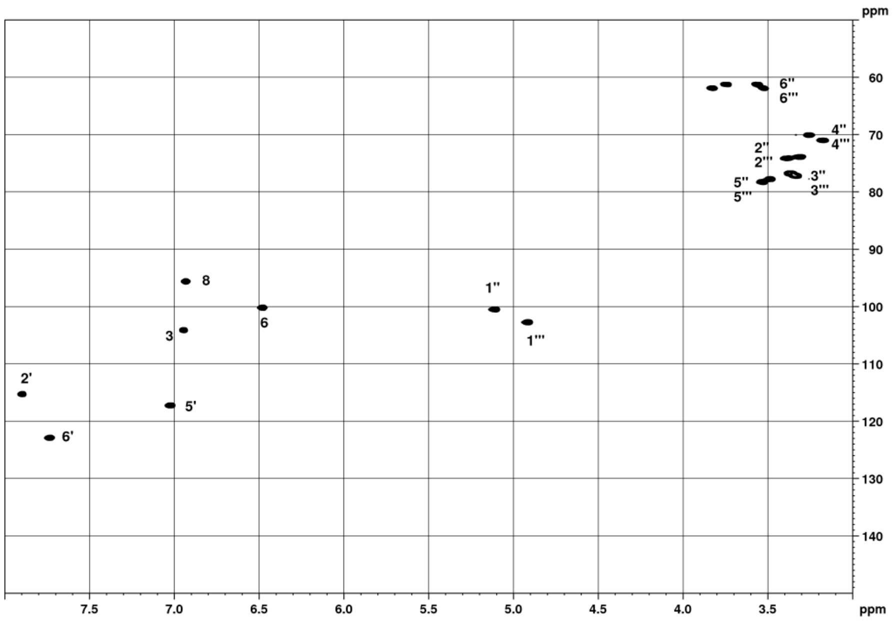

Figure SI 65. HSQC spectrum of **lut**-7,3′-di-*O*-glu; glucose signals displayed.

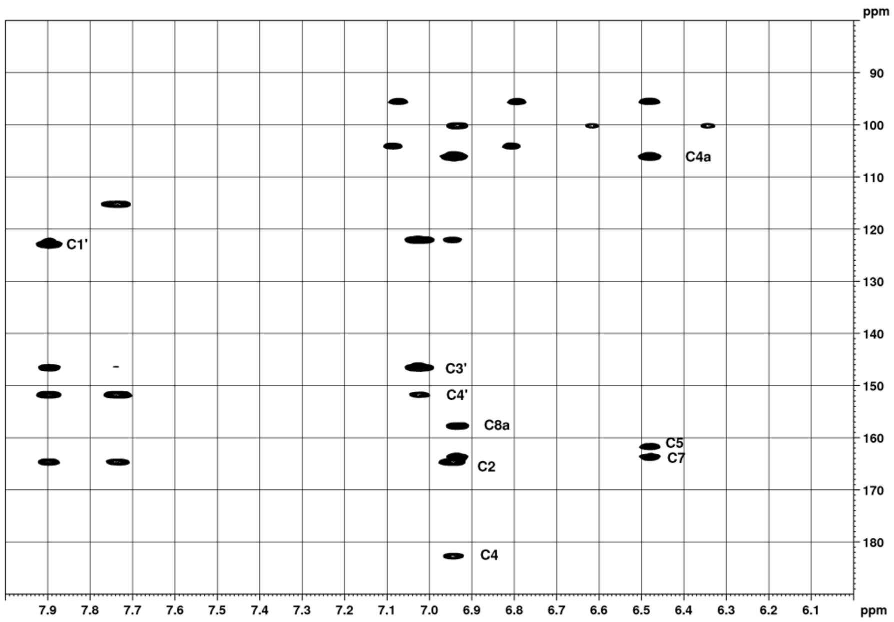

Figure SI 66. HMBC spectrum of **lut**-7,3′-di-*O*-glu.

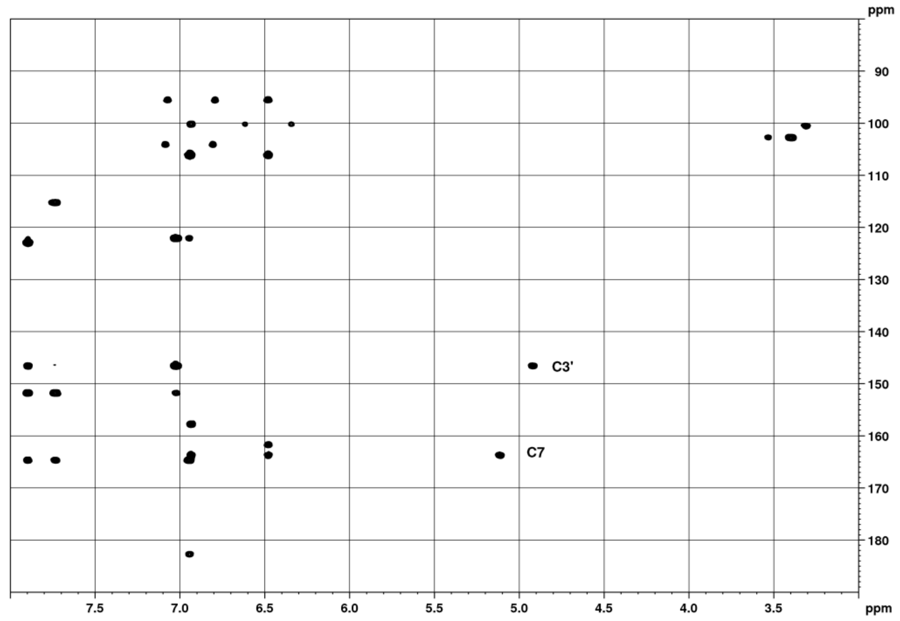

Figure SI 67. HMBC spectrum of **lut**-7,3′-di-*O*-glu; glucose signals displayed.

Fig. SI 67 shows a correlation between C-7 and H-1″. This confirms that the glucose ″ is attached to O-7. It also shows a correlation between C-3′ and H-1‴. This confirms that the glucose ‴ is attached to O-3′.

Reference

1. Markham, K.R.; Geiger, H. ^{1}H Nuclear magnetic resonance spectroscopy of flavonoids and their glycosides in hexadeuterodimethylsulfoxide. In *The Flavonoids: Advances in Research since 1986*, 1st ed.; Harborne, J.B., Ed.; Chapman & Hall: London, UK, 1994; pp 441–497.

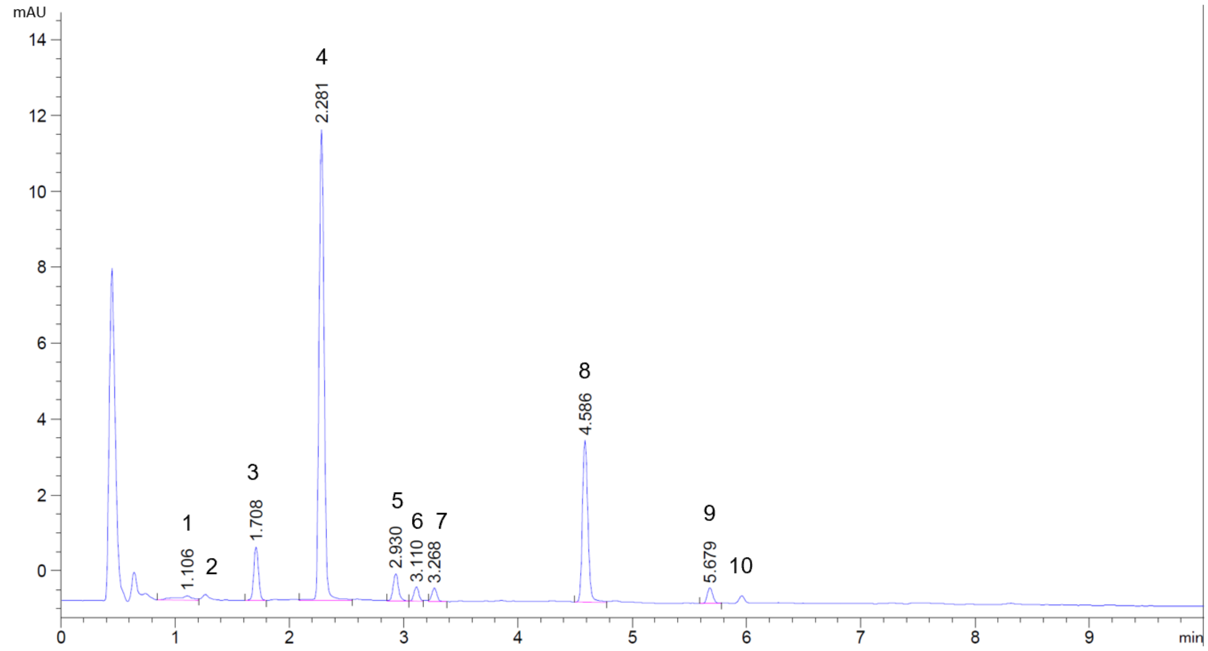

Fig. SI 68. UHPLC profile of a duplicate injection of an extract of 56.6 µg weld-dyed wool (compare with Fig. 4a in paper proper).

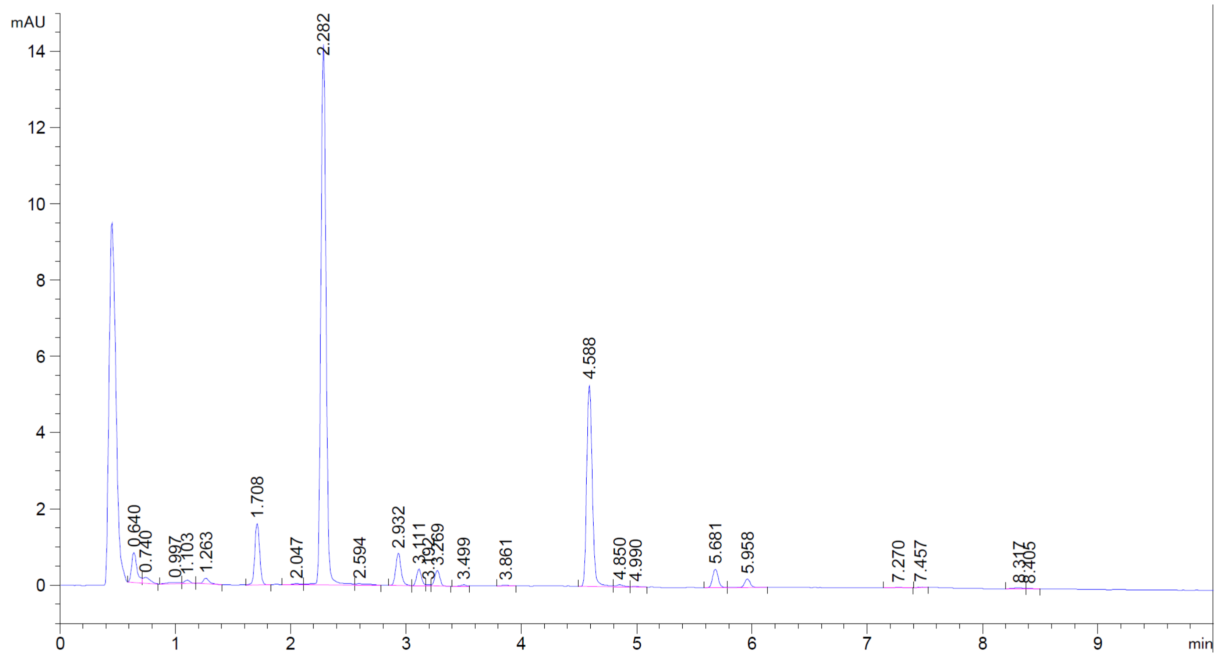

Fig. SI 69. UHPLC profile of a duplicate injection of an extract of 49.2 µg weld-dyed wool (compare with Fig. 4b in paper proper).

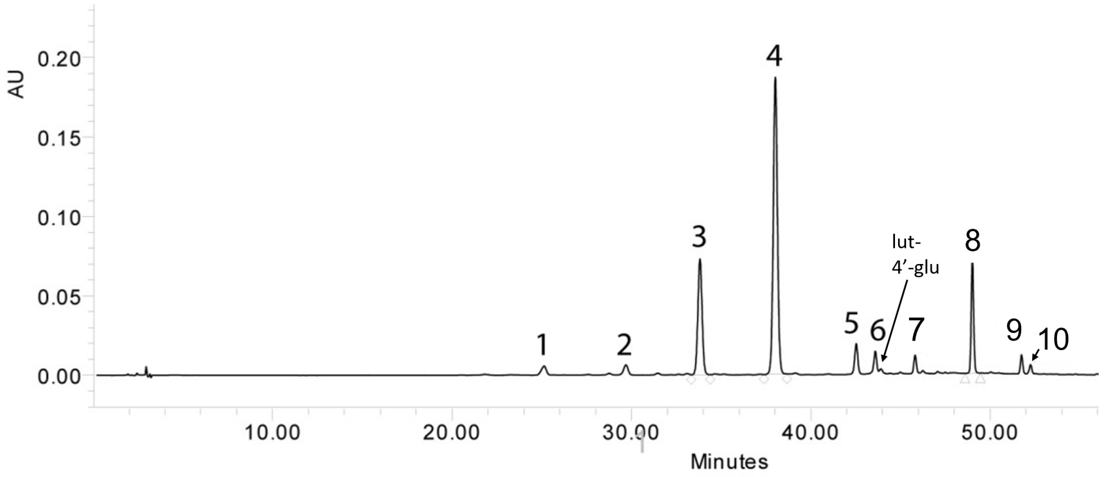

Fig. SI 70. HPLC profile of weld extract including the barely visible peak of **lut**-4'-glu **tr2**, which is not visible in the UHPLC profile (Fig. 2 of the paper proper). For conditions, see: Villela et al., *J. Chromatogr. A* **2011**, *1218*, 8544-8550 (2011).

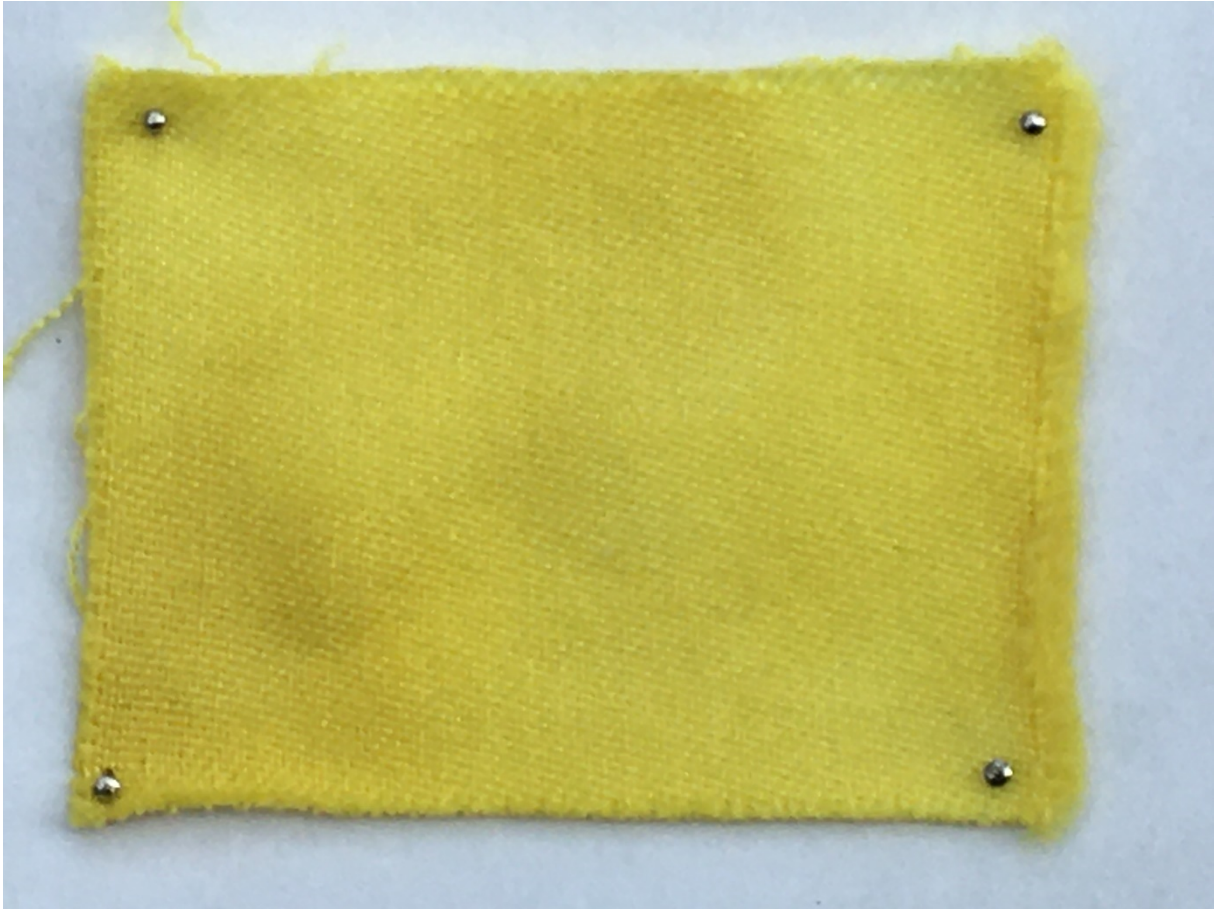

Fig. SI 71. Example of wool dyed in 2011 with weld. Threads were used in this investigation.

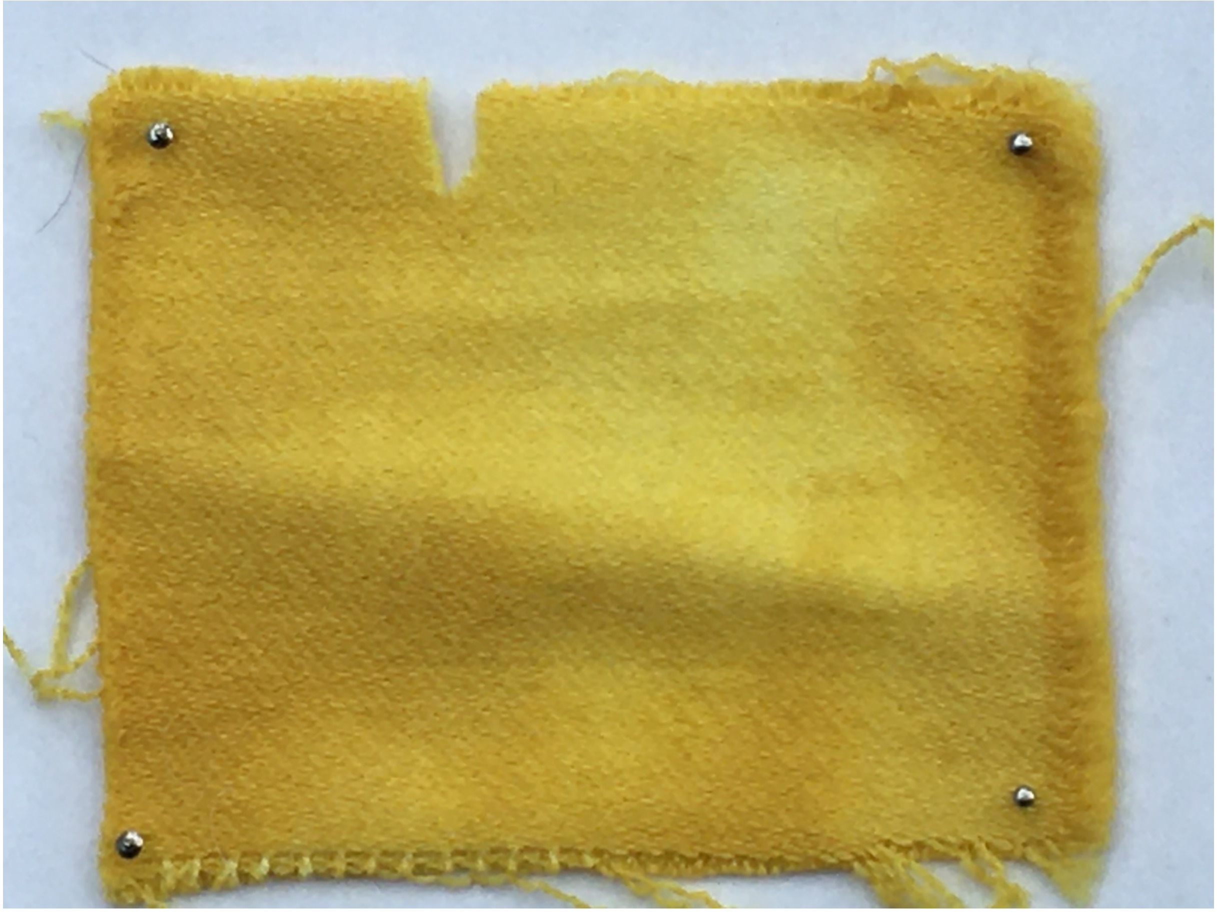

Fig. SI 72. Example of wool dyed in 2011 with onion. Threads were used in this investigation.

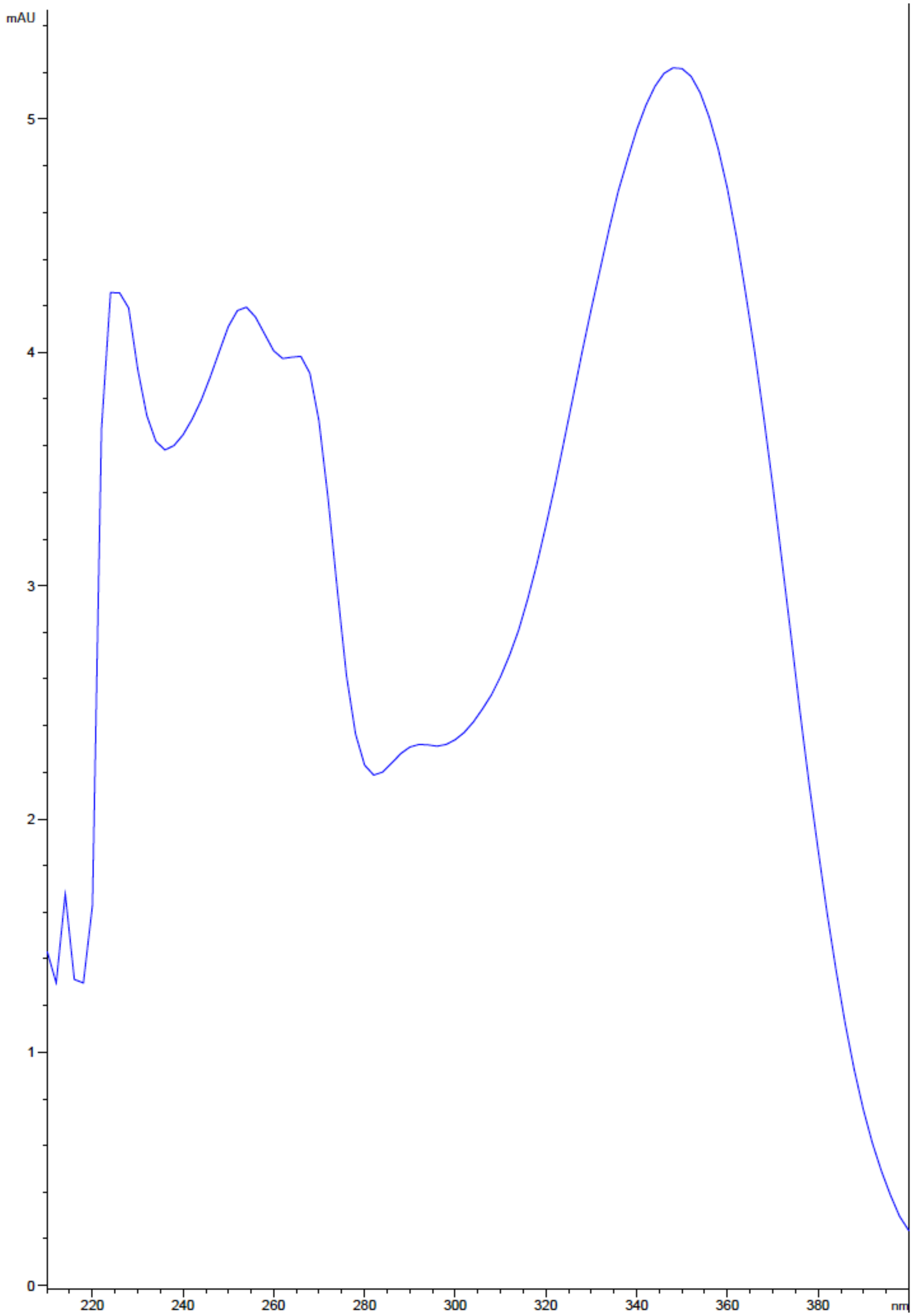

Fig. SI 73. On-line UV spectrum of peak **8** (luteolin) of UHPLC analysis of an extract of 49.2 µg weld-dyed wool (compare with Fig. 4b in paper proper).

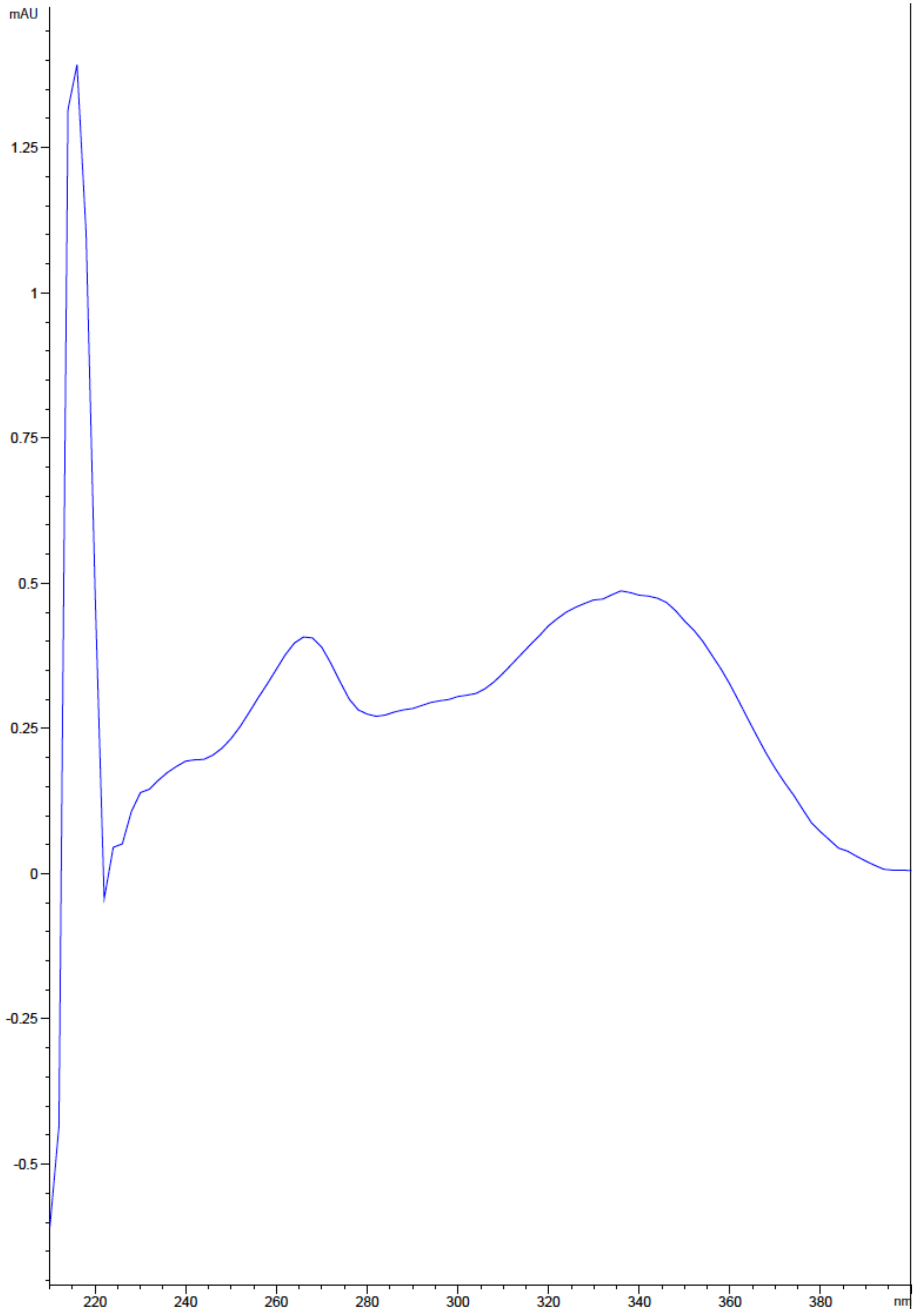

Fig. SI 74. On-line UV spectrum of peak **9** (apigenin of UHPLC analysis of an extract of 49.2 µg weld-dyed wool (compare with Fig. 4b in paper proper).

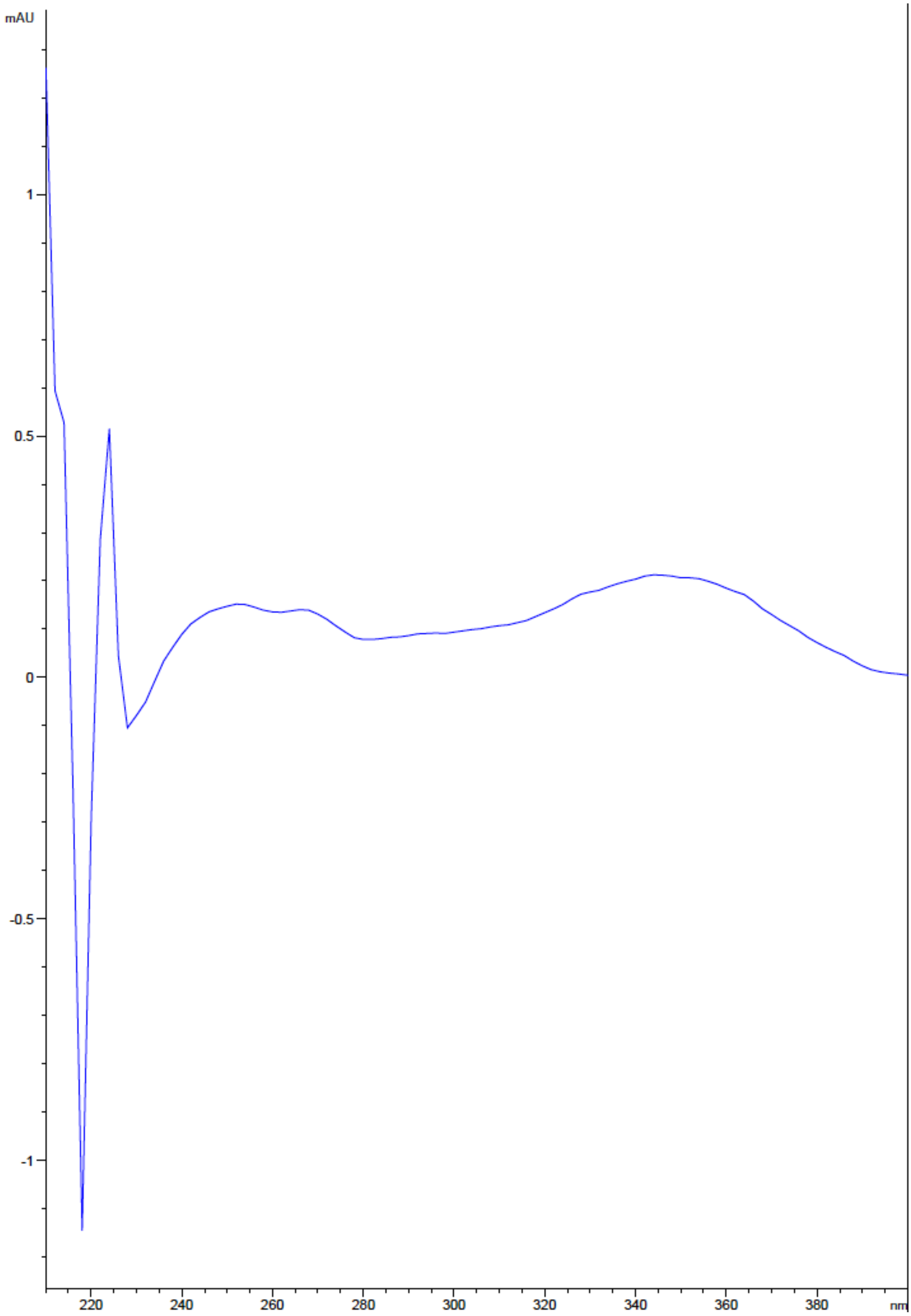

Fig. SI 75. On-line UV spectrum of peak **10** (chrysoeriol) of UHPLC analysis of an extract of 49.2 µg weld-dyed wool (compare with Fig. 4b in paper proper).

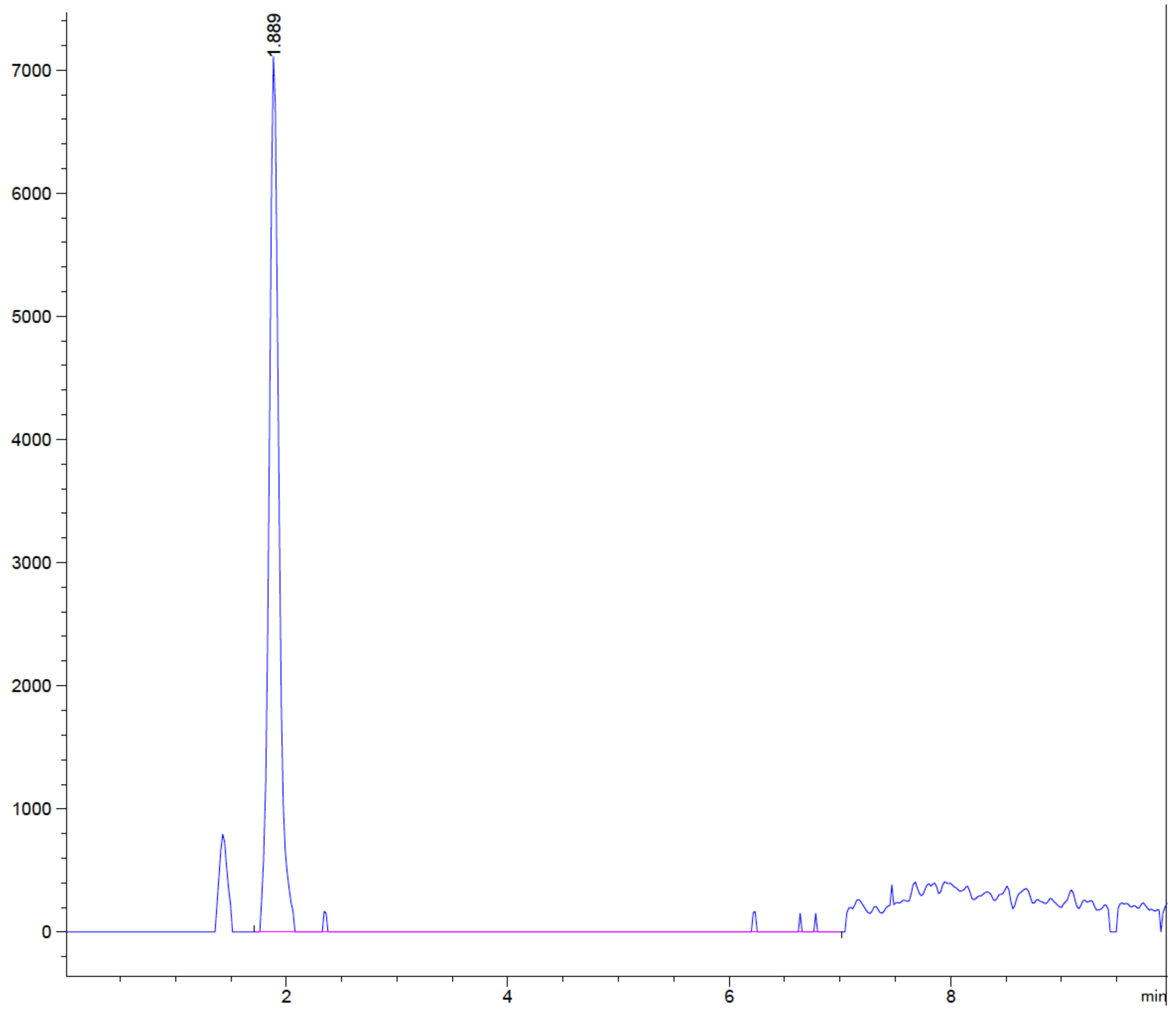

Fig. SI 76. Extracted ion chromatogram (EIC) of *m/z* 609 $[M{-}H]^-$ of UHPLC-DAD-MS analysis of weld extract (compare with Fig. 2 in paper proper). Time difference between UV and MS detection ~0.159 min.

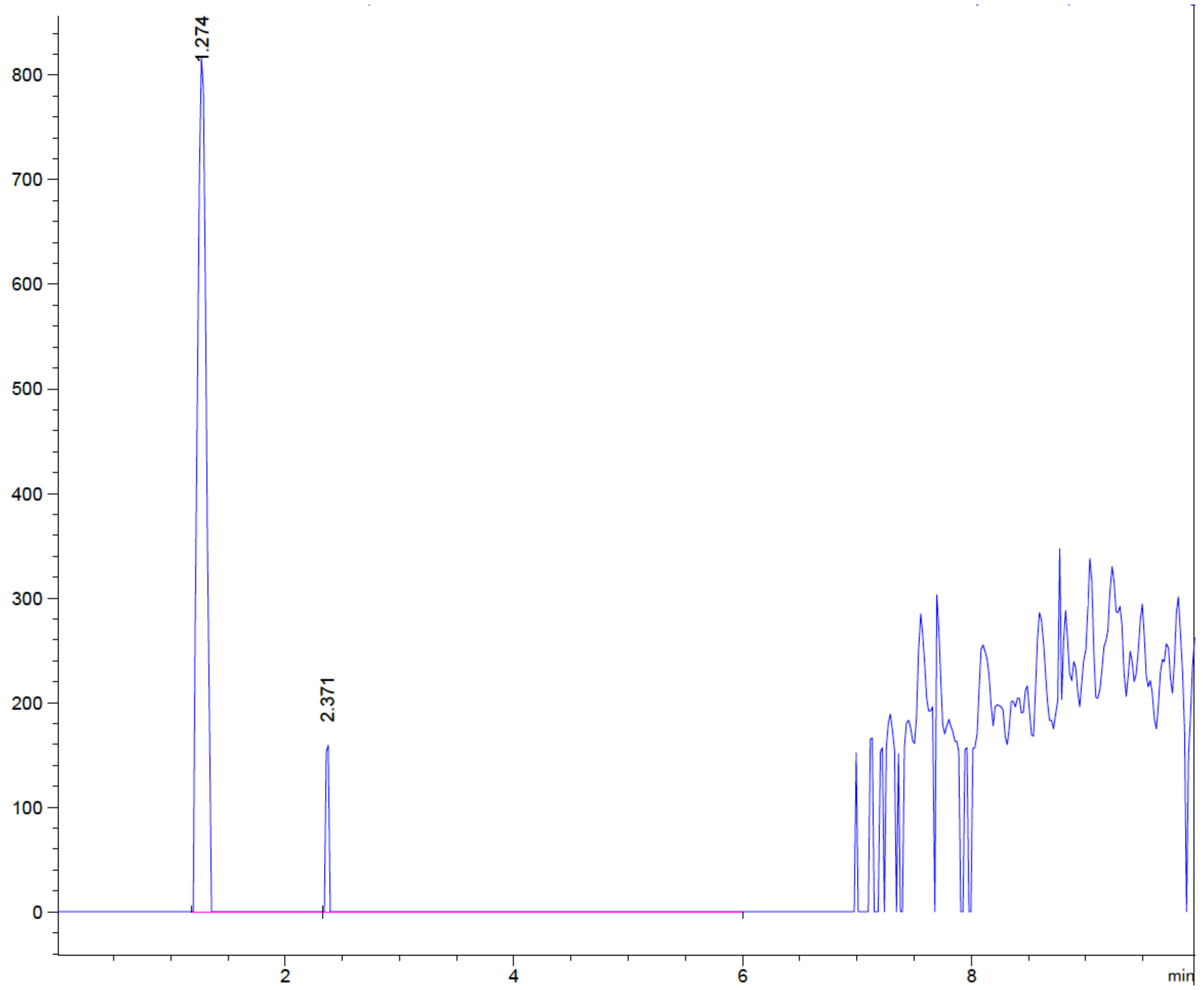

Fig. SI 77. Extracted ion chromatogram (EIC) of *m/z* 593 $[M{-}H]^-$ of UHPLC-DAD-MS analysis of weld extract (compare with Fig. 2 in paper proper). Time difference between UV and MS detection ~0.159 min.

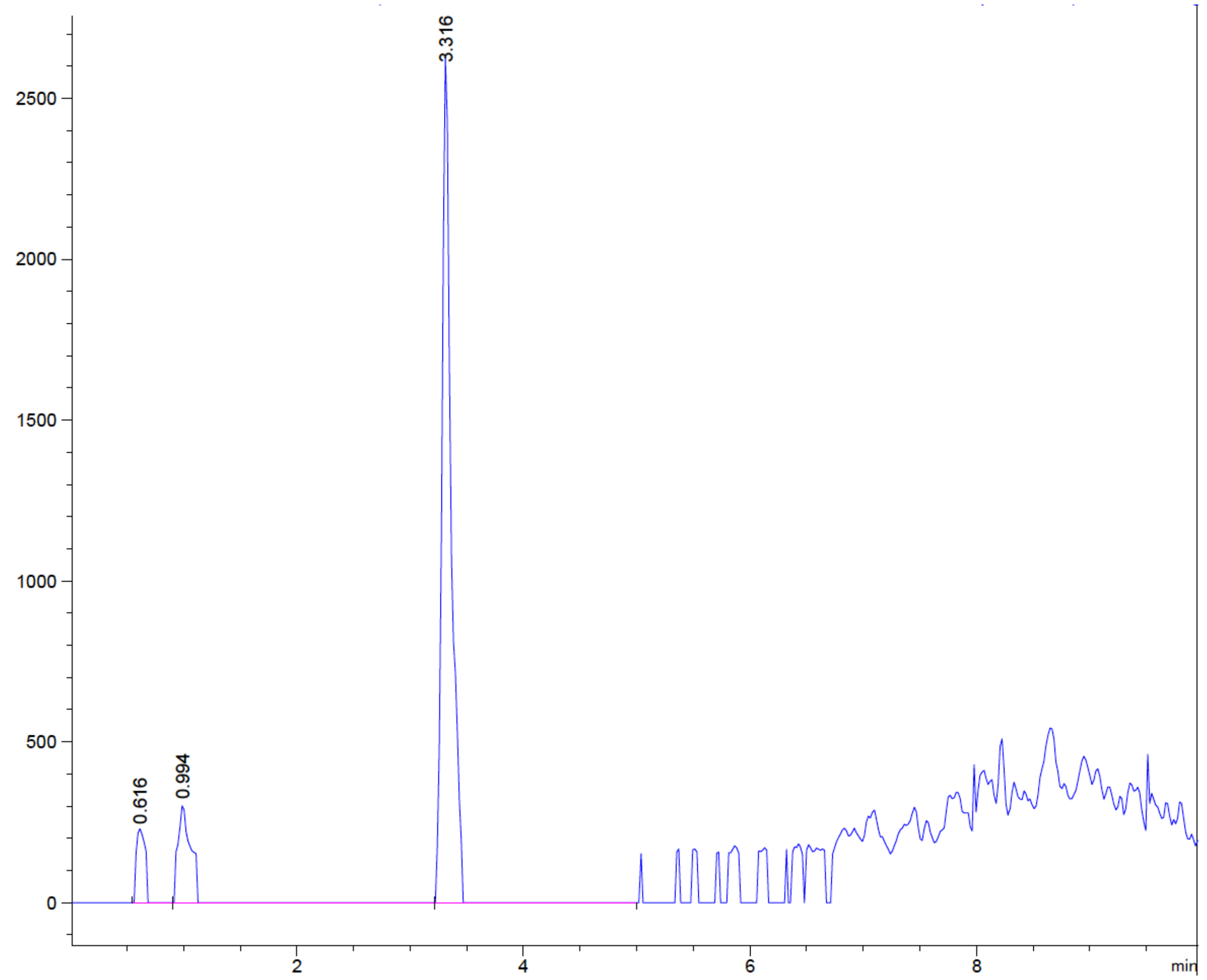

Fig. SI 78. Extracted ion chromatogram (EIC) of *m/z* 461 $[M{-}H]^{-}$ of UHPLC-DAD-MS analysis of weld extract (compare with Fig. 2 in paper proper). Time difference between UV and MS detection ~0.159 min.

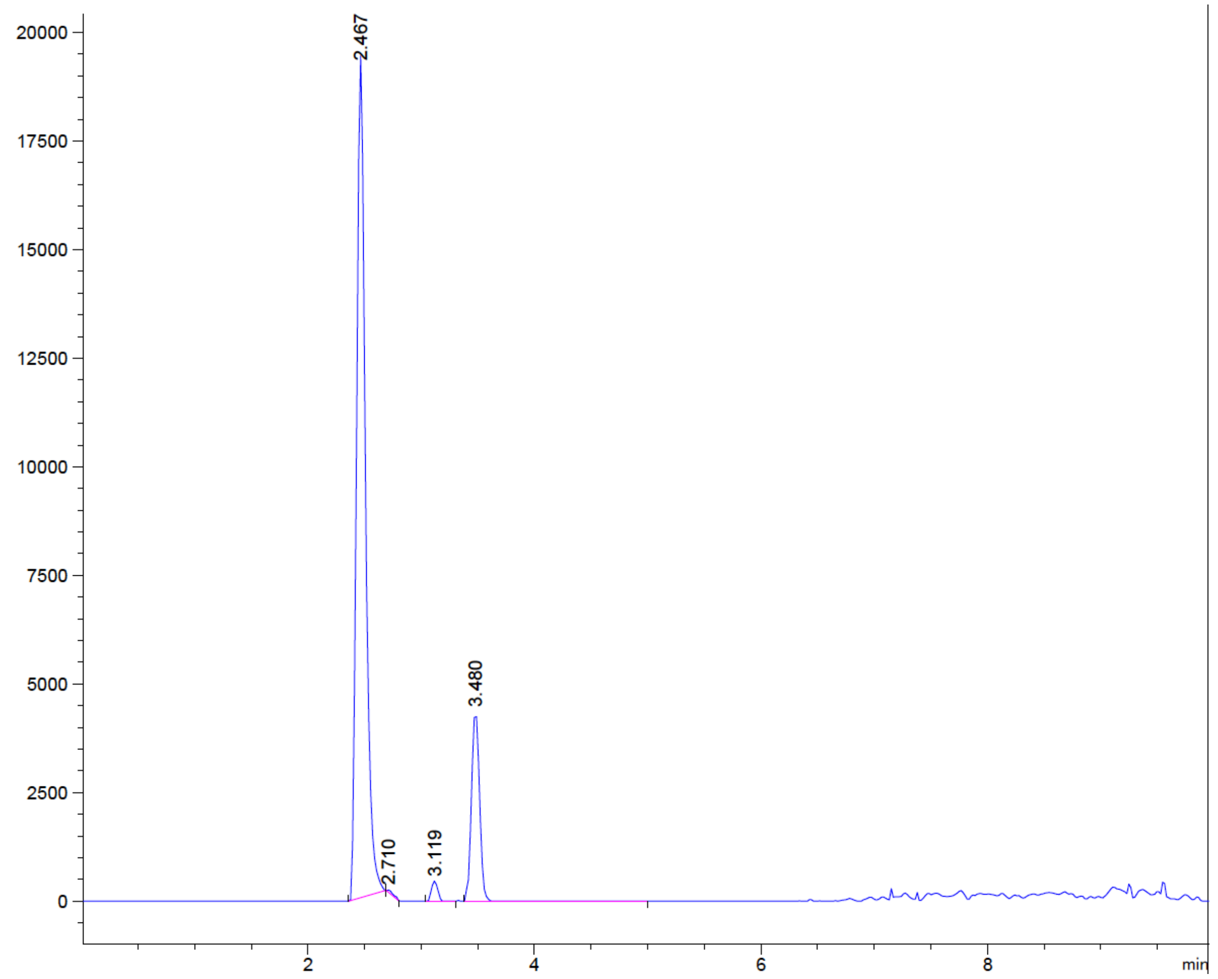

Fig. SI 79. Extracted ion chromatogram (EIC) of *m/z* 447 $[M{-}H]^-$ of UHPLC-DAD-MS analysis of weld extract (compare with Fig. 2 in paper proper). Time difference between UV and MS detection ~0.159 min.

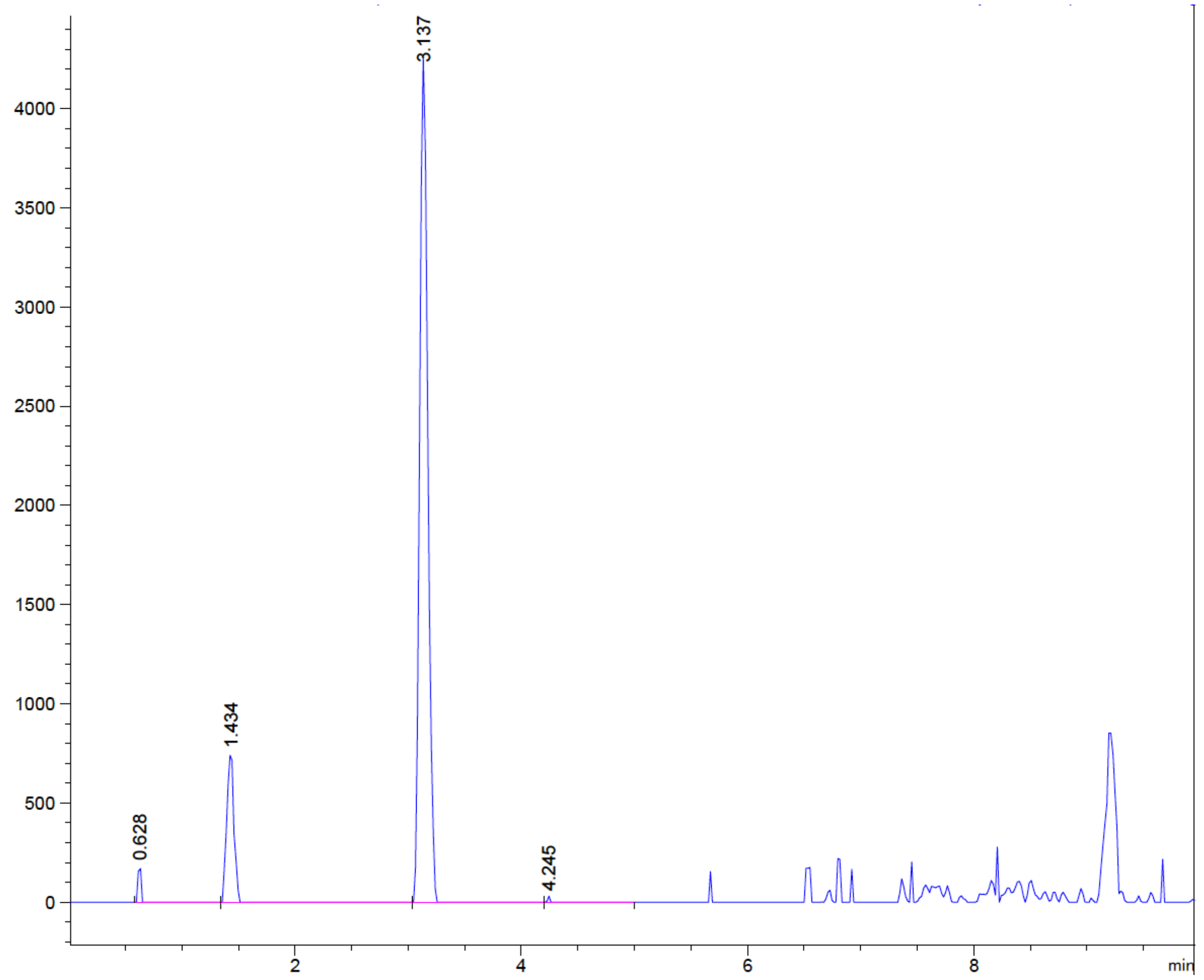

Fig. SI 80. Extracted ion chromatogram (EIC) of *m/z* 431 $[M{-}H]^-$ of UHPLC-DAD-MS analysis of weld extract (compare with Fig. 2 in paper proper). Time difference between UV and MS detection ~0.159 min.

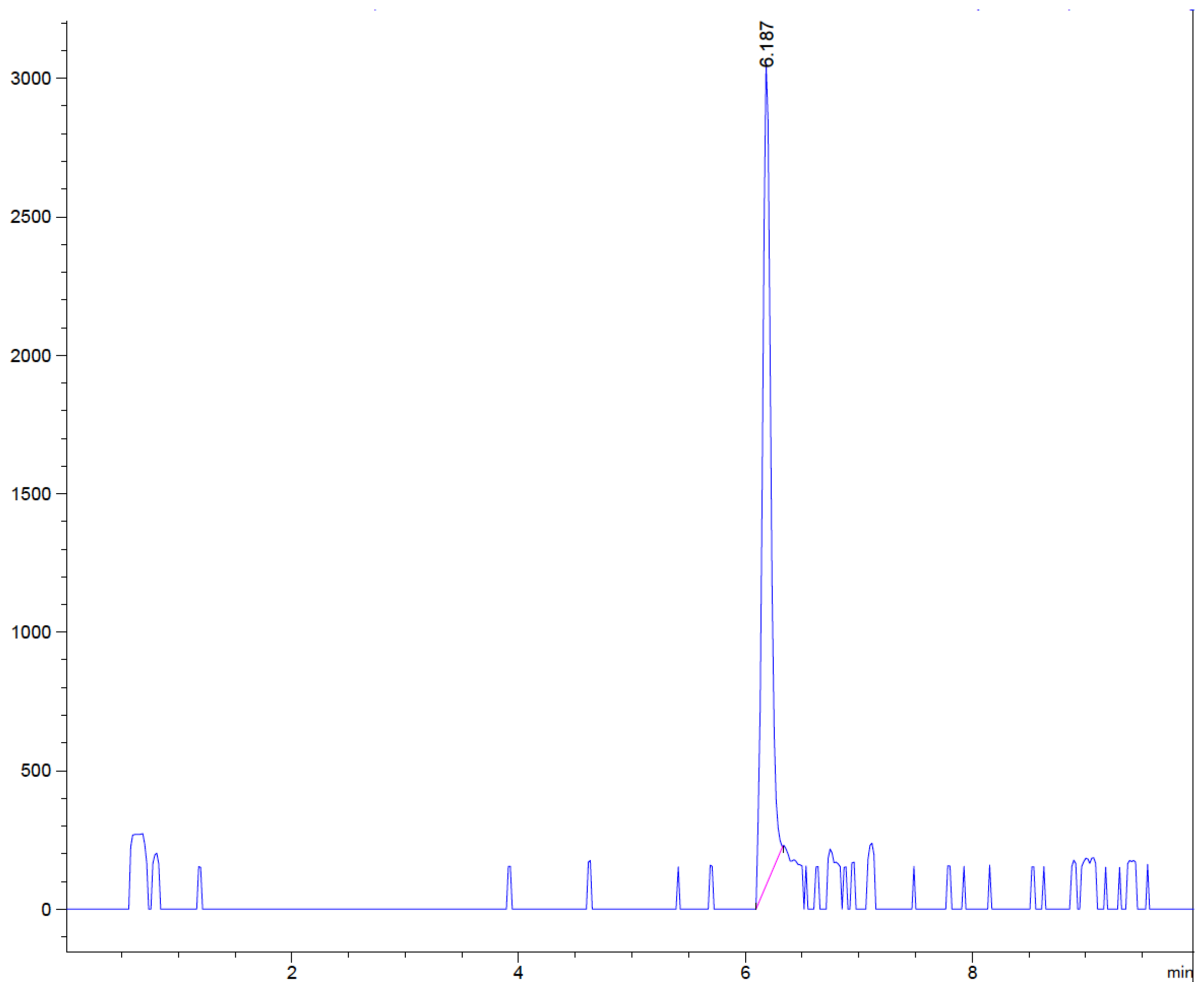

Fig. SI 81. Extracted ion chromatogram (EIC) of *m/z* 299 [M−H]⁻ of UHPLC-DAD-MS analysis of weld extract (compare with Fig. 2 in paper proper). Time difference between UV and MS detection ~0.159 min.

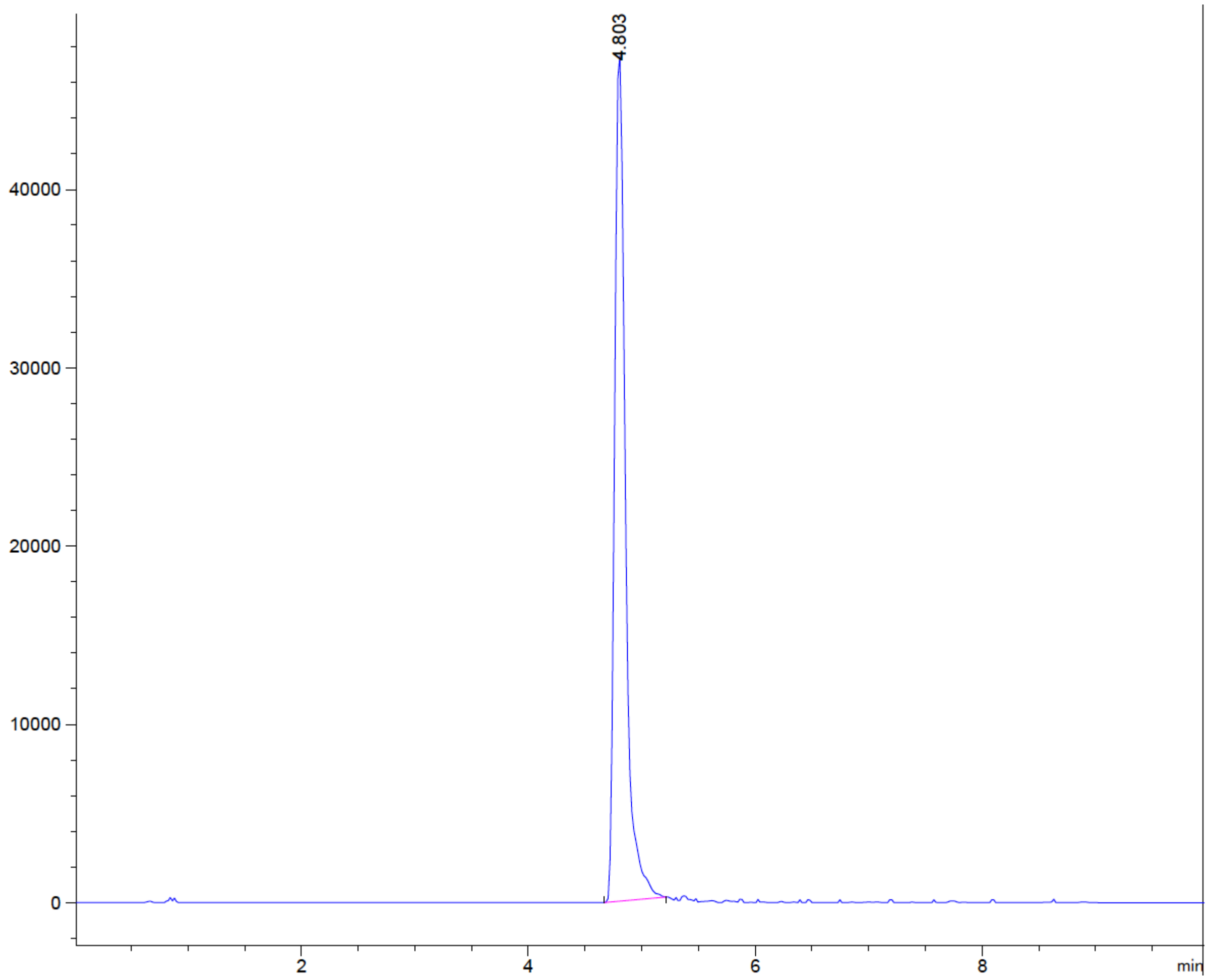

Fig. SI 82. Extracted ion chromatogram (EIC) of *m/z* 285 $[M-H]^-$ of UHPLC-DAD-MS analysis of weld extract (compare with Fig. 2 in paper proper). Time difference between UV and MS detection ~0.159 min.

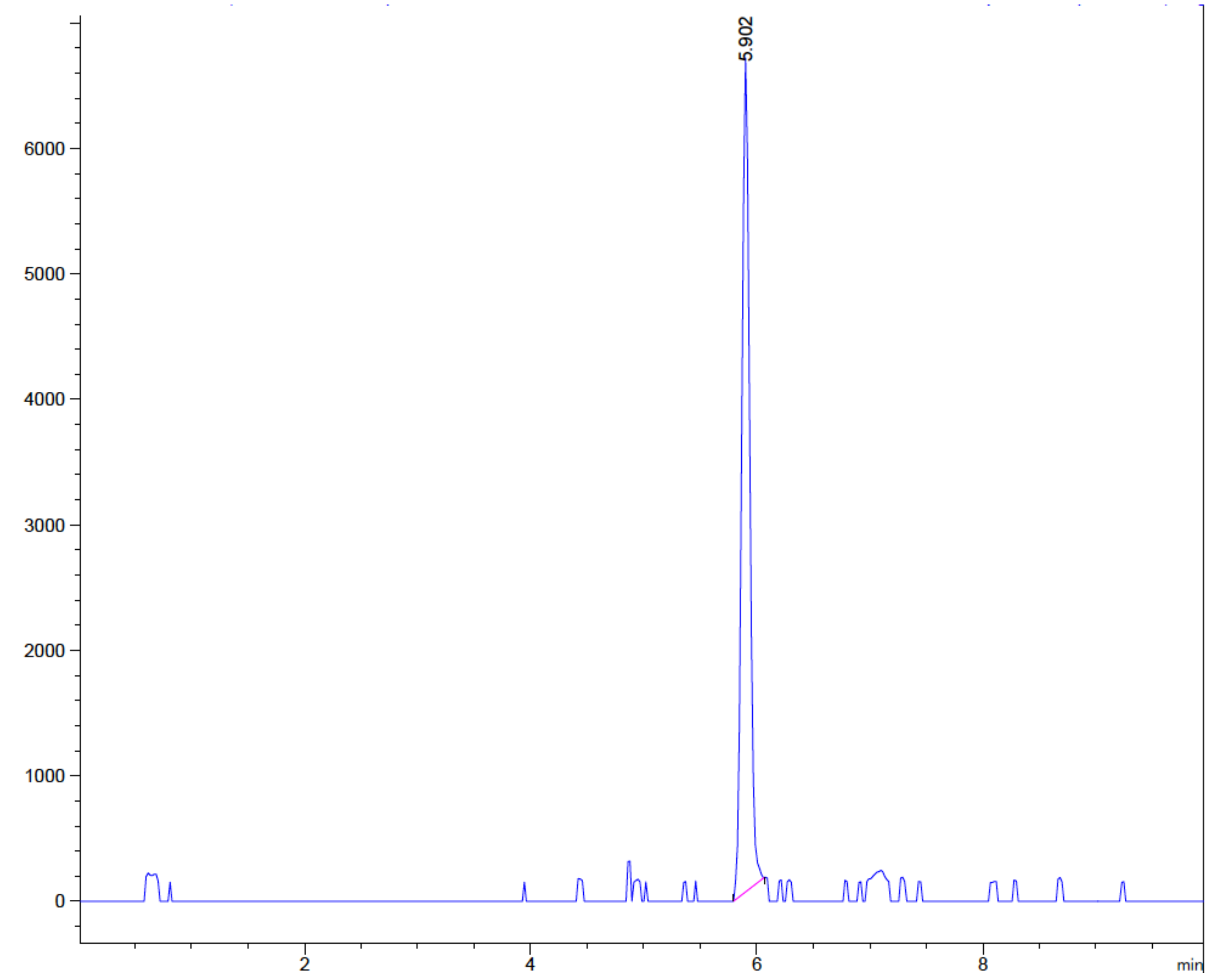

Fig. SI 83. Extracted ion chromatogram (EIC) of *m/z* 269 $[M{-}H]^-$ of UHPLC-DAD-MS analysis of weld extract (compare with Fig. 2 in paper proper). Time difference between UV and MS detection ~0.159 min.

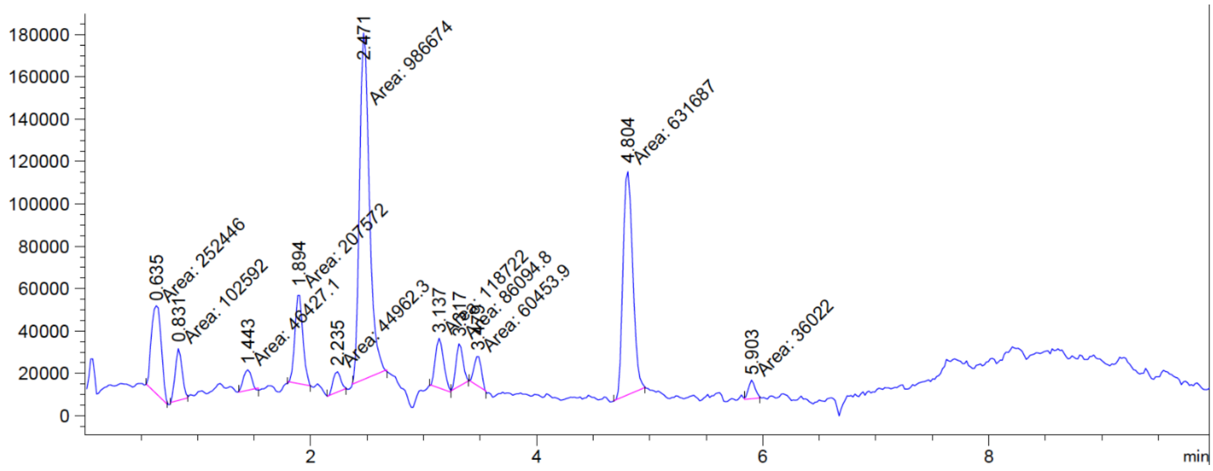

Fig. SI 84. Total ion current (TIC) chromatogram (negative mode) of UHPLC-DAD-MS analysis of weld extract (compare with Fig. 2 in paper proper). Time difference between UV and MS detection ~0.159 min.

Table SI 1: High resolution mass spectrometric measurements of the twelve isolated weld flavones.

Peak numbering [a]	Identity of the flavone	Molecular formula	$[M-H]^-$ theoretical mass (amu)	Measured mass (amu) (relative intensity, in %)
**1**	**api**-6,8-di-*C*-glu	$C_{27}H_{30}O_{15}$	593.1501	593.1514 (34) [c]
**2**	**lut**-7,4′-di-*O*-glu	$C_{27}H_{30}O_{16}$	609.1450	609.1467 (100)
**3**	**lut**-7,3′-di-*O*-glu	$C_{27}H_{30}O_{16}$	609.1450	609.1467 (100)
**4**	**lut**-7-*O*-glu	$C_{21}H_{20}O_{11}$	447.0922	447.0935 (100)
**5**	**api**-7-*O*-glu	$C_{21}H_{20}O_{10}$	431.0973	431.0980 (100)
**tr1** [b]	**api**-4′-*O*-glu	$C_{21}H_{20}O_{10}$	431.0973	431.0983 (100)
**tr2** [b]	**lut**-4′-*O*-glu	$C_{21}H_{20}O_{11}$	447.0922	447.0937 (40) [d]
**6**	**chry**-7-*O*-glu	$C_{22}H_{22}O_{11}$	461.1078	461.1088 (100)
**7**	**lut**-3′-*O*-glu	$C_{21}H_{20}O_{11}$	447.0922	447.0935 (100)
**8**	**lut**	$C_{15}H_{10}O_6$	285.0394	285.0404 (100)
**9**	**api**	$C_{15}H_{10}O_5$	269.0444	269.0457 (100)
**10**	**chry**	$C_{16}H_{12}O_6$	299.0550	299.0564 (100)

[a] as in Figure 2 of paper proper; [b] trace flavone co-eluting with **5**; [c] 2nd most intense ion of spectrum, base peak being *m/z* 339.2328 (possibly $C_{23}H_{31}O_2$); [d] 2nd most intense ion of spectrum, base peak being *m/z* 285.0407 (assigned as [**lut**−H]⁻).
